# Supplementary material for: Multimodal profiling of lung granulomas in macaques reveals cellular correlates of tuberculosis control
Source: Immunity. 2022 May 10;55(5):827–846.e10. doi: 10.1016/j.immuni.2022.04.004 (PMC9122264; doi:10.1016/j.immuni.2022.04.004)
Supplement: Document S1. Figures S1–S6 [file mmc1.pdf]

Immunity, Volume *55*

Supplemental information

Multimodal profiling of lung granulomas in macaques reveals cellular correlates of tuberculosis control

Hannah P. Gideon, Travis K. Hughes, Constantine N. Tzouanas, Marc H. Wadsworth II, Ang Andy Tu, Todd M. Gierahn, Joshua M. Peters, Forrest F. Hopkins, Jun-Rong Wei, Conner Kummerlowe, Nicole L. Grant, Kievershen Nargan, Jia Yao Phuah, H. Jacob Borish, Pauline Maiello, Alexander G. White, Caylin G. Winchell, Sarah K. Nyquist, Sharie Keanne C. Ganchua, Amy Myers, Kush V. Patel, Cassaundra L. Ameel, Catherine T. Cochran, Samira Ibrahim, Jaime A. Tomko, Lonnie James Frye, Jacob M. Rosenberg, Angela Shih, Michael Chao, Edwin Klein, Charles A. Scanga, Jose Ordovas-Montanes, Bonnie Berger, Joshua T. Mattila, Rajhmun Madansein, J. Christopher Love, Philana Ling Lin, Alasdair Leslie, Samuel M. Behar, Bryan Bryson, JoAnne L. Flynn, Sarah M. Fortune, and Alex K. Shalek

Fig S1

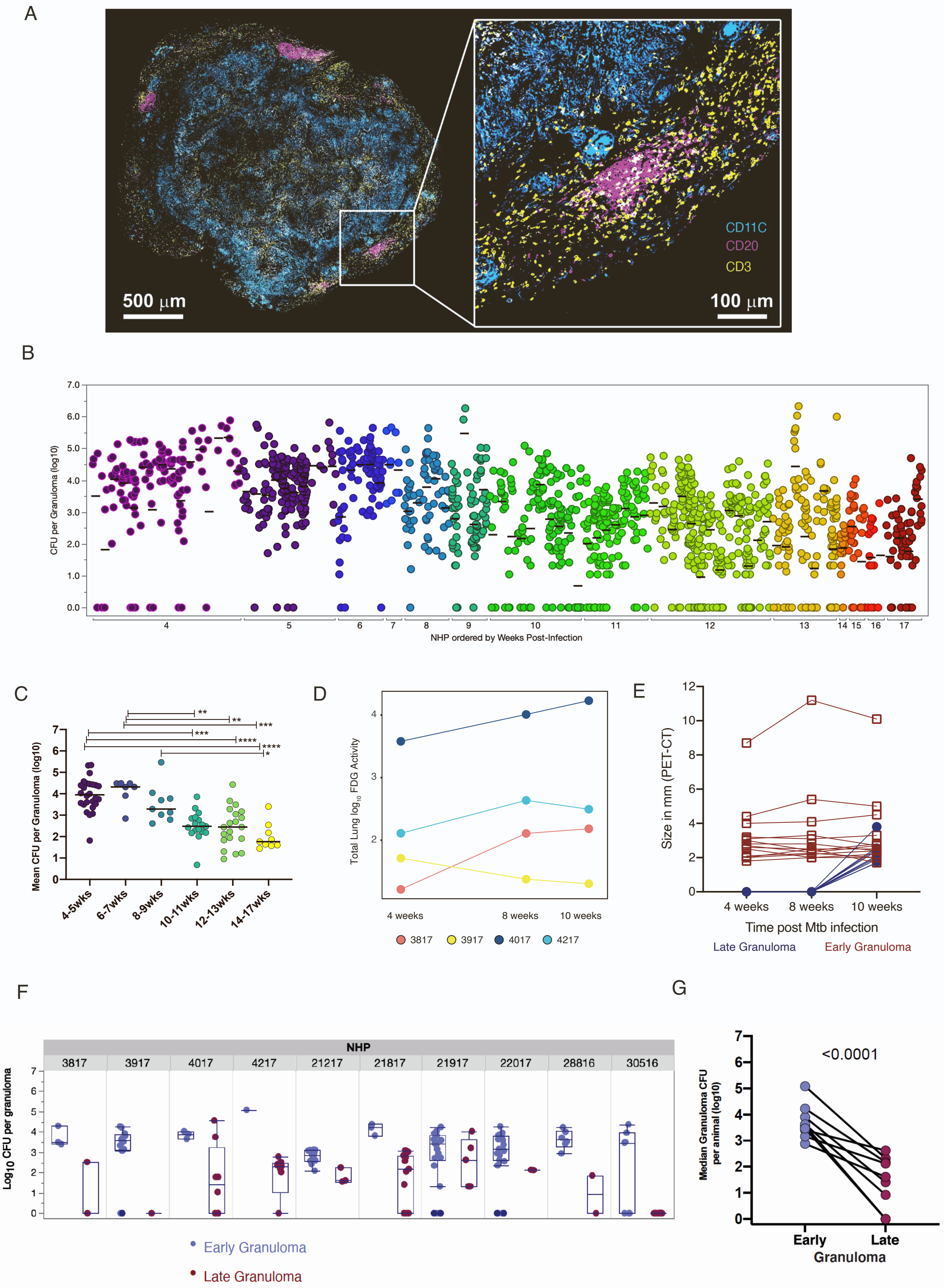

A
500 µm
100 µm
CD11C
CD20
CD3
B
CFU per Granuloma (log10)
NHP ordered by Weeks Post-Infection
4
5
6
7
8
9
10
11
12
13
14
15
16
17
C
Mean CFU per Granuloma (log10)
4-5wks
6-7wks
8-9wks
10-11wks
12-13wks
14-17wks
**
***
****
*
D
Total Lung log_{10} FDG Activity
4 weeks
8 weeks
10 weeks
3817
3917
4017
4217
E
Size in mm (PET-CT)
4 weeks
8 weeks
10 weeks
Time post Mtb infection
Late Granuloma
Early Granuloma
F
NHP
3817
3917
4017
4217
21217
21817
21917
22017
28816
30516
Log_{10} CFU per granuloma
Early Granuloma
Late Granuloma
G
Median Granuloma CFU per animal (log10)
<0.0001
Early
Late
Granuloma

Figure S1. Characterization of granuloma architecture and bacterial burden dynamics (related to Fig 1)

(**A**) Architecture of macaque TB lung granuloma, where lymphocytes and macrophages are present in distinct regions. Immunohistochemistry and confocal microscopy were performed on a granuloma from an animal at 11 weeks post-Mtb infection to visualize localization of CD11c+ macrophages (cyan), CD3+ T cells (yellow), and CD20+ B cells (magenta). (**B**) Each column depicts the CFU for all granulomas of an individual macaque (N=88 macaques), ranging from 4 weeks to 17 weeks post-infection. Each dot represents a granuloma. Lines are at means (per animal) and different colors represent weeks post-infection. (**C**) CFU per granuloma decreases significantly starting at 10-11 weeks post-infection. Each dot represents the mean CFU per granuloma of an individual animal, with the x-axis indicating weeks post-infection at which necropsy was performed. Lines are at medians. Differences between time points were tested using Kruskal-Wallis test with Dunn's multiple comparison adjustment. (* $p < 0.05$, ** $p < 0.01$, *** $p < 0.001$, **** $p < 0.0001$.) (**D**) Total lung FDG activity (in log scale) measured by PET scans of each animal at 4, 8 and 10-weeks post-Mtb infection showing trajectories of lung inflammation. (**E**) Size of each granuloma measured by CT scans at 4, 8 and 10 weeks post-mtb infection. Early granulomas are those identified at 4 weeks post infection (in maroon) and late granulomas are those identified at 10 weeks post infection (in dark blue). (**F**) CFU per granuloma is shown for early detection (blue) and late detection (red) within each animal. Box plots lines represent the median, IQR and range Each dot represents a granuloma. (**G**) CFU is significantly lower in new granulomas within animals. Each dot (and line) represents the median CFU per granuloma of each animal. Statistics: paired t-test

Fig S2

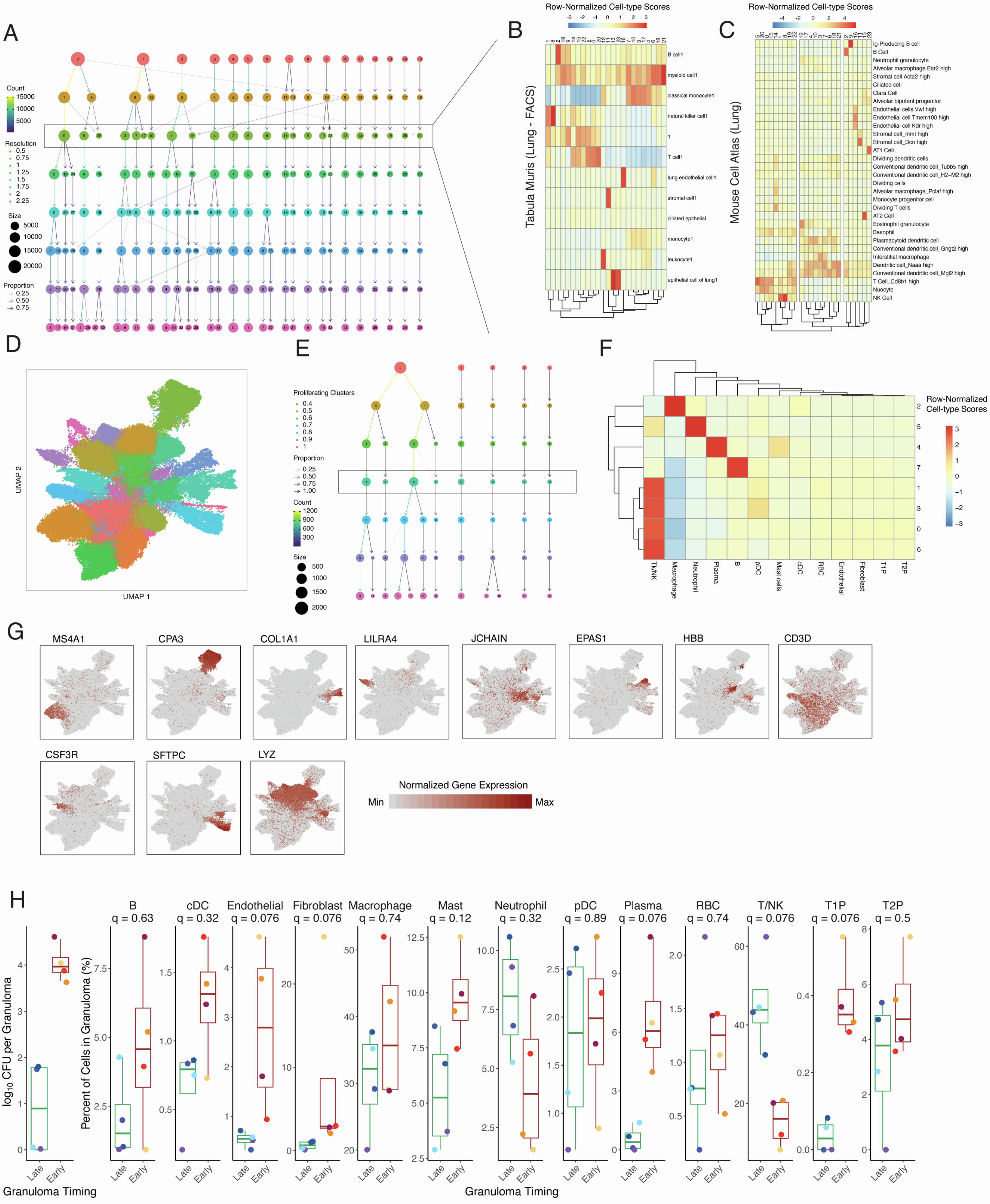

A
B
C
D
E
F
G
H
Row-Normalized Cell-type Scores
Tabula Muris (Lung - FACS)
Mouse Cell Atlas (Lung)
Count
Resolution
Size
Proportion
Proliferating Clusters
UMAP 1
UMAP 2
Row-Normalized Cell-type Scores
MS4A1
CPA3
COL1A1
LILRA4
JCHAIN
EPAS1
HBB
CD3D
CSF3R
SFTPC
LYZ
Normalized Gene Expression
Min
Max
log10 CFU per Granuloma
Percent of Cells in Granuloma (%)
Granuloma Timing
B q = 0.63
cDC q = 0.32
Endothelial q = 0.076
Fibroblast q = 0.076
Macrophage q = 0.74
Mast q = 0.12
Neutrophil q = 0.32
pDC q = 0.89
Plasma q = 0.076
RBC q = 0.74
T/NK q = 0.076
T1P q = 0.076
T2P q = 0.5
Late
Early

Figure S2. Identification of Canonical Cell Types (related to Fig 2)

(A) Waterfall plot showing stability of cell-type clustersat multiple clustering resolutions. Boxed row (resolution=1.00) selected for downstream analysis. **(B, C)** Distribution of lung cell-type signatures obtained from the Tabula Muris (B) and Mouse cell (C) atlas. (**D**) UMAP plot of 109,584 cells colored by Louvain clusters (resolution = 1.00). **(E)** Waterfall plot showing the stability of sub-clustering analysis of 3,123 cells with a proliferating gene signature. **(F)** Distribution of canonical cell type signatures across subclusters of proliferating cells. **(G)** Expression levels of cluster-defining genes overlaid on UMAP plot in panel 2A. (**H) Left:** CFU per granuloma based on the timing of detection by PET CT scan in one animal : 4017. **Right:** Difference in granuloma proportional composition of cell type clusters between early (maroon box plot) and late granulomas (green) within an animal (4017). Each granuloma is coloured. Statistics: Mann Whitney U test with Benjamini-Hochberg multiple testing correction. Box plot showing median, IQR and range; each dot represents a granuloma.

Fig S3

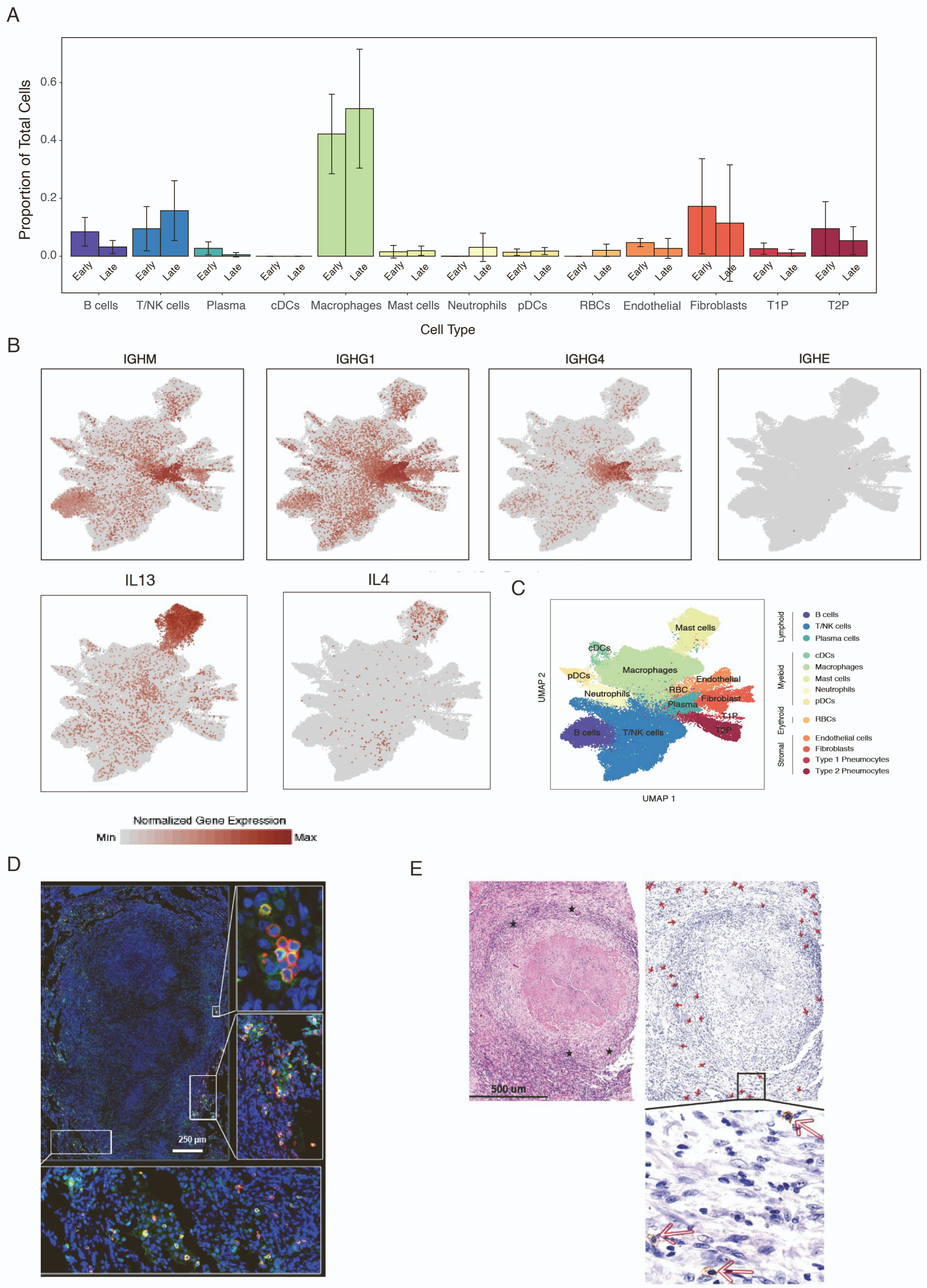

Figure S3. Cell type confirmation and Expression of selected functional transcripts (related to Fig 1-2)

(A) Proportion of cell types in granulomas from bulk sequencing of 6 early and 6 late granulomas to confirm the trend seen in scRNAseq. **(B)** UMAP plot of 109,584 cells from 26 granulomas colored by identities of 13 generic cell types. **(C)** Expression levels of select functional genes overlaid on UMAP plot of 109,584 cells. **(D)** Detection of mast cells in a 10-week NHP granuloma using immunohistochemistry, staining for tryptase (green) and c-kit (CD117)(red). **(E)** Detection of mast cells in a human lung granuloma. Hematoxylin and eosin stain and immunohistochemistry with multinucleated giant cells (stars, (top left) and c-kit (CD117) staining (indicated by arrows, top and bottom right).

Fig S4

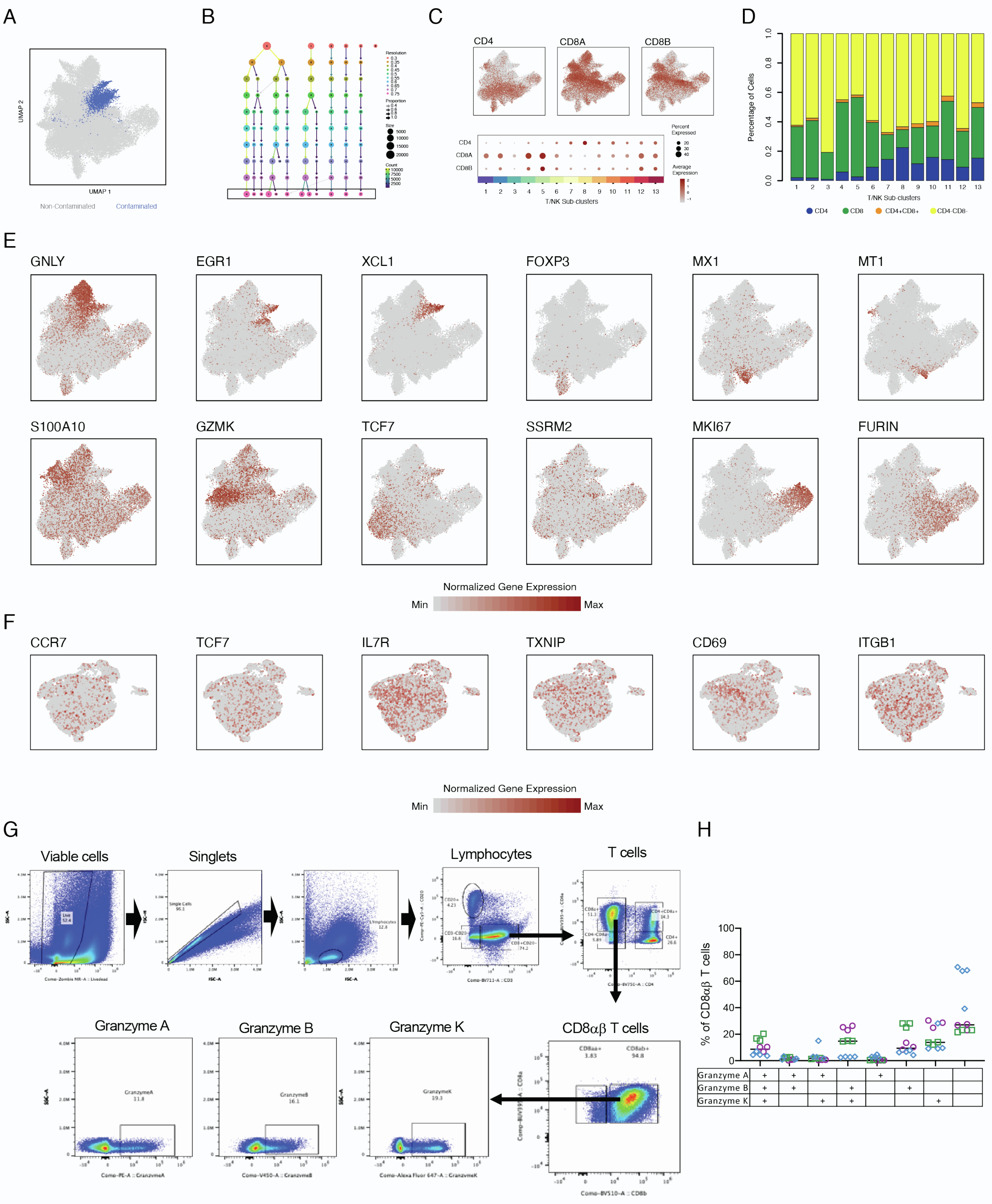

A
UMAP 2
UMAP 1
Non-Contaminated
Contaminated
B
Resolution
Proportion
Size
Count
C
CD4
CD8A
CD8B
Percent Expressed
Average Expression
T/NK Sub-clusters
D
Percentage of Cells
T/NK Sub-clusters
CD4
CD8
CD4+CD8+
CD4-CD8-
E
GNLY
EGR1
XCL1
FOXP3
MX1
MT1
S100A10
GZMK
TCF7
SSRM2
MKI67
FURIN
Normalized Gene Expression
Min
Max
F
CCR7
TCF7
IL7R
TXNIP
CD69
ITGB1
Normalized Gene Expression
Min
Max
G
Viable cells
Singlets
Lymphocytes
T cells
Granzyme A
Granzyme B
Granzyme K
CD8αβ T cells
H
% of CD8αβ T cells
Granzyme A
Granzyme B
Granzyme K

Figure S4. Sub-clustering and phenotypic identification of T/NK cell populations (related to Fig 3-4)

(A) UMAP plot of 44,766 T/NK cells with a sub-cluster of 3,544 T/NK cells defined by residual contamination highlighted (blue). **(B)** Waterfall plot showing the stability of T/NK cell sub-clustering following removal of contaminated T cell sub-cluster. Boxed row (resolution=0.75) selected for downstream analysis. **(C)** T/NK subclustering UMAP overlaid with normalized gene expression for CD4, CD8A, and CD8B (top). Expression of these genes across 13 sub-clusters (bottom) where color intensity corresponds to level of gene expression and size of dots represents the percent of cells with non-zero expression in each cluster. **(D)** Frequency of expression of *CD4* (blue), *CD8A* and/ *CD8B* (green), *CD4* and *CD8A/B* (orange) or no expression of *CD4/CD8A/B* (yellow) across 13 T/NK cell subclusters. **(E)** UMAP plots overlaid with normalized expression levels for selected T/NK cell subcluster-defining genes. **(F)** Expression of key T cell markers on subclustered stem-like T cell population. **(G)** Gating Tree showing identification CD8ab T cells in lung granuloma samples and population of Granzyme A, Granzyme B and Granzyme K + CD8abT cells. **(H)** Frequency of CD8ab T cells in lung granulomas making one or more (two , three) types of Granzymes (A, B or K). Each symbol is a granuloma and each colour identifies an animal. This data supports different types of granzyme producing cytotoxic cells identified in scRNAseq.

Fig S5

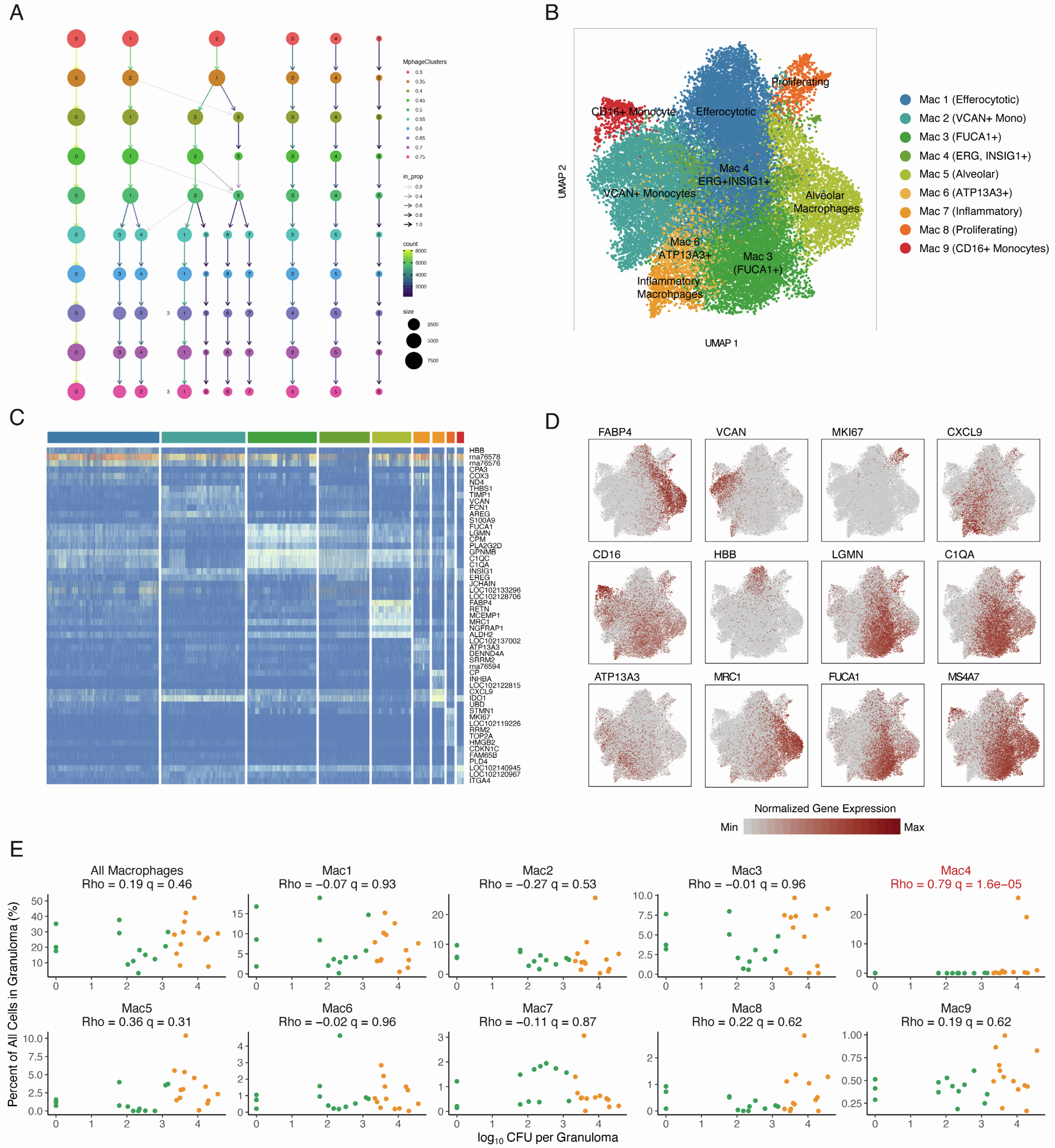

Figure S5. Macrophage heterogeneity in Mtb granulomas (related to Fig 2)

(A) Waterfall plot showing the stability of macrophage sub-clusters. Boxed row (resolution=0.55) selected for downstream analysis. **(B)** UMAP plot of 27,670 macrophage cluster colored by phenotypes. **(C)** Cluster-defining genes across macrophage subclusters. **(D)** Macrophage subcluster-defining genes overlaid on macrophage plot in panel B. **(E)** Significant correlations between proportion of Macrophage subclusters with bacterial burden of individual granulomas (Log_{10} CFU per granuloma) using non-parametric Spearman's rho correlation test. Color indicated binned granuloma bacterial burden: low (green) and high (orange).

Fig S6

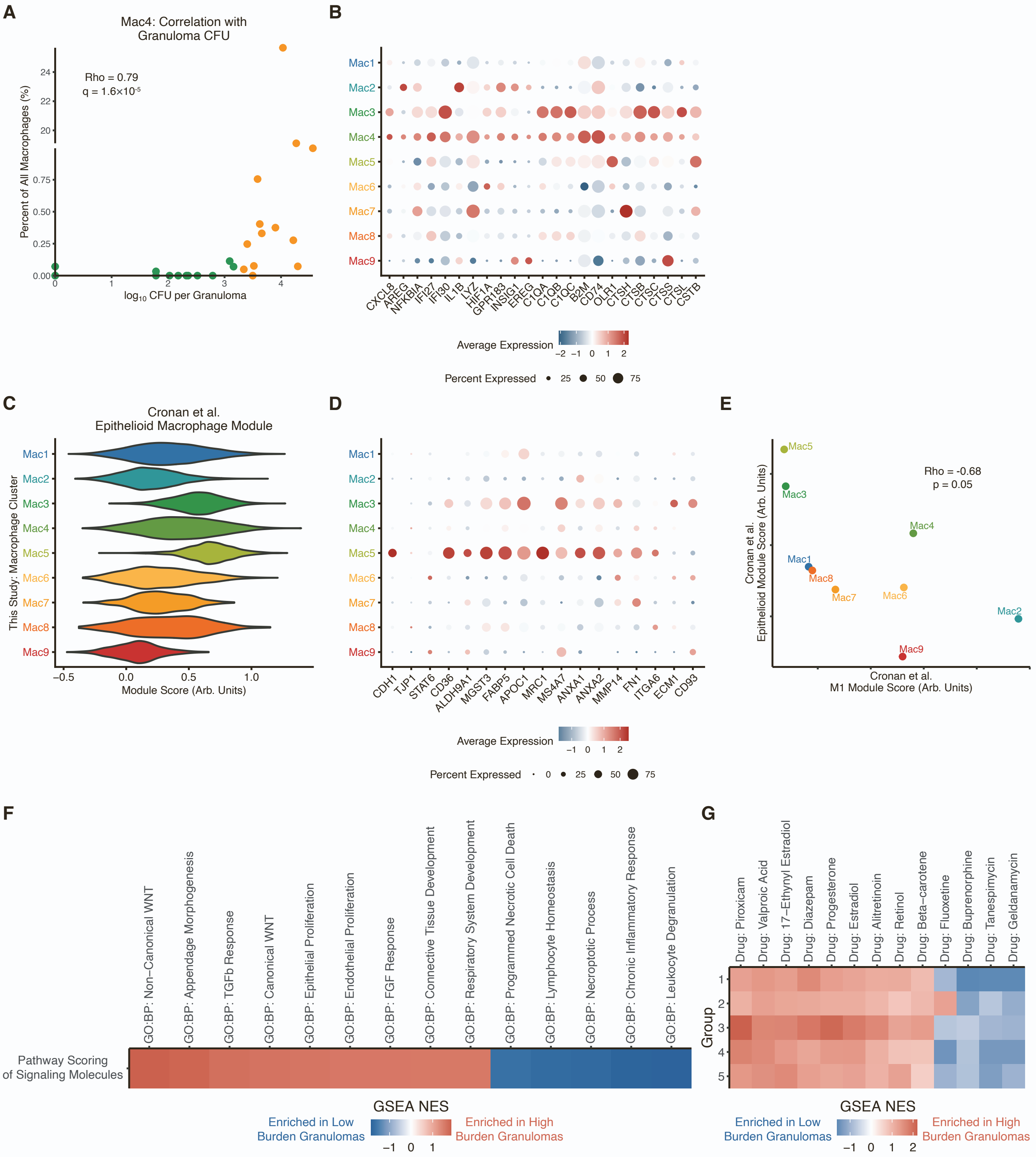
A
Mac4: Correlation with
Granuloma CFU
Rho = 0.79
q = 1.6×10-5
Percent of All Macrophages (%)
log10 CFU per Granuloma
B
Mac1
Mac2
Mac3
Mac4
Mac5
Mac6
Mac7
Mac8
Mac9
CXCL8 AREG NFKBIA IFI27 IFI30 IL1B LYZ HIF1A GPR183 INSIG1 EREG C1QA C1QB C1QC B2M CD74 OLR1 CTSH CTSB CTSC CTSS CTSL CSTB
Average Expression
Percent Expressed
C
Cronan et al.
Epithelioid Macrophage Module
This Study: Macrophage Cluster
Module Score (Arb. Units)
D
CDH1 TJP1 STAT6 CD36 ALDH9A1 MGST3 FABP5 APOC1 MRC1 MS4A7 ANXA1 ANXA2 MMP14 FN1 ITGA6 ECM1 CD93
Average Expression
Percent Expressed
E
Rho = -0.68
p = 0.05
Cronan et al.
Epithelioid Module Score (Arb. Units)
Cronan et al.
M1 Module Score (Arb. Units)
F
GO:BP: Non−Canonical WNT
GO:BP: Appendage Morphogenesis
GO:BP: TGFb Response
GO:BP: Canonical WNT
GO:BP: Epithelial Proliferation
GO:BP: Endothelial Proliferation
GO:BP: FGF Response
GO:BP: Connective Tissue Development
GO:BP: Respiratory System Development
GO:BP: Programmed Necrotic Cell Death
GO:BP: Lymphocyte Homeostasis
GO:BP: Necroptotic Process
GO:BP: Chronic Inflammatory Response
GO:BP: Leukocyte Degranulation
Pathway Scoring
of Signaling Molecules
GSEA NES
Enriched in Low
Burden Granulomas
Enriched in High
Burden Granulomas
G
Drug: Piroxicam
Drug: Valproic Acid
Drug: 17−Ethynyl Estradiol
Drug: Diazepam
Drug: Progesterone
Drug: Estradiol
Drug: Alitretinoin
Drug: Retinol
Drug: Beta−carotene
Drug: Fluoxetine
Drug: Buprenorphine
Drug: Tanespimycin
Drug: Geldanamycin
Group
GSEA NES
Enriched in Low
Burden Granulomas
Enriched in High
Burden Granulomas

Figure S6. Connecting cell states and interactions to prior knowledge (related to Fig 2, 6)

(A) Correlation between Mac4 compositional abundance and granuloma burden, using Spearman's rho correlation test with Benjamini-Hochberg correction for multiple hypothesis testing. **(B)** Expression levels marker genes that align the Mac4 subcluster with pro-inflammatory, interferon-responsive macrophages, as in Esaulova et al. Color intensity corresponds to level of gene expression, and size of dots represents the proportion of cells with non-zero expression in each cluster. **(C)** Module scoring of macrophage clusters from this study against markers associated with Cronan et al.'s epithelioid macrophage populations. **(D)** Expression levels marker genes that align the Mac3 and Mac5 subclusters with epithelioid, pro-remodeling macrophage phenotypes, as in Cronan et al. Color intensity corresponds to level of gene expression, and size of dots represents the proportion of cells with non-zero expression in each cluster. **(E)** Module scoring of macrophage clusters from this study against M1 and epithelioid gene modules from Cronan et al; Spearman's rho correlation test. **(F)** Pathways enriched in signaling molecules associated with high vs. low granuloma burden. Signaling molecules were ranked according to their log(fold-change in high vs. low burden granulomas) as input to GSEA. **(G)** Drugs with targets enriched in signaling molecules associated with each cell type group.
