# Supplementary material for: Multimodal profiling of lung granulomas in macaques reveals cellular correlates of tuberculosis control
Source: Immunity. 2022 May 10;55(5):827–846.e10. doi: 10.1016/j.immuni.2022.04.004 (PMC9122264; doi:10.1016/j.immuni.2022.04.004)
Supplement: Document S2. Article plus supplemental information [file mmc7.pdf]

Article

Immunity

Multimodal profiling of lung granulomas in macaques reveals cellular correlates of tuberculosis control

Graphical abstract

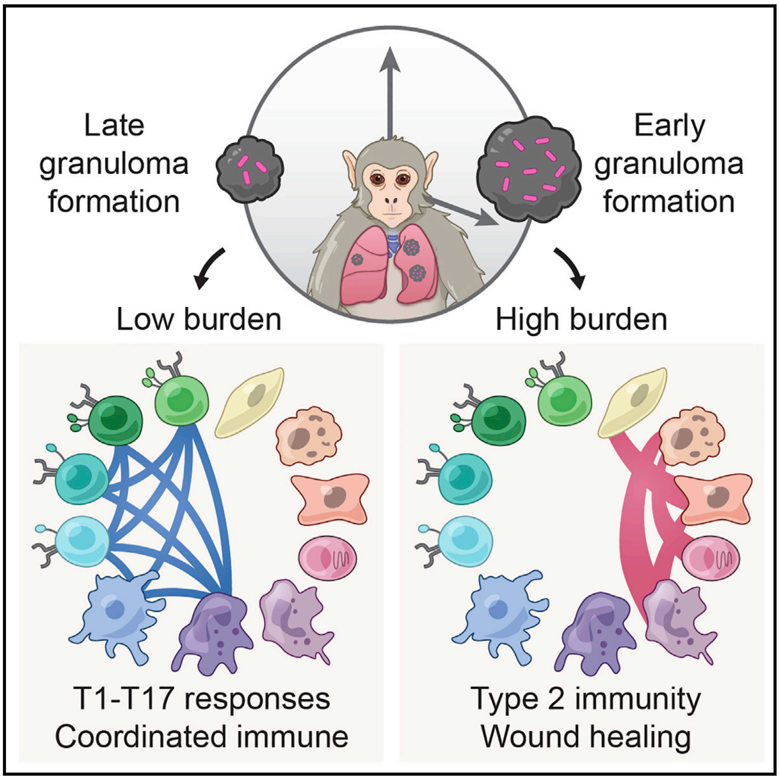

Highlights

- Timing of granuloma formation influences local microenvironment and bacterial burden
- Mast cells, type 2 immunity, and tissue remodeling underlie early, high-burden granulomas
- Type1-type17 and cytotoxic T cells associate with late-forming, low-burden granulomas
- Distinct interaction circuits across granuloma phenotypes nominate therapeutic targets

Authors

Hannah P. Gideon, Travis K. Hughes, Constantine N. Tzouanas, ..., JoAnne L. Flynn, Sarah M. Fortune, Alex K. Shalek

Correspondence

joanne@pitt.edu (J.L.F.), sfortune@hsph.harvard.edu (S.M.F.), shalek@mit.edu (A.K.S.)

In brief

Tuberculosis granulomas exhibit independent trajectories even within a single individual: some granulomas permit high bacterial growth, whereas others successfully resolve. Gideon et al. leveraged longitudinal PET-CT, bacterial burden assays, and scRNA-seq in cynomolgus macaques to define dynamic cellular phenotypes and circuits underlying granuloma persistence and immune control. Uncovered cell states and interactions may represent targets for host-directed therapies and vaccines.

Gideon et al., 2022, Immunity *55*, 827–846
May 10, 2022 © 2022 The Author(s). Published by Elsevier Inc.
https://doi.org/10.1016/j.immuni.2022.04.004

CellPress

Immunity

CellPress
OPEN ACCESS

Article

Multimodal profiling of lung granulomas in macaques reveals cellular correlates of tuberculosis control

Hannah P. Gideon,[1,2,23] Travis K. Hughes,[3,4,5,23] Constantine N. Tzouanas,[3,4,5,23] Marc H. Wadsworth II,[3,4,5,6] Ang Andy Tu,[7] Todd M. Gierahn,[7] Joshua M. Peters,[4,7] Forrest F. Hopkins,[4,8] Jun-Rong Wei,[4,8] Conner Kummerlowe,[9] Nicole L. Grant,[1] Kievershen Nargan,[10] Jia Yao Phuah,[1] H. Jacob Borish,[1] Pauline Maiello,[1] Alexander G. White,[1] Caylin G. Winchell,[1,2,11] Sarah K. Nyquist,[3,4,5,9,12] Sharie Keanne C. Ganchua,[1] Amy Myers,[1] Kush V. Patel,[1] Cassaundra L. Ameel,[1] Catherine T. Cochran,[1] Samira Ibrahim,[3,4,5] Jaime A. Tomko,[1] Lonnie James Frye,[1] Jacob M. Rosenberg,[4,8,13] Angela Shih,[13] Michael Chao,[4,8] Edwin Klein,[14] Charles A. Scanga,[1,2] Jose Ordovas-Montanes,[4,5] Bonnie Berger,[12] Joshua T. Mattila,[2,15] Rajhmun Madansein,[16] J. Christopher Love,[4,17,18] Philana Ling Lin,[2,19] Alasdair Leslie,[10,20,21] Samuel M. Behar,[22] Bryan Bryson,[4,7] JoAnne L. Flynn,[1,2,24,*] Sarah M. Fortune,[4,5,8,24,*] and Alex K. Shalek[3,4,5,6,18,24,25,*]
[1]Department of Microbiology and Molecular Genetics, University of Pittsburgh School of Medicine, Pittsburgh, PA, USA
[2]Center for Vaccine Research, University of Pittsburgh, Pittsburgh, PA, USA
[3]Institute for Medical Engineering & Science, Massachusetts Institute of Technology, Cambridge, MA, USA
[4]Ragon Institute of MGH, MIT, and Harvard, Cambridge, MA, USA
[5]Broad Institute of MIT and Harvard, Cambridge, MA, USA
[6]Department of Chemistry, Massachusetts Institute of Technology, Cambridge, MA, USA
[7]Department of Biological Engineering, Massachusetts Institute of Technology, Cambridge, MA, USA
[8]Department of Immunology and Infectious Diseases, Harvard T.H. Chan School of Public Health, Boston, MA, USA
[9]Program in Computational and Systems Biology, Massachusetts Institute of Technology, Cambridge, MA, USA
[10]Africa Health Research Institute, Durban, South Africa
[11]Division of Pulmonary, Allergy and Critical Care Medicine, University of Pittsburgh School of Medicine, Pittsburgh, PA, USA
[12]Computer Science and Artificial Intelligence Laboratory, Massachusetts Institute of Technology, Cambridge, MA, USA
[13]Division of Infectious Diseases, Massachusetts General Hospital, Boston, MA, USA
[14]Division of Laboratory Animal Research, University of Pittsburgh, Pittsburgh PA, USA
[15]Department of Infectious Diseases and Microbiology, Graduate School of Public Health, University of Pittsburgh, Pittsburgh, PA, USA
[16]Department of Cardiothoracic Surgery, University of KwaZulu Natal, Durban, South Africa
[17]Department of Chemical Engineering, Massachusetts Institute of Technology, Cambridge, MA, USA
[18]The Koch Institute for Integrative Cancer Research, Massachusetts Institute of Technology, Cambridge, MA, USA
[19]Department of Pediatrics, University of Pittsburgh School of Medicine, UPMC Children's Hospital of Pittsburgh, Pittsburgh, PA, USA
[20]School of Laboratory Medicine and Medical Sciences, University of KwaZulu-Natal, Durban, South Africa
[21]Department of Infection and Immunity, University College London, London, UK
[22]Department of Microbiology and Physiological Systems, University of Massachusetts Medical School, Worcester, MA, USA
[23]These authors contributed equally
[24]These authors contributed equally
[25]Lead contact
*Correspondence: joanne@pitt.edu (J.L.F.), sfortune@hsph.harvard.edu (S.M.F.), shalek@mit.edu (A.K.S.)
https://doi.org/10.1016/j.immuni.2022.04.004

SUMMARY

Mycobacterium tuberculosis lung infection results in a complex multicellular structure: the granuloma. In some granulomas, immune activity promotes bacterial clearance, but in others, bacteria persist and grow. We identified correlates of bacterial control in cynomolgus macaque lung granulomas by co-registering longitudinal positron emission tomography and computed tomography imaging, single-cell RNA sequencing, and measures of bacterial clearance. Bacterial persistence occurred in granulomas enriched for mast, endothelial, fibroblast, and plasma cells, signaling amongst themselves via type 2 immunity and wound-healing pathways. Granulomas that drove bacterial control were characterized by cellular ecosystems enriched for type 1-type 17, stem-like, and cytotoxic T cells engaged in pro-inflammatory signaling networks involving diverse cell populations. Granulomas that arose later in infection displayed functional characteristics of restrictive granulomas and were more capable of killing Mtb. Our results define the complex multicellular ecosystems underlying (lack of) granuloma resolution and highlight host immune targets that can be leveraged to develop new vaccine and therapeutic strategies for TB.

This is an open access article under the CC BY license (http://creativecommons.org/licenses/by/4.0/).

INTRODUCTION

Tuberculosis (TB), caused by *Mycobacterium tuberculosis* (Mtb), remains a major global health threat (WHO, 2019). Mtb infection is characterized by the formation of granulomas predominantly in the lungs and lymph nodes (Flynn and Klein, 2011; Lin et al., 2014b; Russell et al., 2010; Ulrichs and Kaufmann, 2006). These spatially organized structures, composed of a mixture of immune and non-immune cells (Ehlers and Schaible, 2013; Flynn and Klein, 2011; Gideon et al., 2019; Lin et al., 2006; Mattila et al., 2013; Pagan and Ramakrishnan, 2014; Phuah et al., 2012; Reece and Kaufmann, 2012; Ulrichs and Kaufmann, 2006), are key sites of host-pathogen interactions that can either restrict or facilitate bacterial survival. Delineating protective responses in humans has been challenging given the limited accessibility of affected lung tissue and difficulty determining the true extent of bacterial control. The cynomolgus macaque model of Mtb infection recapitulates the diversity of human outcomes and granuloma pathologies and enables detailed studies of the features of immunologic success and failure in Mtb granulomas (Canetti, 1955; Flynn and Klein, 2011; Lin et al., 2006).

A spectrum of granuloma types, organization, and cellular composition has been described in both humans and non-human primates (NHPs) (Canetti, 1955; Flynn and Klein, 2011; Hunter, 2011, 2016; Lin et al., 2006). The bacterial burden in individual granulomas is highest early in infection and then decreases due to increased bacterial killing as the immune response matures, even in macaques that ultimately develop active TB (Cadena et al., 2016; Lin et al., 2014b; Maiello et al., 2018). Strikingly, however, individual granulomas within a single host follow independent trajectories with respect to inflammation, cellular composition, reactivation risk, and ability to kill Mtb (Coleman et al., 2014b; Gideon et al., 2015; Lenaerts et al., 2015; Lin et al., 2013, 2014b; Malherbe et al., 2016; Martin et al., 2017). We and others have profiled immune responses among individual cell types in macaque lung granulomas, including those of T cells (Diedrich et al., 2020; Foreman et al., 2016; Gideon et al., 2015; Lin et al., 2012; Mattila et al., 2011; Wong et al., 2018), macrophages (Mattila et al., 2013), B cells (Phuah et al., 2012, 2016), and neutrophils (Gideon et al., 2019; Mattila et al., 2015) and have also examined the instructive roles of cytokines, including interferon (IFN)-γ, interleukin (IL)-2, tumor necrosis factor (TNF), IL-17, and IL-10 (Gideon et al., 2015; Lin et al., 2010; Wong et al., 2020). Although these analyses have led to insights into how specific canonical cell types and effector molecules relate to bacterial burden, they have not yet revealed how the integrated actions of diverse cell types within individual granulomas influence control.

High-throughput single-cell genomic profiling methods afford new opportunities to define the cell types, phenotypic states, and intercellular circuits that comprise granulomas and inform their dynamics (Prakadan et al., 2017). Here, we developed and applied a multifactorial profiling pipeline—integrating longitudinal positron emission tomography and computed tomography (PET-CT) imaging, single-cell RNA sequencing (scRNA-seq), and molecular measures of bacterial killing with immunohistochemistry and flow cytometry—to identify features of TB lung granulomas that correlate with bacterial clearance in cynomolgus macaques. We defined the cellular compositions and cell-cell signaling networks associated with bacterial persistence or control. Collectively, our data define the cellular ecosystems within TB lung granulomas in which Mtb is controlled or alternatively survives and multiplies, uncovering therapeutic and prophylactic targets for future investigation.

RESULTS

Profiling longitudinal TB granuloma dynamics, bacterial burden, and bacterial killing

We sought to define the complex cellular ecosystems of granulomas that manifest different degrees of bacterial control in NHPs. Four cynomolgus macaques were infected with a low dose of Mtb (<10 CFU; Erdman strain) and followed for 10 weeks (Figure 1A). Ten weeks post-infection (p.i.) was chosen as a pivotal time point at which bacterial killing could be identified in some but not all granulomas during the course of immune activation and mobilization, even in macaques that would eventually progress to active TB (Figures S1A–S1C). Progression of Mtb infection and individual granuloma dynamics were monitored at 4, 8, and 10 weeks p.i. by using PET-CT imaging of FDG avidity as a proxy for inflammation (Figures S1D and S1E; Table S1) (Coleman et al., 2014b; White et al., 2017). At necropsy, individual PET-CT identified lung granulomas were excised and dissociated to obtain a single-cell suspension; viable bacterial burden (CFU, colony forming units—i.e., culturable live bacterial burden) and cumulative (live + dead) bacterial load (chromosomal equivalents, CEQ) were measured to define the extent of bacterial growth and killing in each granuloma (Lin et al., 2014b; Munoz-Elias et al., 2005).

Twenty-six granulomas from four animals were randomly selected at the time of necropsy 10 weeks p.i. for scRNA-seq analysis. Among them, there was a range of granuloma-level bacterial burdens, from sterile (0 CFU/granuloma) to high (4.6 $\log_{10}$ CFU/granuloma) (Figures 1B and 1C; Table S1). We binned the granulomas by bacterial burden (low, n = 13; high, n = 13). There was a significant difference in CFU between low and high CFU granulomas (median 2.2 [low] vs 3.6 [high] $\log_{10}$ CFU/granuloma, $p < 0.0001$, Mann-Whitney U [MWU] test) (Figure 1C). To determine whether low CFU reflected reduced bacterial growth or increased bacterial killing, we assessed the total number of bacterial genomes (CEQ), because we have previously shown that the genomes of dead bacteria are not readily cleared and that CEQ provides a measure of cumulative bacterial load (Munoz-Elias et al., 2005). There was not a significant difference in CEQ values between low- and high-burden granulomas, although there was a trend toward higher CEQ in high-burden lesions (Figure 1D). However, the extent of bacterial killing, calculated as the ratio of CFU to CEQ, was significantly higher in the low-bacterial-burden granulomas ($p = 0.03$, MWU test) (Figure 1E), indicating that the lower CFU largely reflected greater killing rather than more limited bacterial growth.

We then sought to identify granuloma features correlated with the degree of bacterial control. *Post hoc* analysis of serial PET-CT imaging data revealed a strong association between the apparent timing of lesion formation and the extent of bacterial control. All high-bacterial-burden granulomas were detected at the four-week scan, whereas most (11/13) low-bacterial-burden granulomas were first detected at the final pre-necropsy scan (10 weeks) (Figures 1F, 1G, and S1E). Consistent with these

CellPress
OPEN ACCESS

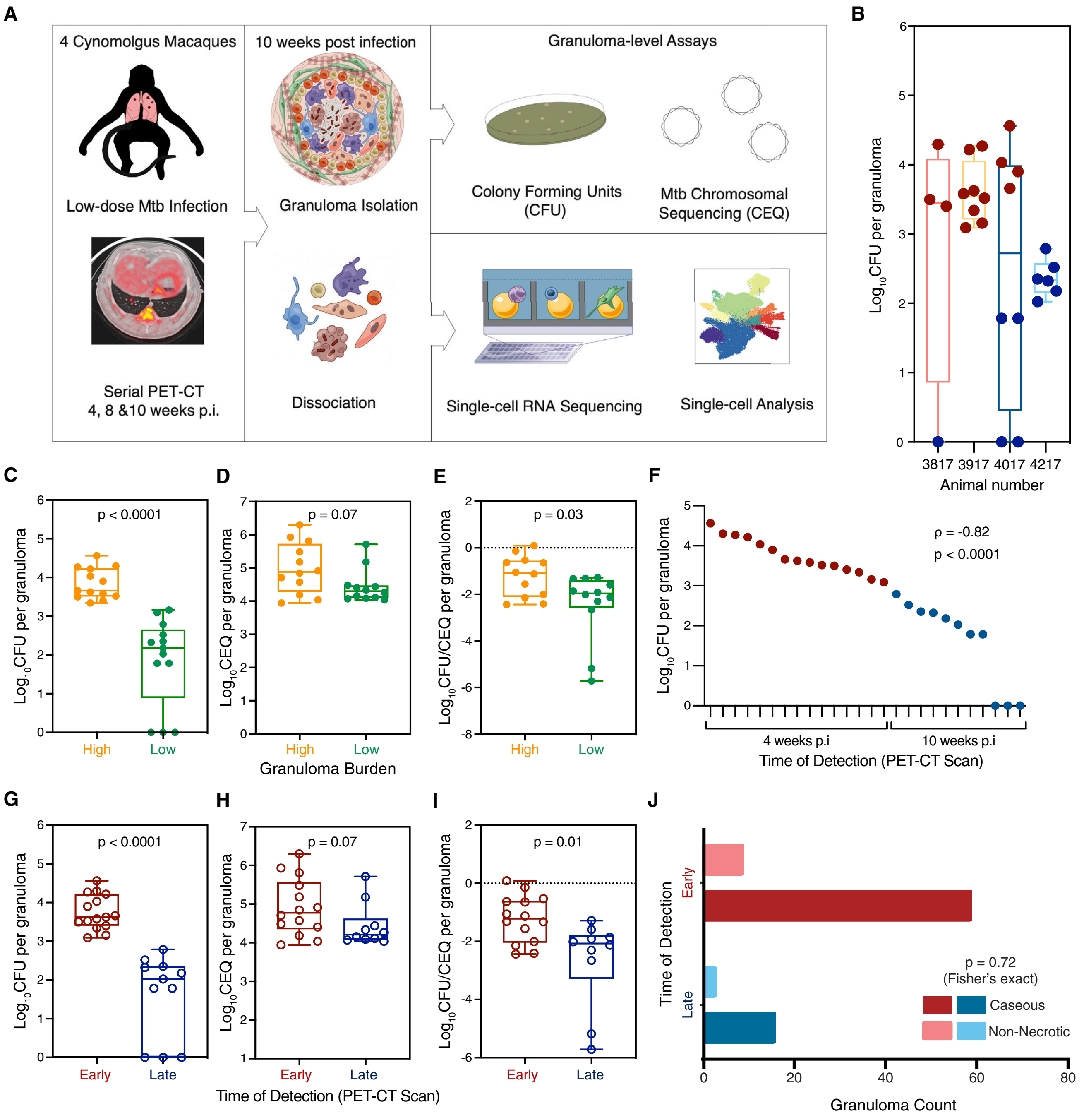

Figure 1. Characteristics of animals over the course of Mtb infection and granuloma bacterial burden

(A) Study design: cynomolgus macaques (n = 4) were infected with a low-dose inoculum of Mtb (Erdman strain), and serial PET-CT scans were performed at four, eight, and 10 weeks post-infection (p.i.), with the final scan used as a map for lesion identification at necropsy.

(B) Distribution of CFU per granuloma sampled for Seq-Well assay for each animal.

(C and G) CFU $\log_{10}$ per granuloma (total live bacteria). Box plot showing median, interquartile range, and range with MWU.

(D and H) CEQ $\log_{10}$ per granuloma (live + dead Mtb) organized by time of detection. Box plot showing median, interquartile range, and range with MWU.

(E and I) Ratio between CFU (viable bacteria) and CEQ (total bacterial burden)—i.e., relative bacterial survival. Box plot showing median, interquartile range, and range with MWU. Lower ratio (negative values) corresponds to increased killing, and higher ratio corresponds to increased Mtb survival.

(C–E) Organized by bacterial burden: low, green; high, orange.

(F) Individual granuloma bacterial burden ($\log_{10}$ CFU) plotted with time of detection by PET-CT scans: four weeks p.i. (early) or 10 weeks p.i. (late).

(F–I) Time of detection by PET-CT scan (Table S1): early granulomas (maroon), late granulomas (blue).

(J) Histological evaluation of necrosis across early-arising and late-arising granulomas at 10–12 weeks post-infection (n = 87 granulomas across 16 macaques).

See also Figures S1, S3, and S6; Table S1.

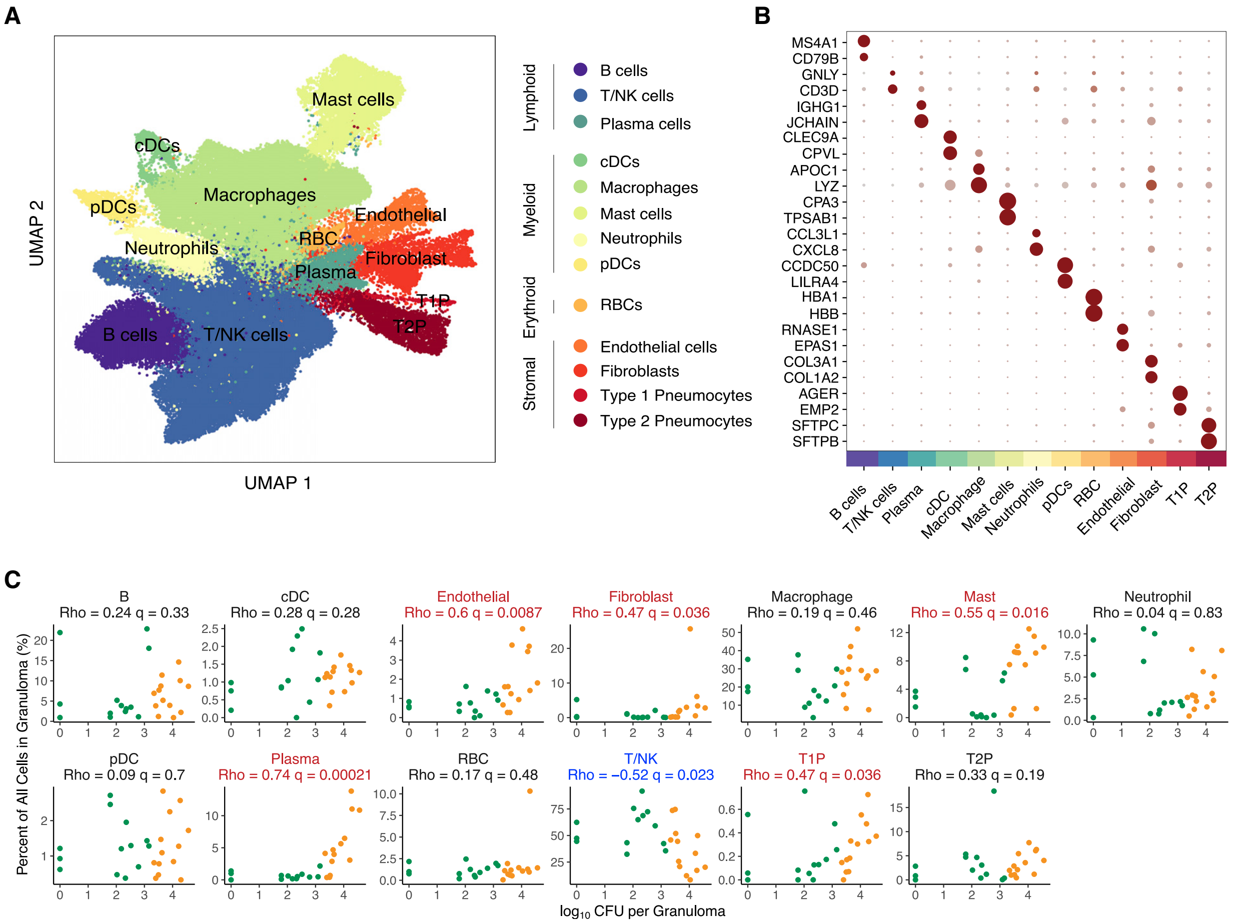

Figure 2. Analysis of scRNA-seq of tuberculosis lung granulomas

(A) Uniform manifold approximation and projection (UMAP) plot of 109,584 cells from 26 granulomas colored by identities of 13 generic cell types.

(B) Expression levels of cluster-defining genes. Color intensity corresponds to the level of gene expression, whereas the size of dots represents the percent of cells with non-zero expression in each cluster.

(C) Significant correlations between proportion of canonical cell types with bacterial burden of individual granulomas ($\log_{10}$ CFU per granuloma) using non-parametric Spearman's rho correlation test with Benjamini-Hochberg multiple testing correction. Color indicates binned granuloma bacterial burden.

See also Figures S2, S3, and S5; Table S2.

data, we further evaluated bacterial burden between early- and late-appearing granulomas in 10 additional animals at 10 weeks p.i (Figures S1F and S1G) and again found that the median CFU/granuloma per animal was significantly lower in late granulomas than in early ones ($p < 0.0001$, Student's t test). We considered the model that late lesions have lower CFU because the bacterial population had simply not had sufficient time to expand. However, the CFU/CEQ analysis was most consistent with greater bacterial killing in late-appearing granulomas (-2.1 $\log_{10}$ CFU/CEQ per granuloma) as compared to that in early-appearing ones (-1.2 $\log_{10}$ CFU/CEQ per granuloma, $p = 0.01$, MWU test) (Figure 1I).

Late-appearing granulomas could be due to differences in the timing of lesion formation, most likely due to a dissemination event from an early granuloma, such that granuloma development occurs in the context of an activated immune response, which we have previously shown to confer significant protection against reinfection (Cadena et al., 2018). Alternatively, we considered the possibility that differences in inflammatory-response characteristics, and specifically the extent of necrosis, might make some granulomas both detectable by PET-CT before others and associated with higher bacterial burdens. Therefore, we reviewed the histopathology from 87 historical granuloma samples from 16 cynomolgus macaques at 10–12 weeks p.i. but found no association between necrosis and time of granuloma detection ($p = 0.72$, Fisher exact test; Figure 1J), suggesting that bacterial control in early and late granulomas is a result of more complex factors than necrosis alone.

Cellular composition of TB lung granulomas

We next sought to identify cellular and molecular factors associated with increased Mtb killing in an unbiased fashion through scRNA-seq (STAR Methods) (Gierahn et al., 2017; Macosko et al., 2015; Young and Behjati, 2018; Lun et al., 2019; McGinnis et al., 2019; Wolf et al., 2019). Among the 10-week granulomas, we analyzed 109,584 cells, resolving 13 general cell types (Figures 2A, 2B, and S2A–S2G; Table S2; STAR Methods)

(Tabula Muris Consortium et al., 2018; Han et al., 2018; Liberzon et al., 2011; Lopez et al., 2017; Varemo et al., 2013; Guo et al., 2018; Zilionis et al., 2019). These encompass groups of lymphocytes, including B cells, T and NK cells (T/NK), and plasma cells; myeloid cells, including conventional dendritic cells (cDCs), plasmacytoid dendritic cells (pDCs), and macrophages; mast cells; neutrophils; erythroid cells; stromal cells, including endothelial cells and fibroblasts; type 1 pneumocytes; and type 2 pneumocytes (Figures 2A, 2B, and S2G; Table S2). For each of these 13 cell types, we also performed further within cell-type subclustering; in these analyses, we only detected substructure among the T/NK and macrophage clusters (detailed below, STAR Methods).

Cell types associated with timing of granuloma formation and control

To investigate the relationship between cell type composition and bacterial burden, we quantified the correlation between cellular frequency and CFU across all granulomas. Our data revealed multiple cell types that were significantly enriched in high-burden (early-appearing) granulomas, including plasma cells (relative cell abundance vs CFU, q = 0.00021, non-parametric Spearman's rho correlation test with Benjamini-Hochberg multiple testing correction), mast cells (q = 0.016), endothelial cells (q = 0.0087), and fibroblasts (q = 0.036) (Figure 2C; Table S3). By contrast, T/NK cells were more abundant in low-burden (late-appearing) granulomas (q = 0.023) (Figure 2C; Table S3). Cynomolgus macaques are variable in their infection outcomes (Figure 1B), so to control for inter-subject variability, each of the cellular associations between granuloma dynamics and bacterial control was examined both (1) across all animals and lesions and (2) through a directed analysis of the granulomas from a single NHP host (4017) (Figure S2H). We found similar trends in bulk RNA-sequencing data of 12 additional granulomas (six high-CFU [early] and six low-CFU [late] granulomas) from separate macaques (Figure S3A) (Newman et al., 2015). To account for compositional dependencies between the cell types comprising each granuloma, we also conducted a multivariate Dirichlet regression analysis, which explicitly considers how shifts in the abundance of one cell type affect the relative proportions of the others present (Smillie et al., 2019). In this framework, T/NK cells were also significantly associated with low burden (Dirichlet $p = 3.3 \times 10^{-5}$), and mast cells and plasma cells significantly associated with high burden (Dirichlet p = 0.025 & p = 0.021, respectively). We chose to prioritize cell types for further investigation based on concordance across statistical testing frameworks.

High-bacterial-burden granulomas are characterized by fibrosis and type 2 immune features

To validate our mast cell observations, we performed immunohistochemistry on NHP and human granuloma sections by using tryptase and C-kit/CD117 markers (Figures S3B–S3E) (Schindelin et al., 2012). This revealed the presence of mast cells within both NHP and human granulomas and that they primarily localize to the outer regions of NHP granulomas, including the lymphocyte cuff (Figure S3D) and could be found within and around human granulomas (Figure S3E) (Garcia-Rodriguez et al., 2017). In our data, mast cells were distinguished by their expression of *IL4* and *IL13* (Figure S3B), which we also recently observed in a study of human nasal polyposis, a type 2 inflammatory disease associated with far-reaching epithelial remodeling (Ordovas-Montanes et al., 2018). This association between mast cells and fibrosis is further supported by a study on the spatial structure of human TB granulomas, which found a class of local signaling neighborhoods characterized by elevated proportions of mast and endothelial cells and speculated about an association with tissue repair (McCaffrey et al., 2022). Mast cells were also marked by expression of *ALOX5A* and *ALOX5AP*, which encode the system to synthesize the anti-inflammatory lipoxin LXA_4; the balance between LXA_4 and the pro-inflammatory lipoxin LTB_4 has been strongly implicated in the progression of TB disease in humans (Tobin et al., 2010, 2012).

Plasma cells were also abundant in high-burden lesions, consistent with previous findings (Jacobs et al., 2016; Phuah et al., 2012). Recruitment of mast cells can be characteristic of allergic type 2 immune responses mediated by IgE (Kanagaratham et al., 2020), but mast cell function is also regulated by IgG, which is much more abundant in the circulation and tissues. Among the plasma cells in our scRNA-seq dataset, the vast majority expressed either *IGHG* or *IGHA* (Collins and Jackson, 2013) constant chains (Figures S3B and S3C), suggesting that IgG and IgA were the dominant antibody classes. Taken together, these data suggested that granulomas with failed bacterial clearance are characterized by a type 2 immune environment, but the antibody features were not consistent with a canonical allergic response.

T and NK functional subclusters as mediators of protection

Of the 13 broad cell types, only the T/NK cell subcluster was associated with more robust bacterial control in granulomas (q = 0.023; Dirichlet $p = 3.3 \times 10^{-5}$; Figure 2C). Previously, we showed that ~90% of T cells in granulomas are tissue localized, with only ~10% immigrating to the tissue from blood over a 24-h period; ~95% of the tissue-localized cells exhibit a tissue-resident memory phenotype (Potter et al., 2021). To further assess functional diversity within the T and NK cell cluster and association with bacterial burden, we performed additional sub-clustering analyses. This revealed 13 T/NK cell subclusters which we annotated based upon expression of the following: lineage-defining markers; known cytotoxic, regulatory, and proliferation genes (Figures 3A–3C and S4; Table S4); and TCR constant gene (*TRAC*, *TRBC*, and *TRDC*) expression (Figure 3B). The process of annotation revealed that most subclusters did not correspond neatly to canonical T and NK cell subsets, consistent with recent studies in other systems (Rath et al., 2020). Accordingly, we annotated each subset based on distinguishing functional patterns of gene expression by using known T cell markers and literature-derived genes of interest where possible, as opposed to ontological classification based on pre-structured developmental relationships. These genes were parts of broader transcriptional signatures that appeared to reflect dominant cellular response states superimposed on cell-lineage-associated gene-expression programs. Among the 13 T/NK cell subclusters, two were significantly negatively associated with bacterial burden (with another four trending toward significance with q<0.1) (Figure 3D; Table S3).

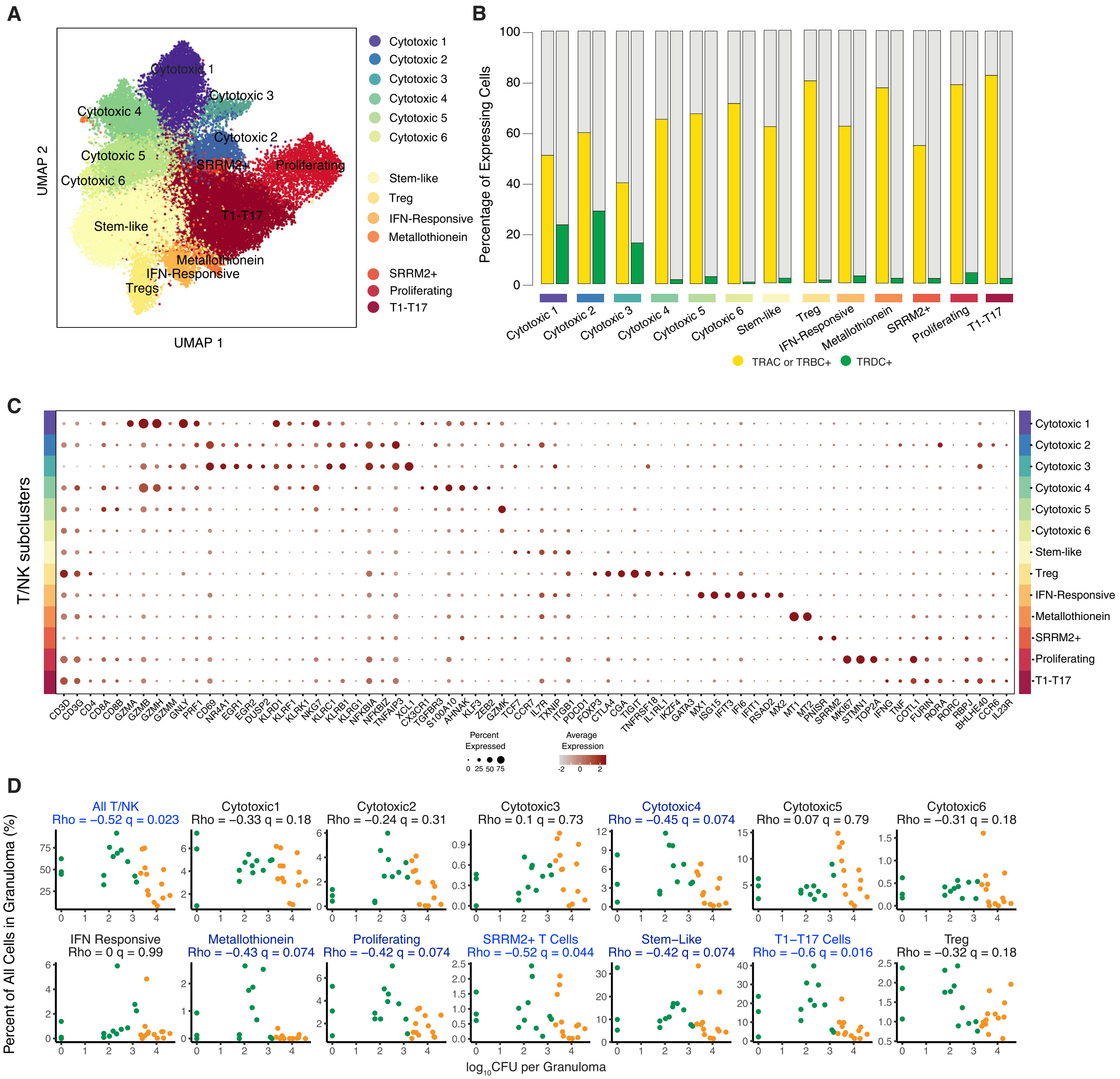

Figure 3. Diversity in the unified T and NK cell cluster and relationship to granuloma-level bacterial burden

(A) Subclustering of 41,222 cells in the unified T/NK cell cluster.

(B) Frequency of expression of TCR genes *TRAC*, *TRBC1*, or *TRBC2* (yellow) and *TRDC* (green).

(C) Expression levels of T/NK cell cluster-defining genes. Color intensity corresponds to the level of gene expression and the size of dots represents the percent of cells with non-zero expression in each cluster.

(D) Significant correlations between proportion of T/NK subclusters with bacterial burden of individual granulomas ($\log_{10}$ CFU per granuloma) using non-parametric Spearman's rho correlation test with Benjamini-Hochberg multiple testing correction.

See also Figure S4; Tables S2, S3, and S4.

A prominent role for type 1-type 17 T cells in bacterial control

One T/NK cell subcluster represented the most abundant cell type identified across all granulomas (8.8%) (Table S4), and the strongest correlate with bacterial control (q = 0.016; Dirichlet p = 3.3 × 10^{-9}) (Figure 3D; Table S3). This subcluster, which we designated type 1-type 17 (T1-T17) (Figure 3C), was enriched for expression of classical Th1-associated genes, including *IFNG* and *TNF* (Raphael et al., 2015), as well as transcription factors associated with Th17 differentiation (Yosef et al., 2013), including *RORA* (Yang et al., 2008), *RORC* (Ivanov et al., 2006), *RBPJ* (Meyer Zu Horste et al., 2016), and *BHLHE40* (Huynh

CellPress
OPEN ACCESS

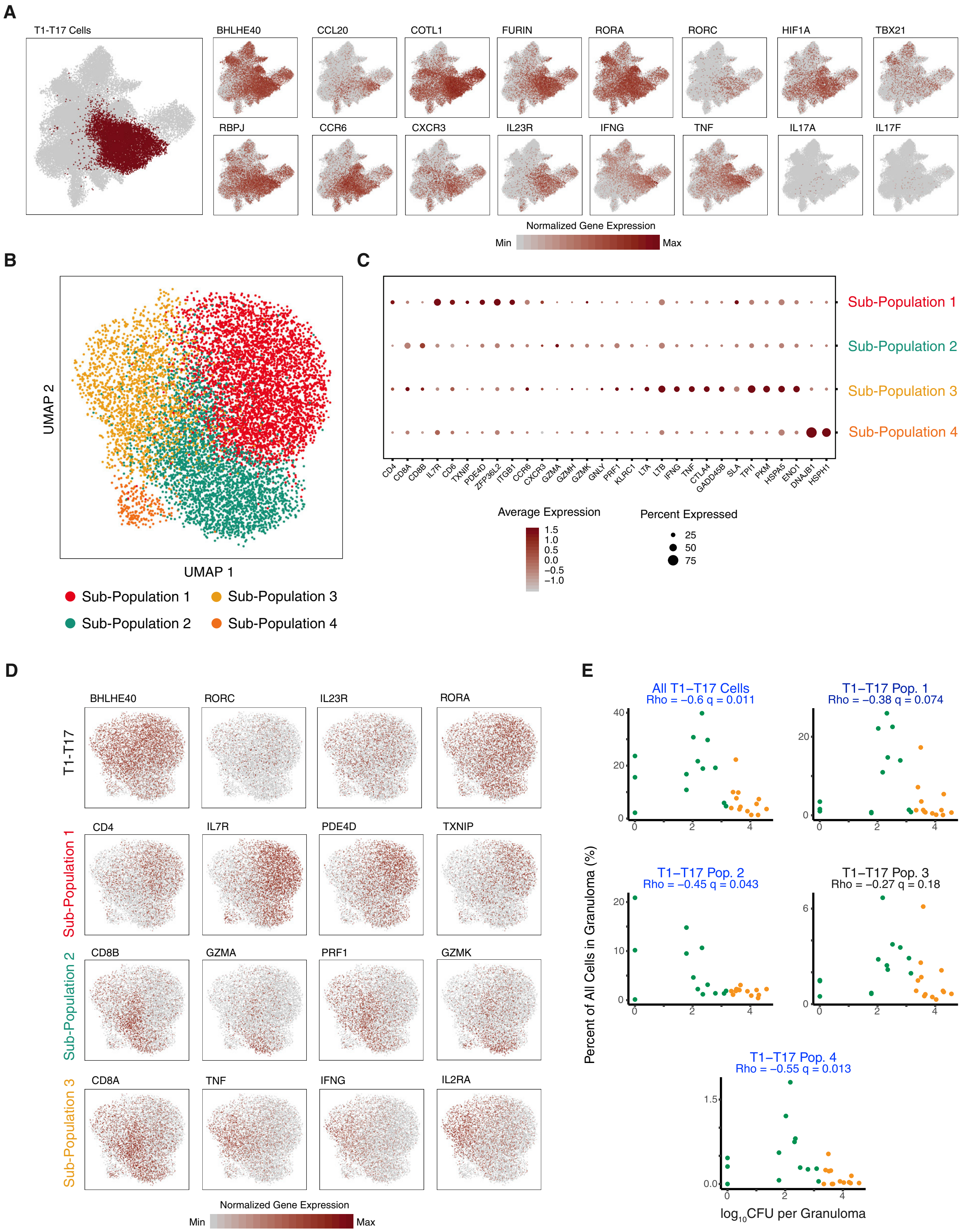

(legend on next page)

et al., 2018; Lin et al., 2014a, 2016). Although we also detected additional features of T17 cells, including *CCR6* (Hirota et al., 2007) and *IL23R* (Kobayashi et al., 2008), we did not observe expression of either *IL17A* or *IL17F* (Figure 4A; Table S4), which was consistent with our published flow-cytometry data demonstrating minimal IL-17 production from granuloma T cells (Gideon et al., 2015; Wong et al., 2020). T1-T17 cells in our dataset were double positive for *CXCR3* and *CCR6* (Figure 4A), consistent with markers for Th1* or ex-Th17 cells, which are believed to be precursors to tissue-resident memory cells. Multiple prior studies have reported a CXCR3+CCR6+ Th1/Th17 subset that contributes to Mtb-specific T cell responses (Becattini et al., 2015; Nikitina et al., 2018) and is capable of producing IFNγ, IL-17, and IL-22 after stimulation, but not Th2-biased IL-4, IL-5, or IL-13 (Acosta-Rodriguez et al., 2007; Becattini et al., 2015; Lindestam Arlehamn et al., 2013; Mahnke et al., 2013). These CXCR3+CCR6+ Th1/Th17 T cells have also been demonstrated to express Th1-associated *TBX21* (encoding T-bet) and Th17-associated *RORC* (encoding RORγt), but not Th2-associated *GATA3* (Acosta-Rodriguez et al., 2007; Becattini et al., 2015), supporting the existence of the T1-T17 cell type. Further supporting a hybrid cell state, we independently confirmed the presence of a subset of granuloma T cells expressing both T-bet and RORα by flow cytometry (Grant et al., 2022). Notably, although Th1* and ex-Th17 subsets are described primarily as CD4 T cells (Darrah et al., 2020; Gideon et al., 2015; Lyadova and Panteleev, 2015; Mpande et al., 2018), our T1-T17 sub-cluster was characterized by the expression of both *CD4* and *CD8A/B* transcripts (Figures 3C, 4A–4C, S4D, and S4E), suggesting that this phenotype is not an identity program but context dependent, consistent with findings in other systems (Lee et al., 2021).

To better resolve the identities of the cells in this cluster, we further sub-clustered the T1-T17 cells. This revealed four distinct subpopulations, each of which expressed T1-T17 cluster markers (*RORA*, *RORC*, *IL23R*, and *BHLHE40*) but were further distinguished by markers of cell type and state (Figure 4B; Table S4): T1-T17 subpopulation 1 was distinguished by expression of *CD4* and markers of activation and motility, including *IL7R*, *CD6*, *TXNIP*, *PDE4D*, *ZFP36L2*, *ITGB1*, *CCR6*, and *CXCR3* (Figures 4B–4D; Table S4), making it most akin to ex-Th17 cells (Amezcua Vesely et al., 2019; Nikitina et al., 2018); T1-T17 subpopulation 2 was characterized by increased relative expression of both *CD8A* and *CD8B* and cytotoxic effector molecules; T1-T17 subpopulation 3, which includes cells expressing either *CD8A/B* or *CD4*, was characterized by cytokine gene expression (*IFNG*, *TNF*, *LTA*, and *LTB*) and markers of an inhibitory cell state (*CTLA4*, *GADD45B*, and *SLA*); and T1-T17 subpopulation 4 was very low in abundance and characterized by heat shock and DNA damage associated transcripts (*DNAJB1* and *HSPH1*). In a univariate analysis, there was a trend toward negative association between bacterial burden and higher abundance of T1-T17 subpopulation 1 (q = 0.074) and a significant negative association between bacterial burden and abundance of T1-T17 subpopulation 2 (q = 0.043); both of these were significantly associated with low burden in the multivariate Dirichlet regression analysis (Dirichlet p = 0.001 & p = 0.018, respectively). T1-T17 subpopulation 3, however, was not correlated with bacterial burden, despite elevated expression of *IFNG* and *TNF* (Figure 4E; Table S4)—cytokines generally considered as critical mediators of control in Mtb infection (O'Garra et al., 2013; Scriba et al., 2017).

CD4 and CD8 subclusters associated with low bacterial burden

Among the remaining 12 T/NK cell subclusters, six were enriched for both *CD4* and *CD8* expression (Figures 3A–D, S4D, and S4E; Table S4). The most abundant subcluster (8.3% of granuloma cells, q = 0.074, Dirichlet p = 0.00049; Figure 3D; Tables S3 and S4) exhibited elevated expression of markers of naive and memory T cells (*TCF7*, *CCR7*, *IL7R*, and *TXNIP*) and activation or memory state (*CD69* and *ITGB1*) (Figure 3C; Table S4). When we conducted further subclustering of this population to evaluate the potential presence of separate naive or memory T cell states, we instead found that these markers were expressed homogenously and overlapped throughout the subcluster (Figure S4F). As such, these cells could represent a "stem-like" population of T cells, which has been described as an early differentiating memory phenotype, distinct from naive T cells, that are long lived and possess a distinguishing ability to proliferate and self-renew (Ahmed et al., 2016; Caccamo et al., 2018; Gattinoni et al., 2011). Further targeted experimentation will be required to establish true stem capacity for these cells in tissue.

We also identified a cluster of proliferating T CD4- and CD8-expressing cells (2.4%; q = 0.074, Dirichlet p = 0.016; Figure 3D; Tables S3 and S4), which was characterized by high expression of transcripts associated with cellular proliferation (*MKI67*, *STMN1*, and *TOP2A*) (Figure 3C; Table S4). We found a very small population of metallothionein-expressing T cells (0.05%; q = 0.074, Dirichlet p = 0.071; Figure 3D; Table S4), defined by metallothionein genes, such as *MT1* and *MT2* (Figure 3C; Table S4), which play a role in negative regulation of type 1 regulatory (Tr1) $CD4^+$ cells (Wu et al., 2013). A cluster labeled SRRM2-T cells (0.6%; q = 0.044, Dirichlet p = 0.17) was characterized by enrichment of genes associated with nuclear speckles and splicing factors such as *PNISR* and *SRRM2* (Figures 3C and 3D; Table S4).

The remaining two CD4/CD8 subclusters were not associated with bacterial control by either statistical framework. One was

Figure 4. Phenotypic Diversity in T1-T17 cells

(A) T1-T17 subcluster overlaid on unified T/NK cell cluster (left) and colored by normalized expression values for T1-T17 subcluster-defining genes (bold outlined boxes) and non-enriched canonical Type1 and type 17 genes (right).

(B) Subclustering of 9,234 T1-T17 cells resulting in four phenotypic sub-populations.

(C) Cluster-defining genes for T1-T17 subpopulations 1, 2, 3 and 4. Color intensity corresponds to the level of gene expression, and the size of dots represents the percent of cells with non-zero expression in each cluster.

(D) Subclustering of T1-T17 cells colored by normalized gene-expression values for selected subcluster (top row) and subpopulation defining genes.

(E) Significant correlations between proportion of T1-T17 subcluster and subpopulations with bacterial burden of individual granulomas (log_{10} CFU per granuloma) using non-parametric Spearman's rho correlation test with Benjamini-Hochberg multiple testing correction.

See also Figure S4; Tables S3 and S4.

CellPress
OPEN ACCESS

regulatory T cells (1.2%), defined by elevated expression of canonical Treg markers (*FOXP3*, *CTLA4*, *TIGIT*, and *IL1RL1*) and *GATA3*, a Th2 lineage-defining transcription factor that has been observed in a subset of tissue-resident Tregs (Figures 3C and 3D; Table S4) (Wohlfert et al., 2011). Of note, although *CTLA-4* was highly expressed by regulatory T cells, the inhibitory receptor PD-1 (*PDCD1*) was only sparsely detected in our data set, concordant with recent work (McCaffrey et al., 2022; Wong et al., 2018). The final subcluster was interferon-responsive T cells (0.4%), which were enriched for type-1-interferon-inducible molecules (Szabo et al., 2019) (Figures 3C and 3D, Table S4).

Bacterial control is associated with a specific cytotoxic T cell population

The remaining six T/NK subclusters were broadly defined by expression of *CD8A* and/or *CD8B* and cytotoxic genes, including granzymes, granulysin, and/or perforin (designated cytotoxic 1–6, Figure 3C; Table S4). We confirmed expression of multiple granzymes among CD8 $\alpha\beta$ T cells in Mtb granulomas by flow cytometry (Figure S8).

Low-bacterial-burden granulomas were associated with a higher proportion of cells from cytotoxic subcluster C4 (3.8% of granuloma cells; q = 0.074, Dirichlet p = 0.00042; Figure 3D; Table S4). C4 expressed both *CD8A* and *CD8B* and *TCRA* and *TCRB*, but not *TCRD*, indicating that it is composed primarily of conventional CD8 $\alpha\beta$ T cells (Figures 3B, 3C, and S4). C4 was further enriched for genes associated with cytotoxic effector functions (*PRF1*, *GZMH*, *GZMB*, and *GZMM*), motility, migration and tissue residency (*CX3CR1*, *TGFBR3*, and *S100A10*), and regulators of cell state (*AHNAK*, *KLF3*, and *ZEB2*; Figure 3C; Table S4).

The remaining five cytotoxic subclusters did not associate with bacterial control by either statistical framework. Cytotoxic subclusters C1-3 were enriched for the expression of *CD8A* but not *CD8B* and elevated *TCRD*, implying that these cells possessed innate cytotoxic function (Figures 3B and 3C). C5, which expressed *CD8A* and *CD8B*, was distinguished by elevated expression of *GZMK* (Figure 3C), which has been recently described as a hallmark of immune dysfunction in inflammation (Mogilenko et al., 2021).

The functional complexity of these six subclusters, along with the common and distinct responses they represent, suggests a significant and underappreciated role for cytotoxic cells in TB granulomas.

Macrophage heterogeneity in Mtb granulomas

Although macrophages are responsible for much of the bacterial killing within granulomas, we did not observe any association between overall macrophage abundance and bacterial burden (Figures 2 and S5). Yet, like the T/NK cell cluster, the macrophage cluster had discernable substructure based on unbiased gene-expression analyses. Among the 27,670 macrophages, we identified nine subclusters (Table S4). The only cluster independently associated with bacterial control was Mac4, a subpopulation of macrophages enriched in high-burden lesions (q = 1.6×10^{-5}, Dirichlet p = 0.12; Figure S5E; Table S4). Upregulated genes in Mac4 included known interferon-response genes (*NFKBIA*, *IFI27*, *IFI30*), as well as more general pro-inflammatory processes (*IL1B*, *CXCL8*, *LYZ*) and complement activation (*C1QA*, *C1QB*, *C1QC*) (Figures S6A and S6B), consistent with the "macrophage IFN" phenotype described by Esaulova et al. as associated with poor bacterial control (Esaulova et al., 2021). Mac5 and Mac3, meanwhile, were the populations that most strongly expressed genes that have been described as characteristic of epithelioid macrophages in zebrafish granulomas (q = 1.67×10^{-7} and q = 9.17×10^{-6}, respectively; Figures S6C–S6E) (Cronan et al., 2021). Mac5 was statistically significantly associated with high burden via multivariate Dirichlet regression analysis (Dirichlet p = 0.034), but not via univariate correlations with CFU (q = 0.31; Figure S9E).

Defining trajectories of bacterial burden and granuloma phenotype

To further understand the temporal emergence of variations in bacterial burden and granuloma states, we evaluated how cellular identities and compositions track with time. Here, we leveraged a scRNA-seq discovery dataset from six granulomas isolated at four weeks p.i. from two separate macaques (Figures 5A–5C). Four weeks is the earliest timepoint at which we can reliably identify granulomas by imaging; these lesions are by definition early appearing and thus likely to be high burden at 10 weeks p.i. However, they were captured at an earlier point in their development and therefore might be considered more analogous to late-appearing lesions at the 10-week timepoint (i.e., those first detected four weeks prior). We defined cell-type-specific "burden-associated gene sets" based on differentially expressed genes between 10-week p.i. high- vs. low-burden granulomas. Scoring four-week p.i. granuloma cells for these gene sets demonstrated that the T cell and macrophage phenotypes were more concordant with the early, high-burden lesions at 10 weeks than the later-appearing, more restrictive lesions (Figures 5D and 5E).

These data suggest a measure of stability in the cellular microenvironment between four and 10 weeks in early-appearing granulomas. They further indicate that the differences between high- and low-burden granulomas at 10 weeks do not simply reflect lesions at different stages in the same maturation continuum. Instead, they suggest that late-appearing, low-burden granulomas reflect a different path. We propose that late-appearing granulomas develop in the context of an emerging adaptive immune response, can recruit adaptive T cells quickly, and are better able to kill Mtb. This model is consistent with our published work showing robust clearance of Mtb in a reinfection model (Cadena et al., 2018). However, we also acknowledge the potential for bacterial burden to shape granuloma phenotype, with burden and multicellular microenvironment each having the capacity to influence one another, potentially in a self-reinforcing manner.

Cellular ecology of pulmonary TB granulomas

Given demonstrable differences in cellular composition across the bacterial burden spectrum, we wondered whether specific cell types co-occur in TB lung granulomas to collectively influence control. By using hierarchical clustering of pairwise correlations between cell type frequencies, we defined five groups of cell types whose collective abundances were associated across granulomas (Figure 6A; Table S5). Of these, group 2 (shown in red), which included mast cells, plasma cells, Mac4, and certain

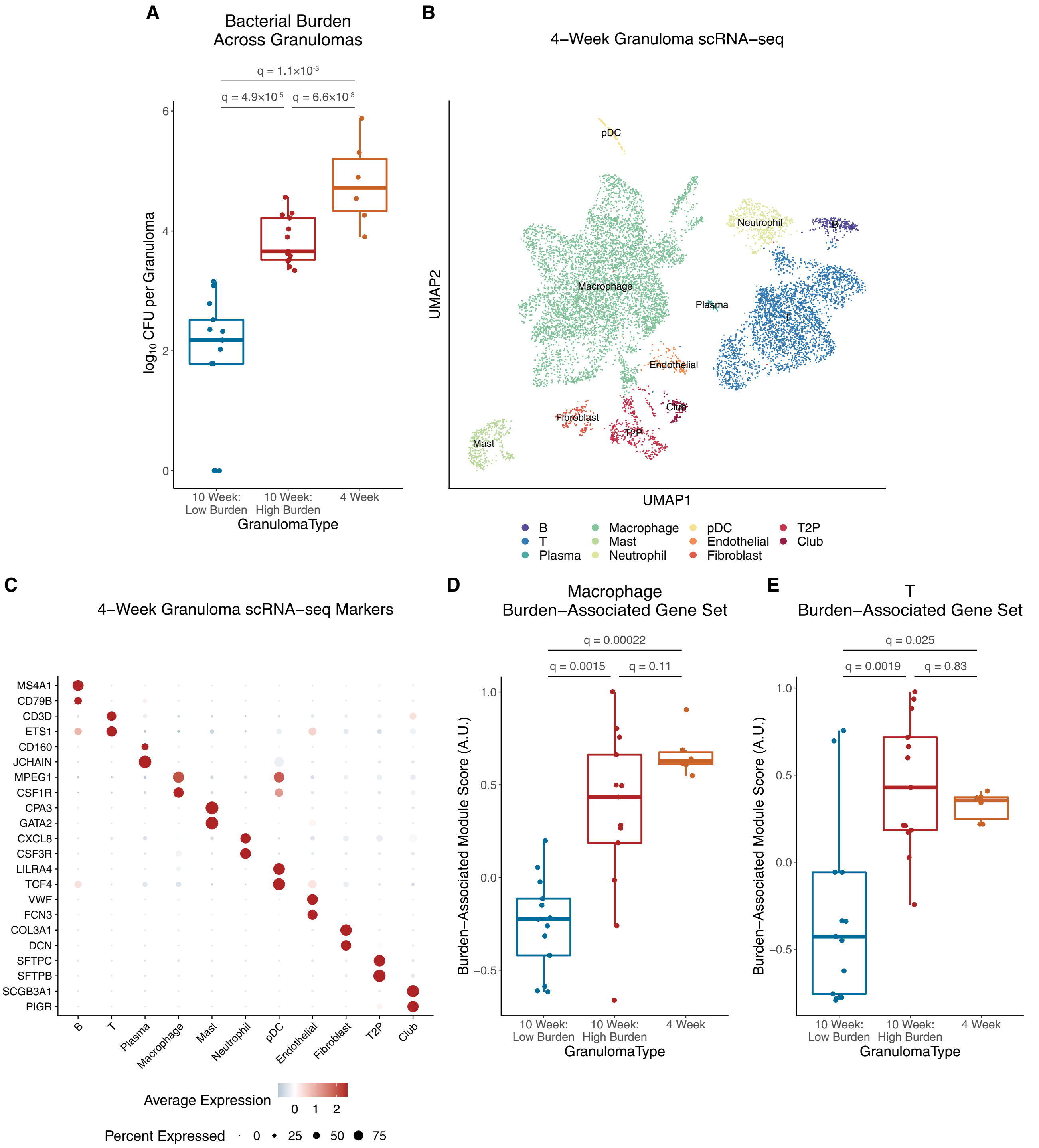

Figure 5. Profiling the temporal trajectory of granuloma development

(A) Comparison of bacterial burdens across timing of granuloma development and time p.i., using MWU test with Benjamini-Hochberg correction for multiple hypothesis testing.

(B) UMAP visualization of scRNA-seq data of 10,007 cells from six granulomas across two macaques at four weeks p.i.

(C) Expression levels of cluster-defining genes. Color intensity corresponds to level of gene expression, and size of dots represents the proportion of cells with non-zero expression in each cluster.

(D) Expression levels of macrophage burden-associated gene set, defined by using genes differentially expressed between macrophages in 10-week-p.i. high-burden and 10-week-p.i. low-burden granulomas; boxplot with median, interquartile range, and whiskers extending a maximum of 1.5*IQR; MWU test with Benjamini-Hochberg correction for multiple hypothesis testing.

(E) Expression levels of T cell burden-associated gene set, defined by using genes differentially expressed between T cells in 10-week p.i. high-burden and 10-week p.i. low-burden granulomas; MWU test with Benjamini-Hochberg correction for multiple hypothesis testing.

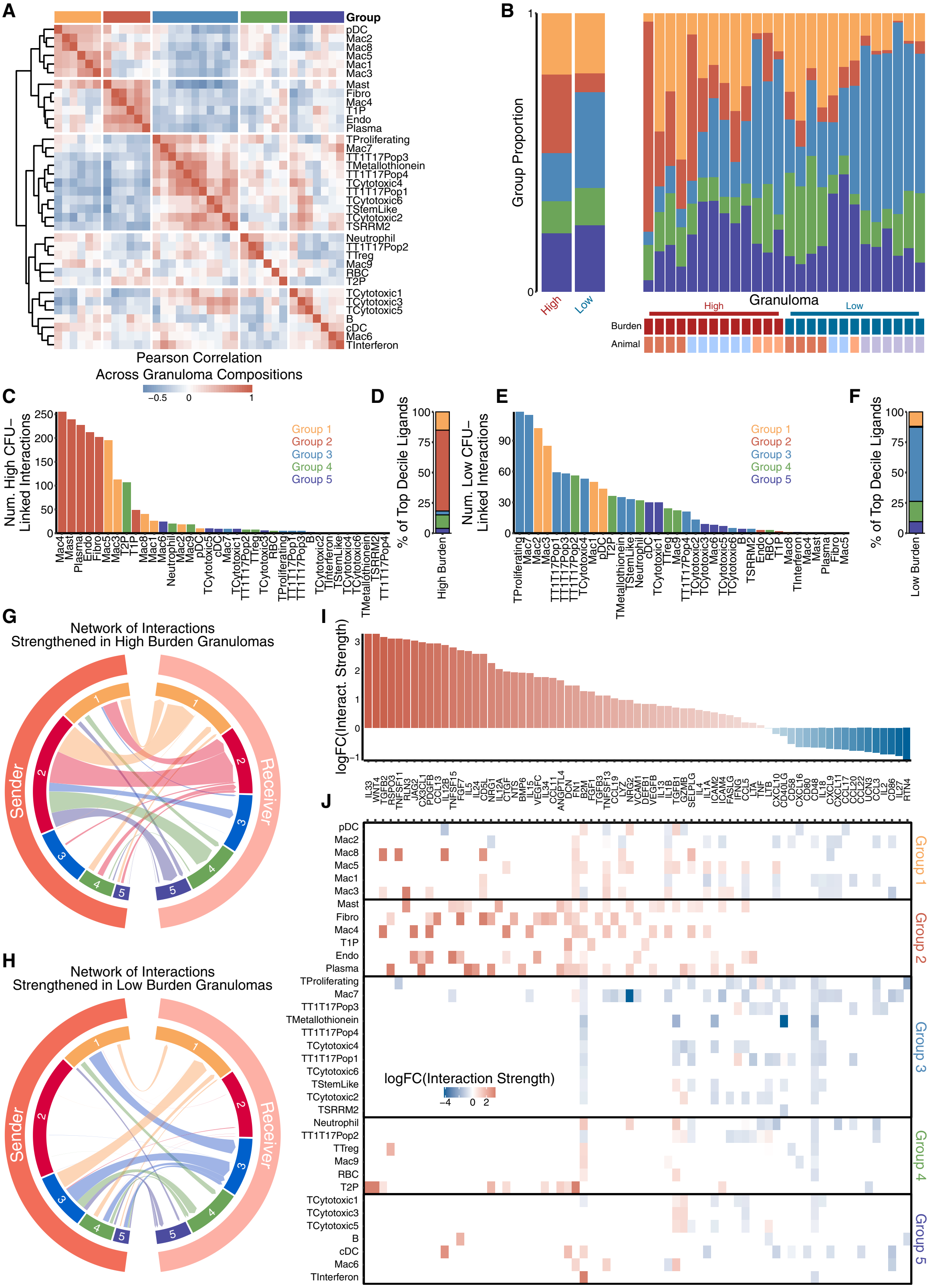

Figure 6. Cellular ecosystem in TB lung granulomas

(A) Pairwise Pearson correlation values of cell type proportions across 26 10-week p.i. granulomas.

(B) Composition of each granuloma by cell type group. Left shows grouped high- and low-burden granulomas; right bar graph is split by granuloma.

(legend continued on next page)

stromal populations, was significantly expanded in high-bacterial-burden granulomas ($p = 3 \times 10^{-4}$, MWU test; Figure 6B; Table S5). Group 3 (shown in blue) was significantly more abundant in low-bacterial-burden granulomas ($p = 0.026$; Figure 6B; Table S5) and consisted of many T cell subclusters/subpopulations, including stem-like; cytotoxic subclusters C2, C4, and C6; metallothionein; proliferating; SRRM2+; and T1-T17 subpopulations 1, 3, and 4, as well as Mac7. This macrophage subset was distinguished in part, by expression of the immunomodulatory genes *IDO* and *CHIT* (encoding chitotriosidase), which is abundantly produced by lipid-laden macrophages in other conditions such as Gaucher's disease, Niemenn-Pick disease, and atherosclerosis (Barone et al., 2007; Yap et al., 2020).

Distinct cellular ecosystems associate with granuloma-level bacterial burden

To further explore how specific cellular compositions might underpin differential bacterial control, we examined putative cell-cell interactions within each granuloma (STAR Methods). High-bacterial-burden lesions were dominated by signals sent by group 2 cell types (i.e., mast, fibroblast, endothelial, plasma, type 1 pneumocyte, and Mac4); these cell types displayed the highest counts of high-burden-linked interactions as well as those most strengthened in high-burden granulomas ($p < 2.2 \times 10^{-16}$, binomial test) (Figures 6C and 6D). In contrast, interactions in low-burden granulomas more evenly involved groups 1, 3, 4, and 5, with group 3 showing the strongest enrichment for signaling activity strengthened in low-burden granulomas ($p < 2.2 \times 10^{-16}$) (Figures 6E and 6F). We further examined shifts in intercellular interaction network topology by quantifying the sender and receiver activity associated with different degrees of bacterial burden. In high-burden granulomas, group 2 cell types were the key source of intercellular signals (Figure 6G), with strong intra-group 2 signaling. This suggests that high-burden lesions are driven by self-reinforcing interactions amongst group 2 cell types (e.g., between mast cells, plasma cells, fibroblasts, and endothelial cells). In contrast, in low-burden granulomas, we found only sparse contributions from group 2 cell types (Figure 6H); instead, low-burden granulomas were characterized by a more even distribution of signals stemming and terminating in groups 1, 3, 4, and 5 cell types, suggestive of a coordinated immune response involving multiple cellular subsets.

We next examined which specific axes of intercellular communication and, among whom, which were associated with varying bacterial control. Among the ligands whose interactions are most strengthened in high-burden granulomas, we identified genes implicated in fibrosis (e.g, *FGF1*, *PDGFB*, *CTGF*, *FGF7*, *IL34*), vascular remodeling (*VEGFB*, *VEGFC*, *ANGPTL4*) and TGFβ signaling (*TGFB2*, *TGFB3*, *BMP6*), suggestive of a wound-healing response (Figure 6I) (Joshi et al., 2020; Padela et al., 2008). In addition, we observed evidence of intercellular communication via genes implicated in type 2 immunity (*CCL11*, *CCL13*, *CD5L*, *IL4*, *IL5*, *IL13*, *IL24*) and allergy-linked inflammation (*CCL19*) (Nakano et al., 2019). These specific ligands were largely produced and received by group 2 cell types (with only sparse contributions from groups 3–5). Collectively, this supports a model where intra-group 2 signaling drives a self-reinforcing high-burden microenvironment via wound-healing-like responses and associated type 2 immune activity (Figure 6J). This interpretation was further supported by enrichment of pathways such as TGFβ, WNT, and FGF signaling, as well as organogenesis, epithelial/endothelial proliferation, and tissue-remodeling processes (Figure S6F); is consistent with prior descriptions of angiogenesis and fibrosis in human granulomas (McCaffrey et al., 2022; Polena et al., 2016); and supports the targeting of vasculature as a therapeutic direction for tuberculosis (Datta et al., 2015; Oehlers et al., 2015).

In contrast, low-burden granulomas exhibited cell-cell interactions consistent with type 1 immune responses (*CCL3*, *CXCL9/10/11*, *IL18*) and Th17 chemoattraction (*CXCL16*, *CCL20*), co-stimulatory molecules important in immune activation (*CD40LG*, *CD48*, *CD80*, *CD86*), and those involved in lymphocyte adhesion (*CD58*) (Figure 6I) (Li et al., 2013; Lim et al., 2008; Touzot et al., 2014). Importantly, signaling occurred between multiple T and macrophage cell subsets, suggesting that successful Mtb control required coordinated interactions across diverse innate and adaptive immune cell types.

Our cell-cell interaction analyses also indicated context-dependent roles for certain cell types and ligands. For instance, the macrophage-dominated group 1 was not statistically correlated with granuloma control in our compositional analyses (Figure 6A) but participated in the second-most interactions in both high- and low-burden granulomas (Figures 6B, 6C, 6E, 6G, and 6H). The idea of dual roles for group 1 cells was borne out by examination of the ligands produced by group 1 cell types in high- (*PDGFB*, *CD5L*, *TNFSF13*) and low-burden (*CXCL9/10/11*, *CD86*, *IL18*, *CCL20*) microenvironments (Figures 6I and 6J). Similarly, some individual ligands participated in interactions in both high- and low-burden granulomas, suggesting pleiotropic

(C) Number of interactions strengthened in high-burden granulomas, organized by sender cell clusters.

(D) Representation of each cell type group as sender cell population among the 10% of ligands most strengthened in high-burden granulomas.

(E) Number of interactions strengthened in low-burden granulomas, organized by sender cell clusters.

(F) Representation of each cell type group as sender among the 10% of ligands most strengthened in low-burden granulomas.

(G) Network of interactions across cell type groups, subsetted to interactions strengthened in high-burden granulomas. Widths of arcs are proportional to number of interactions between cell type groups, and widths are on same scale as for inset (H). n = 2,899 statistically significant interactions, 1,837 of which were strengthened in high-burden granulomas.

(H) Network of interactions across cell type groups, subsetted to only highlight interactions strengthened in low-burden granulomas. Widths of arcs are proportional to number of interactions between cell type groups, and widths are on same scale as for inset (G). n = 2,899 statistically significant interactions, 1,062 of which were strengthened in low-burden granulomas.

(I) Overall high-vs-low granuloma burden fold-change of interactions strengths of key ligands, averaged across all statistically significant interactions.

(J) Cell-cluster-specific interaction strength fold changes of each ligand, averaged across all statistically significant interactions where each cell cluster was the sender population.

See also Figure S6; Table S5.

effects. As one specific example, IL-1's effects on Mtb vary based on disease stage and model (Juffermans et al., 2000; Law et al., 1996; Mayer-Barber et al., 2014; Mishra et al., 2013; Zhang et al., 2014). Based on our analyses, *IL1A* and *IL1B* each mediated interactions associated with both high and low bacterial burden but were derived from different sender cell populations in the two instances. Thus, our intercellular interaction analyses uncover axes of cellular plasticity and ligand pleiotropy across granuloma microenvironments, important for improved understanding and therapeutic modulation of Mtb (Keshavjee and Farmer, 2012).

DISCUSSION

Within an individual with Mtb infection, distinct granulomas can achieve sterilizing immunity, immune standoff, or frank immune failure (Flynn, 2006; Flynn and Klein, 2011; Lin et al., 2009, 2014b). In NHPs, which most closely recapitulate human Mtb infection and disease (Coleman et al., 2014a), this heterogeneity provides an opportunity to define the cellular and molecular factors that correlate with bacterial control to identify potential host-directed prevention and cure strategies for TB. Here, our data substantiate a model where the state of the surrounding host cellular ecosystem helps inform a granuloma's infection trajectory, leading to long-term, stable states which either permit or restrict bacterial survival.

To exemplify the links between cellular composition, gene expression, intercellular interaction patterns, and bacterial burden, we highlight mast cells: most abundant in high-burden lesions, mast cells were major producers of type 2 cytokines, especially *IL4*, *IL5*, and *IL13*, which are down-modulators of lymphocyte and macrophage antimicrobial activity, including inhibiting the cytolytic functions of $CD8^+$ T cells (Kienzle et al., 2005; Wijesundara et al., 2013). However, IL-4 and IL-13 have broader functions in the context of wound healing. Indeed, the cellular interactions in high-burden granulomas revealed both specific signaling molecules (e.g., FGF1 from type 1 pneumocytes, PDGFB from endothelial cells, ANGPTL4 from plasma and mast cells, among others) and broad pathways that reflected fibrosis, metabolic remodeling, and angiogenesis. Collectively, these data suggest a cascade of interactions in early-appearing granulomas with failed control, whereby an initially permissive environment is reinforced by a tissue-remodeling response that seeks to limit and wall off pathologic activity. Although more detailed studies on the roles of wound-healing responses and tissue remodeling in TB are indicated, these features could represent critical targets for host-directed therapies that need to not only enhance restrictive adaptive immune responses but also address the maladaptive features of microenvironments permissive to granuloma persistence (Ahidjo et al., 2016).

The strongest cellular correlate of bacterial control was a subcluster of cells with transcriptional features of both type 1 and type 17 T cells that was expanded in granulomas with bacterial control. Previous studies have revealed a prominent role for CD4 Th1 and Th17 cytokines in control of Mtb infection, including IFN-γ, TNF, and IL-17 (Algood et al., 2005; Green et al., 2013; Khader et al., 2007; Khader and Gopal, 2010; Lin et al., 2007; Lyadova and Panteleev, 2015; Millington et al., 2007; O'Garra et al., 2013; Scriba et al., 2017), and studies in NHP granulomas suggest an association between T1 and T17 cytokine expression and bacterial burden (Gideon et al., 2015). In addition, in murine models, BHLHE40 is required for control of Mtb infection, as a repressor of IL-10 production (Huynh et al., 2018). Aspects of these data are consistent with recent observations that granulomas established in immune-primed environments—e.g., existing Mtb infection (Cadena et al., 2018) or intravenous or intrabronchial BCG vaccination—are characterized by Th1/17 expression patterns that are associated with protection (Darrah et al., 2020; Dijkman et al., 2019); however, we extend these findings, defining appreciable substructure among the T1-T17 subcluster of relevance to control. The *CD4* T1-T17 subpopulation (subpopulation 1) is most consistent with published descriptions of Th1/17 cells (e.g., Th1* or ex-Th17) (Acosta-Rodriguez et al., 2007; Amezcua Vesely et al., 2019; Becattini et al., 2015; Lee et al., 2021; Lindestam Arlehamn et al., 2013; Mahnke et al., 2013; Nikitina et al., 2018). These cells could represent precursors to long-lived tissue memory, which has been shown to play a crucial protective role in autoimmunity, bacterial control, and memory immune responses to pathogens (Amezcua Vesely et al., 2019; Liang et al., 2015; van Hamburg and Tas, 2018; Wacleche et al., 2016), including Mtb infection. A recent study using flow cytometry and immunohistochemistry in Mtb-infected rhesus macaques supports an association of Th1 (IFNγ+) and Th17 (IL-17+) cells in lung tissue with latent infection (Shanmugasundaram et al., 2020); in contrast, another study using scRNA-seq reported activated CD4 and CD8 T cells including Th1 and Th17 in the lung tissue of macaques with pulmonary TB (Esaulova et al., 2021). The *CD8* subsets within the T1-T17 subcluster (subpopulations 2 and 3), meanwhile, have not been described previously. The former of these was strongly associated with bacterial control and could represent an immunologic paradigm that can be exploited for vaccine development.

Our data also revealed a *CD4*- and *CD8*-expressing T cell subcluster associated with low-burden granulomas that resembles stem-like T cells (Ahmed et al., 2016; Caccamo et al., 2018; Cartwright et al., 2016; Fuertes Marraco et al., 2015; Gattinoni et al., 2011; Mateus et al., 2015; Todryk, 2018). We hypothesize that these cells could be a source of T cell renewal in granulomas and could differentiate into the various functional subsets we observe within them. It is possible, however, that these represent memory T cells that are not specific for Mtb antigens but migrate to the granuloma in response to inflammation and/or chemokine gradients. Indeed, flow-cytometry-based studies support that a majority of T cells in granulomas do not respond to Mtb antigens by making cytokines and do not display hallmarks of exhaustion (Gideon et al., 2015; Sakai et al., 2016; Wong et al., 2018).

Although both CD4 and CD8 T cells have been implicated in control of Mtb infection, the cytotoxic function of lymphocytes in Mtb infection has been relatively understudied. However, we also found previously unappreciated complexity among granuloma cytotoxic cells of relevance to bacterial control. Of these, cytotoxic subcluster 4, which was enriched for CD8 $\alpha\beta$ T cells and defined by expression of several granzymes and perforin, likely represents cytotoxic effector T cells that target infected cells and is associated with low-burden granulomas. Our findings contrast with those in model systems like mice,

which notably do not have the capacity to sterilize sites of infection and whose CD8 T cells also do not express granulysin (Hojo-Souza et al., 2020). However, our findings are consistent with a recent study on lung tissue from Mtb-infected macaques which also found evidence of cytotoxic molecule expression associated with controlled infection (Esaulova et al., 2021).

Our analyses not only revealed sets of biological pathways utilized in the host cells of high- vs. low-burden granulomas but also assigned roles to the specific cell types that drive these signaling patterns. In particular, the strong internal signaling among group 2 cell types and comparatively weaker cross-talk to other groups in early lesions could drive establishment of a cellular ecosystem dominated by type 2 immune and wound-healing responses that preclude effective T cell engagement and conversion to a more restrictive state. By comparison, in late-appearing lesions, primed T cell populations, in concert with different innate populations, could use a variety of pro-inflammatory and pro-activation interactions to control Mtb growth or dissemination; a similar phenomenon might explain how infection with Mtb can protect against subsequent reinfection (Cadena et al., 2018) even in the presence of ongoing original infection, by locally recruiting adaptive responses that can act before self-reinforcing group 2 responses work to limit pathology.

We note that the contrasting microenvironments revealed through our analyses can occur within the same individual. Knowledge of intercellular networks underlying granuloma stability will spur future research efforts to identify and manipulate linchpins that serve as key nodes in limiting or enhancing the efficacy of therapeutic and prophylactic measures. For instance, there might be a potential therapeutic role for IL-15 super-agonists in clinical development that can drive expansion of cytotoxic populations (Fujii et al., 2018; Knudson et al., 2019). We also found strong enrichment for the expression of distinct neuro-hormonal modulators by group 2 (*NRG1*, *RLN3*, *NTS*) and group 3 cells (*UCN3*), as well as associations with transcriptional targets of sex hormones. Ligands and receptors implicated in low-burden interactions were enriched for targets of several neuromodulatory agents, including buprenorphine and fluoxetine, where serotonin reuptake inhibitors have already been identified in screens for host-acting compounds that improve macrophage control of Mtb, supporting potential for their further investigation (Heemskerk et al., 2021; Stanley et al., 2014).

In summary, our scRNA-seq investigation revealed cellular and molecular features that dynamically associate with natural control of Mtb in pulmonary granulomas. Interactive visualizations of all scRNA-seq data and associated metadata are hosted through the Broad Single-Cell Portal for further exploration and re-analyses (see data and code availability). Beyond recapitulating canonical correlates, our analysis defined nuanced, actionable, innate as well as adaptive functional cell states and shed light on essential dynamics among host-pathogen interactions (Iwasaki and Medzhitov, 2015). Collectively, our data substantiate a model where high Mtb burden within granulomas is dictated locally by type 2 immunity and tissue-protective (wound-healing) responses that seek to maintain essential tissue functionality at the expense of creating a niche for bacterial persistence. In granulomas that form later in infection, and, therefore, in the context of an adaptive immune response, this balance is tipped toward bacterial control by the emergence of adaptive T1-T17 and cytotoxic responses, with interactions involving innate immune cell types enabling sufficient infiltration and activation of these T cell subsets. As a result, successful immune coordination across cell types in late-forming granulomas could obviate the self-reinforcing type 2 immune/wound-healing responses that would otherwise exclude immune effector functions needed for Mtb control. We also identified cell types and ligands that participate in both high- and low-burden granulomas potentially indictive of phenotypic plasticity and pleiotropic effects that might both be molded by and (in turn) reinforce distinct, pathology-associated granuloma microenvironments. Such a framework is consistent with previous observations of natural (Cadena et al., 2018) or induced (Darrah et al., 2020) control and supports the need to look to combinatorial host-directed paradigms for the development of efficacious therapeutic and prophylactic measures.

Moving beyond the perspective of individual molecular targets, our work highlights the importance of the complexities of divergent host cellular ecosystems in driving Mtb persistence or control. By defining and nominating several putative axes of intra- and intercellular signaling associated with contrasting Mtb outcomes, our work provides a foundation for enabling effective manipulation of the properties and states of complex cellular ecosystems, therapeutically relevant destabilization of pathologic molecular environments to enable adaptive immune access and fundamental connections to other inflammatory and infectious diseases that affect epithelial barrier tissues (Hughes et al., 2020; Ordovas-Montanes et al., 2018).

Limitations of the study

Granulomas are inherently heterogeneous and include necrotic debris, requiring robust technical correction and quality control; this results in an analysis of only high-quality cells. Because only a fraction of cells from each granuloma were analyzed, proportions might not have reflected the true composition of cells within a granuloma and could be skewed toward lymphocytes, highlighting the importance of orthogonal validations. In bulk RNA-sequencing analysis of a separate set of dissociated early and late granulomas, we observe generally similar trends in cell-type composition, supporting our conclusions; similar studies will need to be performed in undigested granulomas to account for dissociation artifacts. In the absence of prior comparable studies on macaque granulomas, we could not predict *a priori* the granuloma diversity uncovered by scRNA-seq profiling, even before considering potential genetic differences in both host and pathogen. Even with these considerations, the sample size of this study was sufficient to reveal features of host responses linked to Mtb persistence or control that could inform future efforts across the TB community. Furthermore, knowledge of T cell antigen specificity could serve to prioritize T cell subsets for their relevance to bacterial control but would require the development of new methodologies that allow analysis of very small numbers of primary cells and a very large antigenic repertoire against the major histocompatibility complex diversity of outbred macaques. Relatedly, the transcriptomic granuloma landscape investigated here is from a pair of (albeit pivotal) time points, including granulomas at the earliest timepoint of reliable, non-invasive detection and granulomas across a spectrum of growth trajectories when bacterial killing can be identified in some but not all granulomas.

It is likely that expression of certain genes that arise early in infection and then are downregulated as infection progress will be missed, as will some populations critical to guiding overall granuloma outcome. More generally, matched profiling of additional timepoints p.i., along with analysis of lung tissue and granulomas from vaccinated or reinfected and protected animals, will provide a more complete picture of the temporal control of Mtb in granulomas and is the subject of future work.

STAR★METHODS

Detailed methods are provided in the online version of this paper and include the following:

- KEY RESOURCES TABLE
- RESOURCE AVAILABILITY
  - Lead contact
  - Materials availability
  - Data and code availability
- EXPERIMENTAL MODEL AND SUBJECT DETAILS
  - Research animals
- METHOD DETAILS
  - Serial PET-CT Imaging
  - Necropsy
  - Bacterial burden
  - Chromosomal equivalents, CEQ
  - Immunohistochemistry analysis
  - Flow cytometry
  - Single-cell RNA-sequencing (scRNA-seq)
  - Bulk RNA sequencing
  - Sequencing and alignment
- QUANTIFICATION AND STATISTICAL ANALYSIS
  - Data processing and quality control
  - Correction for residual background contamination
  - Separation of doublets
  - Integrated cell type classification
  - Cell type assignment of proliferating cells
  - Filtering of soup-defining transcripts
  - Sub-clustering of granuloma unified T and NK cells
  - Annotation of T /NK subclusters
  - Sub-clustering of granuloma macrophages
  - Classification of macrophage populations
  - Deconvolution of bulk RNA-sequencing data
  - Co-variation in granuloma composition
  - Cell-communication analysis
  - Statistical methods

SUPPLEMENTAL INFORMATION

Supplemental information can be found online at https://doi.org/10.1016/j.immuni.2022.04.004.

ACKNOWLEDGMENTS

We are grateful to the research and veterinary technicians: Chelsea Chedrick, Carolyn Bigbee, Nicholas Schindler, Mark Rogers, Tara Rutledge, Chelsea Causgrove, and Brianne Stein in the Flynn lab who assisted with this work, as well as helpful discussions with members of the Flynn, Scanga, Mattila, Lin, and Shalek laboratories. We also thank the efforts of the University of Pittsburgh Division of Laboratory Animal Research technicians for husbandry of the animals. We acknowledge the following funding sources: Bill and Melinda Gates Foundation (OP1139972, A.L., S.M.B., S.M.F., J.L.F., A.K.S.; OPP1202327, A.K.S.), Searle Scholars Program (A.K.S.), The Beckman Young Investigator Program (A.K.S.), Sloan Fellowship in Chemistry (A.K.S.). NIH (5U24AI118672 and BAA-NIAID-NIHAI201700104, A.K.S.), American Lung Association (RG571577, H.P.G.), F30-AI143160 (T.K.H.), NIH T32A1065380 (N.L.G.), NSF GRFP grant (C.N.T., S.K.N., 1122374). Fannie and John Hertz Foundation Fellowship (C.N.T.), Wellcome Trust Fellowship award 210662/Z/16/Z (A.L.), Koch Institute Support (core) grant P30-CA14051 from the National Cancer Institute (C.L.), NIH CFAR P30 AI060354 (B.B.), NIH R01A1022553 (B.B.), T32 A1007387 (J.R.), and NIH K12 (C.W.).

AUTHOR CONTRIBUTIONS

Conceptualization, J.L.F., S.M.F., and A.K.S.; Data curation, H.P.G., T.K.H., F.F.H., P.M., A.G.W., N.L.G., and A.L.; Formal analysis, H.P.G., T.K.H., C.N.T., N.L.F., F.F.H., and A.G.W.; Methodology, H.P.G., T.K.H., C.N.T., M.H.W., A.A.T., T.M.G., F.F.H., C.K., P.M., A.G.W., S.K.N., H.J.B., B.B., and J.C.L.; Investigation, H.P.G., T.K.H., C.N.T., M.H.W., A.A.T., T.G., F.F.H., J.W., C.K., J.M.P., P.M., A.G.W., S.K.N., H.J.B., S.K.C.G., A.M., K.V.P., C.L.A., C.T.C., J.A.T., L.J.F., H.J.B., P.L.L., S.I., J.Y.P., J.M.R., A.S., and J.O.M.; Visualization, H.P.G., T.K.H., and C.N.T.; Validation, H.P.G., T.K.H., N.L.G., K.N., C.G.W., and S.I.; Resources, J.L.F., S.M.F., A.K.S., J.C.L., R.M., and A.L.; Funding acquisition: J.L.F., S.M.F., and A.K.S.; Project administration, C.A.S.; Supervision, J.L.F., S.M.F., A.K.S., S.M.B., B.D.B., A.L., J.C.L., and B.B.; Writing – original draft, H.P.G., T.K.H., S.M.B., J.L.F., S.M.F., and A.K.S.; Writing – review & editing, H.P.G., T.K.H., C.N.T., M.C., S.M.B., J.L.F., S.M.F., and A.K.S.

DECLARATION OF INTERESTS

A.K.S. reports compensation for consulting and/or SAB membership from Merck, Honeycomb Biotechnologies, Cellarity, Repertoire Immune Medicines, Third Rock Ventures, Hovione, Relation Therapeutics, FL82, Empress Therapeutics, Ochre Bio, and Dahlia Biosciences.C.L. is a shareholder and consultant for Honeycomb Biotechnologies. T.K.H. is a shareholder and consultant for nference, inc.

INCLUSION AND DIVERSITY

One or more of the authors of this paper self-identifies as an underrepresented ethnic minority in science. One or more of the authors of this paper self-identifies as a member of the LGBTQ+ community. While citing references scientifically relevant for this work, we also actively worked to promote gender balance in our reference list.

Received: October 12, 2021
Revised: February 8, 2022
Accepted: April 7, 2022
Published: April 27, 2022

REFERENCES

Acosta-Rodriguez, E.V., Rivino, L., Geginat, J., Jarrossay, D., Gattorno, M., Lanzavecchia, A., Sallusto, F., and Napolitani, G. (2007). Surface phenotype and antigenic specificity of human interleukin 17-producing T helper memory cells. Nat. Immunol. *8*, 639–646. https://doi.org/10.1038/ni1467.

Ahidjo, B.A., Maiga, M.C., Ihms, E.A., Maiga, M., Ordonez, A.A., Cheung, L.S., Beck, S., Andrade, B.B., Jain, S., and Bishai, W.R. (2016). The antifibrotic drug pirfenidone promotes pulmonary cavitation and drug resistance in a mouse model of chronic tuberculosis. JCI Insight *1*, e86017. https://doi.org/10.1172/jci.insight.86017.

Ahmed, R., Roger, L., Costa Del Amo, P., Miners, K.L., Jones, R.E., Boelen, L., Fali, T., Elemans, M., Zhang, Y., Appay, V., et al. (2016). Human stem cell-like memory T cells are maintained in a state of dynamic flux. Cell Rep. *17*, 2811–2818. https://doi.org/10.1016/j.celrep.2016.11.037.

Scott Algood, H.M., Lin, P.L., and Flynn, J.L. (2005). Tumor necrosis factor and chemokine interactions in the formation and maintenance of granulomas in

tuberculosis. Clin. Infect. Dis. *41*, S189–S193. https://doi.org/10.1086/429994.

Amezcua Vesely, M.C., Pallis, P., Bielecki, P., Low, J.S., Zhao, J., Harman, C.C.D., Kroehling, L., Jackson, R., Bailis, W., Licona-Limon, P., et al. (2019). Effector TH17 cells give rise to long-lived TRM cells that are essential for an immediate response against bacterial infection. Cell *178*, 1176–1188.e15. https://doi.org/10.1016/j.cell.2019.07.032.

Barone, R., Sotgiu, S., and Musumeci, S. (2007). Plasma chitotriosidase in health and pathology. Clin. Lab. *53*, 321–333.

Becattini, S., Latorre, D., Mele, F., Foglierini, M., De Gregorio, C., Cassotta, A., Fernandez, B., Kelderman, S., Schumacher, T.N., Corti, D., et al. (2015). Functional heterogeneity of human memory CD4+ T cell clones primed by pathogens or vaccines. Science *347*, 400–406. https://doi.org/10.1126/science.1260668.

Caccamo, N., Joosten, S.A., Ottenhoff, T.H.M., and Dieli, F. (2018). Atypical human effector/memory CD4(+) T cells with a naive-like phenotype. Front. Immunol. *9*, 2832. https://doi.org/10.3389/fimmu.2018.02832.

Cadena, A.M., Flynn, J.L., and Fortune, S.M. (2016). The importance of first impressions: early events in Mycobacterium tuberculosis infection influence outcome. mBio *7*. e00342–00316. https://doi.org/10.1128/mBio.00342-16.

Cadena, A.M., Hopkins, F.F., Maiello, P., Carey, A.F., Wong, E.A., Martin, C.J., Gideon, H.P., DiFazio, R.M., Andersen, P., Lin, P.L., et al. (2018). Concurrent infection with Mycobacterium tuberculosis confers robust protection against secondary infection in macaques. Plos Pathog *14*, e1007305. https://doi.org/10.1371/journal.ppat.1007305.

Canetti, G. (1955). The Tubercle Bacillus in the Pulmonary Lesion of Man: Histobacteriology and Its Bearing on the Therapy of Pulmonary Tuberculosis (Springer).

Cartwright, E.K., Palesch, D., Mavigner, M., Paiardini, M., Chahroudi, A., and Silvestri, G. (2016). Initiation of antiretroviral therapy restores CD4+ T memory stem cell homeostasis in simian immunodeficiency virus-infected macaques. J. Virol. *90*, 6699–6708. https://doi.org/10.1128/JVI.00492-16.

Coleman, M.T., Chen, R.Y., Lee, M., Lin, P.L., Dodd, L.E., Maiello, P., Via, L.E., Kim, Y., Marriner, G., Dartois, V., et al. (2014a). PET/CT imaging reveals a therapeutic response to oxazolidinones in macaques and humans with tuberculosis. Sci. Transl. Med. *6*, 265ra167. https://doi.org/10.1126/scitranslmed.3009500.

Coleman, M.T., Maiello, P., Tomko, J., Frye, L.J., Fillmore, D., Janssen, C., Klein, E., and Lin, P.L. (2014b). Early Changes by (18)Fluorodeoxyglucose positron emission tomography coregistered with computed tomography predict outcome after Mycobacterium tuberculosis infection in cynomolgus macaques. Infect. Immun. *82*, 2400–2404. https://doi.org/10.1128/IAI.01599-13.

Collins, A.M., and Jackson, K.J.L. (2013). A temporal model of human IgE and IgG antibody function. Front. Immunol. *4*, 235. https://doi.org/10.3389/fimmu.2013.00235.

Cronan, M.R., Hughes, E.J., Brewer, W.J., Viswanathan, G., Hunt, E.G., Singh, B., Mehra, S., Oehlers, S.H., Gregory, S.G., Kaushal, D., and Tobin, D.M. (2021). A non-canonical type 2 immune response coordinates tuberculous granuloma formation and epithelialization. Cell *184*, 1757–1774.e14. https://doi.org/10.1016/j.cell.2021.02.046.

Darrah, P.A., Zeppa, J.J., Maiello, P., Hackney, J.A., Wadsworth, M.H., 2nd, Hughes, T.K., Pokkali, S., Swanson, P.A., 2nd, Grant, N.L., Rodgers, M.A., et al. (2020). Prevention of tuberculosis in macaques after intravenous BCG immunization. Nature *577*, 95–102. https://doi.org/10.1038/s41586-019-1817-8.

Datta, M., Via, L.E., Kamoun, W.S., Liu, C., Chen, W., Seano, G., Weiner, D.M., Schimel, D., England, K., Martin, J.D., et al. (2015). Anti-vascular endothelial growth factor treatment normalizes tuberculosis granuloma vasculature and improves small molecule delivery. Proc. Natl. Acad. Sci. U S A. *112*, 1827–1832. https://doi.org/10.1073/pnas.1424563112.

Diedrich, C.R., Rutledge, T., Maiello, P., Baranowski, T.M., White, A.G., Borish, H.J., Karell, P., Hopkins, F., Brown, J., Fortune, S.M., et al. (2020). SIV and Mycobacterium tuberculosis synergy within the granuloma accelerates the reactivation pattern of latent tuberculosis. Preprint at bioRxiv. https://doi.org/10.1101/2020.02.21.959353.

Dijkman, K., Sombroek, C.C., Vervenne, R.A.W., Hofman, S.O., Boot, C., Remarque, E.J., Kocken, C.H.M., Ottenhoff, T.H.M., Kondova, I., Khayum, M.A., et al. (2019). Prevention of tuberculosis infection and disease by local BCG in repeatedly exposed rhesus macaques. Nat. Med. *25*, 255–262. https://doi.org/10.1038/s41591-018-0319-9.

Ehlers, S., and Schaible, U.E. (2013). The granuloma in tuberculosis: dynamics of a host-pathogen collusion. Front. Immunol. *3*, 411. https://doi.org/10.3389/fimmu.2012.00411.

Esaulova, E., Das, S., Singh, D.K., Choreno-Parra, J.A., Swain, A., Arthur, L., Rangel-Moreno, J., Ahmed, M., Singh, B., Gupta, A., et al. (2021). The immune landscape in tuberculosis reveals populations linked to disease and latency. Cell Host Microbe *29*, 165–178.e8. https://doi.org/10.1016/j.chom.2020.11.013.

Flynn, J.L. (2006). Lessons from experimental Mycobacterium tuberculosis infections. Microbes Infect. *8*, 1179–1188. https://doi.org/10.1016/j.micinf.2005.10.033.

Flynn, J.L., and Klein, E. (2011). "Pulmonary Tuberculosis in Monkeys" in A Color Atlas of Comparative Pulmonary Tuberculosis Histopathology, J. Leong, V. Dartois, and T. Dick, eds. (Taylor & Francis Publishers: CRC Press), pp. 83–106.

Foreman, T.W., Mehra, S., LoBato, D.N., Malek, A., Alvarez, X., Golden, N.A., Bucsan, A.N., Didier, P.J., Doyle-Meyers, L.A., Russell-Lodrigue, K.E., et al. (2016). CD4+ T-cell-independent mechanisms suppress reactivation of latent tuberculosis in a macaque model of HIV coinfection. Proc. Natl. Acad. Sci. U S A *113*, E5636–E5644. https://doi.org/10.1073/pnas.1611987113.

Fuertes Marraco, S.A., Soneson, C., Delorenzi, M., and Speiser, D.E. (2015). Genome-wide RNA profiling of long-lasting stem cell-like memory CD8 T cells induced by Yellow Fever vaccination in humans. Genom Data *5*, 297–301. https://doi.org/10.1016/j.gdata.2015.06.024.

Fujii, R., Jochems, C., Tritsch, S.R., Wong, H.C., Schlom, J., and Hodge, J.W. (2018). An IL-15 superagonist/IL-15Rα fusion complex protects and rescues NK cell-cytotoxic function from TGF-β1-mediated immunosuppression. Cancer Immunol. Immunother. *67*, 675–689. https://doi.org/10.1007/s00262-018-2121-4.

Garcia-Rodriguez, K.M., Goenka, A., Alonso-Rasgado, M.T., Hernandez-Pando, R., and Bulfone-Paus, S. (2017). The role of mast cells in tuberculosis: orchestrating innate immune crosstalk? Front. Immunol. *8*, 1290. https://doi.org/10.3389/fimmu.2017.01290.

Gattinoni, L., Lugli, E., Ji, Y., Pos, Z., Paulos, C.M., Quigley, M.F., Almeida, J.R., Gostick, E., Yu, Z., Carpenito, C., et al. (2011). A human memory T cell subset with stem cell-like properties. Nat. Med. *17*, 1290–1297. https://doi.org/10.1038/nm.2446.

Gideon, H.P., Phuah, J., Junecko, B.A., and Mattila, J.T. (2019). Neutrophils express pro- and anti-inflammatory cytokines in granulomas from Mycobacterium tuberculosis-infected cynomolgus macaques. Mucosal Immunol. *12*, 1370–1381. https://doi.org/10.1038/s41385-019-0195-8.

Gideon, H.P., Phuah, J., Myers, A.J., Bryson, B.D., Rodgers, M.A., Coleman, M.T., Maiello, P., Rutledge, T., Marino, S., Fortune, S.M., et al. (2015). Variability in tuberculosis granuloma T cell responses exists, but a balance of pro- and anti-inflammatory cytokines is associated with sterilization. PLoS Pathog *11*, e1004603. https://doi.org/10.1371/journal.ppat.1004603.

Gierahn, T.M., Wadsworth, M.H., 2nd, Hughes, T.K., Bryson, B.D., Butler, A., Satija, R., Fortune, S., Love, J.C., and Shalek, A.K. (2017). Seq-Well: portable, low-cost RNA sequencing of single cells at high throughput. Nat. Methods *14*, 395–398. https://doi.org/10.1038/nmeth.4179.

Grant, N.L., Maiello, P., Klein, E., Lin, P.L., Borish, H.J., Tomko, J., Frye, L.J., White, A.G., Kirschner, D.E., Mattila, J.T., and Flynn, J.L. (2022). T cell transcription factor expression evolves as adaptive immunity matures in granulomas from Mycobacterium tuberculosis-infected cynomolgus macaques. Cell Reports. (in press). https://doi.org/10.1101/2022.01.25.477732.

Green, A.M., Difazio, R., and Flynn, J.L. (2013). IFN-gamma from CD4 T cells is essential for host survival and enhances CD8 T cell function during Mycobacterium tuberculosis infection. J. Immunol. *190*, 270–277. https://doi.org/10.4049/jimmunol.1200061.

CellPress
OPEN ACCESS

Guo, X., Zhang, Y., Zheng, L., Zheng, C., Song, J., Zhang, Q., Kang, B., Liu, Z., Jin, L., Xing, R., et al. (2018). Global characterization of T cells in non-small-cell lung cancer by single-cell sequencing. Nat. Med. *24*, 978–985. https://doi.org/10.1038/s41591-018-0045-3.

Han, X., Wang, R., Zhou, Y., Fei, L., Sun, H., Lai, S., Saadatpour, A., Zhou, Z., Chen, H., Ye, F., et al. (2018). Mapping the mouse cell atlas by microwell-seq. Cell *173*, 1307. https://doi.org/10.1016/j.cell.2018.05.012.

Heemskerk, M.T., Korbee, C.J., Esselink, J.J., Carvalho Dos Santo, C., van Veen, S., Gordijn, I.F., Vrieling, F., Verreck, F.A.W., Ottenhoff, T.H.M., Haks, M.C., et al. (2021). Repurposing diphenylbutylpiperidine-class antipsychotic drugs for host-directed therapy of Mycobacterium tuberculosis and Salmonella enterica infections. Sci. Rep. *11*, 19634. https://doi.org/10.1038/s41598-021-98980-z.

Hirota, K., Yoshitomi, H., Hashimoto, M., Maeda, S., Teradaira, S., Sugimoto, N., Yamaguchi, T., Nomura, T., Ito, H., Nakamura, T., et al. (2007). Preferential recruitment of CCR6-expressing Th17 cells to inflamed joints via CCL20 in rheumatoid arthritis and its animal model. J. Exp. Med. *204*, 2803–2812. https://doi.org/10.1084/jem.20071397.

Hojo-Souza, N.S., de Azevedo, P.O., de Castro, J.T., Teixeira-Carvalho, A., Lieberman, J., Junqueira, C., and Gazzinelli, R.T. (2020). Contributions of IFN-gamma and granulysin to the clearance of Plasmodium yoelii blood stage. PLoS Pathog *16*, e1008840. https://doi.org/10.1371/journal.ppat.1008840.

Hughes, T.K., Wadsworth, M.H., 2nd, Gierahn, T.M., Do, T., Weiss, D., Andrade, P.R., Ma, F., de Andrade Silva, B.J., Shao, S., Tsoi, L.C., et al. (2020). Second-strand synthesis-based massively parallel scRNA-seq reveals cellular states and molecular features of human inflammatory skin pathologies. Immunity *53*, 878–894.e7. https://doi.org/10.1016/j.immuni.2020.09.015.

Hunter, R.L. (2011). Pathology of post primary tuberculosis of the lung: an illustrated critical review. Tuberculosis (Edinb) *91*, 497–509. https://doi.org/10.1016/j.tube.2011.03.007.

Hunter, R.L. (2016). Tuberculosis as a three-act play: a new paradigm for the pathogenesis of pulmonary tuberculosis. Tuberculosis (Edinb) *97*, 8–17. https://doi.org/10.1016/j.tube.2015.11.010.

Huynh, J.P., Lin, C.C., Kimmey, J.M., Jarjour, N.N., Schwarzkopf, E.A., Bradstreet, T.R., Shchukina, I., Shpynov, O., Weaver, C.T., Taneja, R., et al. (2018). Bhlhe40 is an essential repressor of IL-10 during Mycobacterium tuberculosis infection. J. Exp. Med. *215*, 1823–1838. https://doi.org/10.1084/jem.20171704.

Ivanov, I.I., McKenzie, B.S., Zhou, L., Tadokoro, C.E., Lepelley, A., Lafaille, J.J., Cua, D.J., and Littman, D.R. (2006). The orphan nuclear receptor RORγt directs the differentiation program of proinflammatory IL-17+ T helper cells. Cell *126*, 1121–1133. https://doi.org/10.1016/j.cell.2006.07.035.

Iwasaki, A., and Medzhitov, R. (2015). Control of adaptive immunity by the innate immune system. Nat. Immunol. *16*, 343–353. https://doi.org/10.1038/ni.3123.

Jacobs, A.J., Mongkolsapaya, J., Screaton, G.R., McShane, H., and Wilkinson, R.J. (2016). Antibodies and tuberculosis. Tuberculosis (Edinb) *101*, 102–113. https://doi.org/10.1016/j.tube.2016.08.001.

Joshi, N., Watanabe, S., Verma, R., Jablonski, R.P., Chen, C.I., Cheresh, P., Markov, N.S., Reyfman, P.A., McQuattie-Pimentel, A.C., Sichizya, L., et al. (2020). A spatially restricted fibrotic niche in pulmonary fibrosis is sustained by M-CSF/M-CSFR signalling in monocyte-derived alveolar macrophages. Eur. Respir. J. *55*, 1900646. https://doi.org/10.1183/13993003.00646-2019.

Juffermans, N.P., Florquin, S., Camoglio, L., Verbon, A., Kolk, A.H., Speelman, P., van Deventer, S.J.H., and van der Poll, T. (2000). Interleukin-1 signaling is essential for host defense during murine pulmonary tuberculosis. J. Infect. Dis. *182*, 902–908. https://doi.org/10.1086/315771.

Kanagaratham, C., El Ansari, Y.S., Lewis, O.L., and Oettgen, H.C. (2020). IgE and IgG antibodies as regulators of mast cell and basophil functions in food allergy. Front. Immunol. *11*, 603050. https://doi.org/10.3389/fimmu.2020.603050.

Keshavjee, S., and Farmer, P.E. (2012). Tuberculosis, drug resistance, and the history of modern medicine. Engl. J. Med. *367*, 931–936. https://doi.org/10.1056/NEJMra1205429.

Khader, S.A., Bell, G.K., Pearl, J.E., Fountain, J.J., Rangel-Moreno, J., Cilley, G.E., Shen, F., Eaton, S.M., Gaffen, S.L., Swain, S.L., et al. (2007). IL-23 and IL-17 in the establishment of protective pulmonary CD4+ T cell responses after vaccination and during Mycobacterium tuberculosis challenge. Nat. Immunol. *8*, 369–377. https://doi.org/10.1038/ni1449.

Khader, S.A., and Gopal, R. (2010). IL-17 in protective immunity to intracellular pathogens. Virulence *1*, 423–427. https://doi.org/10.4161/viru.1.5.12862.

Kienzle, N., Olver, S., Buttigieg, K., Groves, P., Janas, M.L., Baz, A., and Kelso, A. (2005). Progressive differentiation and commitment of CD8+ T cells to a poorly cytolytic CD8low phenotype in the presence of IL-4. J. Immunol. *174*, 2021–2029. https://doi.org/10.4049/jimmunol.174.4.2021.

Knudson, K.M., Hicks, K.C., Alter, S., Schlom, J., and Gameiro, S.R. (2019). Mechanisms involved in IL-15 superagonist enhancement of anti-PD-L1 therapy. J. Immunother. Cancer *7*, 82. https://doi.org/10.1186/s40425-019-0551-y.

Kobayashi, T., Okamoto, S., Hisamatsu, T., Kamada, N., Chinen, H., Saito, R., Kitazume, M.T., Nakazawa, A., Sugita, A., Koganei, K., et al. (2008). IL23 differentially regulates the Th1/Th17 balance in ulcerative colitis and Crohn’s disease. Gut *57*, 1682–1689. https://doi.org/10.1136/gut.2007.135053.

Law, K., Weiden, M., Harkin, T., Tchou-Wong, K., Chi, C., and Rom, W.N. (1996). Increased release of interleukin-1 beta, interleukin-6, and tumor necrosis factor-alpha by bronchoalveolar cells lavaged from involved sites in pulmonary tuberculosis. Am. J. Respir. Crit. Care Med. *153*, 799–804. https://doi.org/10.1164/ajrccm.153.2.8564135.

Lee, W., Kingstad-Bakke, B., Kedl, R.M., Kawaoka, Y., and Suresh, M. (2021). CCR2 regulates vaccine-induced mucosal T-cell memory to influenza A virus. J. Virol *95*, e0053021. https://doi.org/10.1128/JVI.00530-21.

Lenaerts, A., Barry, C.E., 3rd, and Dartois, V. (2015). Heterogeneity in tuberculosis pathology, microenvironments and therapeutic responses. Immunol. Rev. *264*, 288–307. https://doi.org/10.1111/imr.12252.

Li, Q., Laumonnier, Y., Syrovets, T., and Simmet, T. (2013). Recruitment of CCR6-expressing Th17 cells by CCL20 secreted from plasmin-stimulated macrophages. Acta Biochim. Biophys. Sin. *45*, 593–600. https://doi.org/10.1093/abbs/gmt049.

Liang, Y., Pan, H.F., and Ye, D.Q. (2015). Tc17 cells in immunity and systemic autoimmunity. Int. Rev. Immunol. *34*, 318–331. https://doi.org/10.3109/08830185.2014.954698.

Liberzon, A., Subramanian, A., Pinchback, R., Thorvaldsdottir, H., Tamayo, P., and Mesirov, J.P. (2011). Molecular signatures database (MSigDB) 3.0. Bioinformatics *27*, 1739–1740. https://doi.org/10.1093/bioinformatics/btr260.

Lim, H.W., Lee, J., Hillsamer, P., and Kim, C.H. (2008). Human Th17 cells share major trafficking receptors with both polarized effector T cells and FOXP3+ regulatory T cells. J. Immunol. *180*, 122–129. https://doi.org/10.4049/jimmunol.180.1.122.

Lin, C.C., Bradstreet, T.R., Schwarzkopf, E.A., Jarjour, N.N., Chou, C., Archambault, A.S., Sim, J., Zinselmeyer, B.H., Carrero, J.A., Wu, G.F., et al. (2016). IL-1-induced Bhlhe40 identifies pathogenic T helper cells in a model of autoimmune neuroinflammation. J. Exp. Med. *213*, 251–271. https://doi.org/10.1084/jem.20150568.

Lin, C.C., Bradstreet, T.R., Schwarzkopf, E.A., Sim, J., Carrero, J.A., Chou, C., Cook, L.E., Egawa, T., Taneja, R., Murphy, T.L., et al. (2014a). Bhlhe40 controls cytokine production by T cells and is essential for pathogenicity in autoimmune neuroinflammation. Nat. Commun. *5*, 3551. https://doi.org/10.1038/ncomms4551.

Lin, P.L., Coleman, T., Carney, J.P.J., Lopresti, B.J., Tomko, J., Fillmore, D., Dartois, V., Scanga, C., Frye, L.J., Janssen, C., et al. (2013). Radiologic responses in cynomolgus macaques for assessing tuberculosis chemotherapy regimens. Antimicrob. Agents Chemother. *57*, 4237–4244. https://doi.org/10.1128/AAC.00277-13.

Lin, P.L., Ford, C.B., Coleman, M.T., Myers, A.J., Gawande, R., Ioerger, T., Sacchettini, J., Fortune, S.M., and Flynn, J.L. (2014b). Sterilization of granulomas is common in active and latent tuberculosis despite within-host variability in bacterial killing. Nat. Med. *20*, 75–79. https://doi.org/10.1038/nm.3412.

Lin, P.L., Myers, A., Smith, L., Bigbee, C., Bigbee, M., Fuhrman, C., Grieser, H., Chiosea, I., Voitenek, N.N., Capuano, S.V., et al. (2010). TNF neutralization results in disseminated disease during acute and latent M. tuberculosis infection with normal granuloma structure. Arthritis Rheum. *62*, 340–350. https://doi.org/10.1002/art.27271.

Lin, P.L., Pawar, S., Myers, A., Pegu, A., Fuhrman, C., Reinhart, T.A., Capuano, S.V., Klein, E., and Flynn, J.L. (2006). Early events in Mycobacterium tuberculosis infection in cynomolgus macaques. Infect. Immun. *74*, 3790–3803. https://doi.org/10.1128/IAI.00064-06.

Lin, P.L., Plessner, H.L., Voitenok, N.N., and Flynn, J.L. (2007). Tumor necrosis factor and tuberculosis. J. Investig. Dermatol. Symp. Proc. *12*, 22–25. https://doi.org/10.1038/sj.jidsymp.5650027.

Lin, P.L., Rodgers, M., Smith, L., Bigbee, M., Myers, A., Bigbee, C., Chiosea, I., Capuano, S.V., Fuhrman, C., Klein, E., and Flynn, J.L. (2009). Quantitative comparison of active and latent tuberculosis in the cynomolgus macaque model. Infect. Immun. *77*, 4631–4642. https://doi.org/10.1128/IAI.00592-09.

Lin, P.L., Rutledge, T., Green, A.M., Bigbee, M., Fuhrman, C., Klein, E., and Flynn, J.L. (2012). CD4 T cell depletion exacerbates acute Mycobacterium tuberculosis while reactivation of latent infection is dependent on severity of tissue depletion in cynomolgus macaques. AIDS Res. Hum. Retroviruses *28*, 1693–1702. https://doi.org/10.1089/AID.2012.0028.

Lindestam Arlehamn, C.S., Gerasimova, A., Mele, F., Henderson, R., Swann, J., Greenbaum, J.A., Kim, Y., Sidney, J., James, E.A., Taplitz, R., et al. (2013). Memory T cells in latent Mycobacterium tuberculosis infection are directed against three antigenic islands and largely contained in a CXCR3+CCR6+ Th1 subset. PLoS Pathog *9*, e1003130. https://doi.org/10.1371/journal.ppat.1003130.

Lopez, D., Montoya, D., Ambrose, M., Lam, L., Briscoe, L., Adams, C., Modlin, R.L., and Pellegrini, M. (2017). SaVanT: a web-based tool for the sample-level visualization of molecular signatures in gene expression profiles. BMC Genomics *18*, 824. https://doi.org/10.1186/s12864-017-4167-7.

Lun, A.T.L., Riesenfeld, S., Andrews, T., Dao, T.P., Gomes, T.; Participants in the 1st Human Cell Atlas Jamboree, and Marioni, J.C. (2019). EmptyDrops: distinguishing cells from empty droplets in droplet-based single-cell RNA sequencing data. Genome Biol. *20*, 63. https://doi.org/10.1186/s13059-019-1662-y.

Lyadova, I.V., and Panteleev, A.V. (2015). Th1 and Th17 cells in tuberculosis: protection, pathology, and biomarkers. Mediators Inflamm. *2015*, 1–13. https://doi.org/10.1155/2015/854507.

Macosko, E.Z., Basu, A., Satija, R., Nemesh, J., Shekhar, K., Goldman, M., Tirosh, I., Bialas, A.R., Kamitaki, N., Martersteck, E.M., et al. (2015). Highly parallel genome-wide expression profiling of individual cells using nanoliter droplets. Cell *161*, 1202–1214. https://doi.org/10.1016/j.cell.2015.05.002.

Mahnke, Y.D., Brodie, T.M., Sallusto, F., Roederer, M., and Lugli, E. (2013). The who's who of T-cell differentiation: human memory T-cell subsets. Eur. J. Immunol. *43*, 2797–2809. https://doi.org/10.1002/eji.201343751.

Maiello, P., DiFazio, R.M., Cadena, A.M., Rodgers, M.A., Lin, P.L., Scanga, C.A., and Flynn, J.L. (2018). Rhesus macaques are more susceptible to progressive tuberculosis than cynomolgus macaques: a quantitative comparison. Infect. Immun *86*. https://doi.org/10.1128/IAI.00505-17.

Malherbe, S.T., Shenai, S., Ronacher, K., Loxton, A.G., Dolganov, G., Kriel, M., Van, T., Chen, R.Y., Warwick, J., Via, L.E., et al. (2016). Persisting positron emission tomography lesion activity and Mycobacterium tuberculosis mRNA after tuberculosis cure. Nat. Med. *22*, 1094–1100. https://doi.org/10.1038/nm.4177.

Martin, C.J., Cadena, A.M., Leung, V.W., Lin, P.L., Maiello, P., Hicks, N., Chase, M.R., Flynn, J.L., and Fortune, S.M. (2017). Digitally Barcoding Mycobacterium tuberculosis Reveals In Vivo Infection Dynamics in the Macaque Model of Tuberculosis. mBio *8*. e00312–17. https://doi.org/10.1128/mBio.00312-17.

Mateus, J., Lasso, P., Pavia, P., Rosas, F., Roa, N., Valencia-Hernandez, C.A., Gonzalez, J.M., Puerta, C.J., and Cuellar, A. (2015). Low frequency of circulating CD8+ T stem cell memory cells in chronic chagasic patients with severe forms of the disease. PLoS Negl. Trop. Dis *9*, e3432. https://doi.org/10.1371/journal.pntd.0003432.

Mattila, J.T., Diedrich, C.R., Lin, P.L., Phuah, J., and Flynn, J.L. (2011). Simian immunodeficiency virus-induced changes in T cell cytokine responses in cynomolgus macaques with latent Mycobacterium tuberculosis infection are associated with timing of reactivation. J. Immunol. *186*, 3527–3537. https://doi.org/10.4049/jimmunol.1003773.

Mattila, J.T., Maiello, P., Sun, T., Via, L.E., and Flynn, J.L. (2015). Granzyme B expressing neutrophils correlate with bacterial load in granulomas from Mycobacterium tuberculosis infected cynomolgus macaques. Cell Microbiol. *17*, 1085–1097. https://doi.org/10.1111/cmi.12428.

Mattila, J.T., Ojo, O.O., Kepka-Lenhart, D., Marino, S., Kim, J.H., Eum, S.Y., Via, L.E., Barry, C.E., 3rd, Klein, E., Kirschner, D.E., et al. (2013). Microenvironments in tuberculous granulomas are delineated by distinct populations of macrophage subsets and expression of nitric oxide synthase and arginase isoforms. J. Immunol. *191*, 773–784. https://doi.org/10.4049/jimmunol.1300113.

Mayer-Barber, K.D., Andrade, B.B., Oland, S.D., Amaral, E.P., Barber, D.L., Gonzales, J., Derrick, S.C., Shi, R., Kumar, N.P., Wei, W., et al. (2014). Host-directed therapy of tuberculosis based on interleukin-1 and type I interferon crosstalk. Nature *511*, 99–103. https://doi.org/10.1038/nature13489.

McCaffrey, E.F., Donato, M., Keren, L., Chen, Z., Delmastro, A., Fitzpatrick, M.B., Gupta, S., Greenwald, N.F., Baranski, A., Graf, W., et al. (2022). The immunoregulatory landscape of human tuberculosis granulomas. Nat. Immunol. *23*, 318–329. https://doi.org/10.1038/s41590-021-01121-x.

McGinnis, C.S., Murrow, L.M., and Gartner, Z.J. (2019). DoubletFinder: doublet detection in single-cell RNA sequencing data using artificial nearest neighbors. Cell Syst. *8*, 329–337.e4. https://doi.org/10.1016/j.cels.2019.03.003.

Meyer Zu Horste, G., Wu, C., Wang, C., Cong, L., Pawlak, M., Lee, Y., Elyaman, W., Xiao, S., Regev, A., and Kuchroo, V.K. (2016). RBPJ controls development of pathogenic Th17 cells by regulating IL-23 receptor expression. Cel Rep. *16*, 392–404. https://doi.org/10.1016/j.celrep.2016.05.088.

Millington, K.A., Innes, J.A., Hackforth, S., Hinks, T.S.C., Deeks, J.J., Dosanjh, D.P.S., Guyot-Revol, V., Gunatheesan, R., Klenerman, P., and Lalvani, A. (2007). Dynamic relationship between IFN-gamma and IL-2 profile of Mycobacterium tuberculosis-specific T cells and antigen load. J. Immunol. *178*, 5217–5226. https://doi.org/10.4049/jimmunol.178.8.5217.

Mishra, B.B., Rathinam, V.A.K., Martens, G.W., Martinot, A.J., Kornfeld, H., Fitzgerald, K.A., and Sassetti, C.M. (2013). Nitric oxide controls the immunopathology of tuberculosis by inhibiting NLRP3 inflammasome–dependent processing of IL-1β. Nat. Immunol. *14*, 52–60. https://doi.org/10.1038/ni.2474.

Mogilenko, D.A., Shpynov, O., Andhey, P.S., Arthur, L., Swain, A., Esaulova, E., Brioschi, S., Shchukina, I., Kerndl, M., Bambouskova, M., et al. (2021). Comprehensive profiling of an aging immune system reveals clonal GZMK(+) CD8(+) T cells as conserved hallmark of inflammaging. Immunity *54*, 99–115.e12. https://doi.org/10.1016/j.immuni.2020.11.005.

Mpande, C.A.M., Dintwe, O.B., Musvosvi, M., Mabwe, S., Bilek, N., Hatherill, M., Nemes, E., Scriba, T.J., and Team, S.C.I. (2018). Functional, antigen-specific stem cell memory (TSCM) CD4(+) T cells are induced by human Mycobacterium tuberculosis infection. Front. Immunol. *9*, 324. https://doi.org/10.3389/fimmu.2018.00324.

Munoz-Elias, E.J., Timm, J., Botha, T., Chan, W.T., Gomez, J.E., and McKinney, J.D. (2005). Replication dynamics of Mycobacterium tuberculosis in chronically infected mice. Infect. Immun. *73*, 546–551. https://doi.org/10.1128/IAI.73.1.546-551.2005.

Nakano, H., Lyons-Cohen, M., Nakano, K., Karcz, T., Thomas, S.Y., Whitehead, G., and Cook, D.N. (2019). Type 1 alveolar epithelial cell-derived chemokine CCL19 promotes airway inflammation. J. Immunol. *202*, 1722.

Newman, A.M., Liu, C.L., Green, M.R., Gentles, A.J., Feng, W., Xu, Y., Hoang, C.D., Diehn, M., and Alizadeh, A.A. (2015). Robust enumeration of cell subsets from tissue expression profiles. Nat. Methods *12*, 453–457. https://doi.org/10.1038/nmeth.3337.

Nikitina, I.Y., Panteleev, A.V., Kosmiadi, G.A., Serdyuk, Y.V., Nenasheva, T.A., Nikolaev, A.A., Gorelova, L.A., Radaeva, T.V., Kiseleva, Y.Y., Bozhenko, V.K., and Lyadova, I.V. (2018). Th1, Th17, and Th1Th17 lymphocytes during tuberculosis: Th1 lymphocytes predominate and appear as low-differentiated

CXCR3(+)CCR6(+) cells in the blood and highly differentiated CXCR3(+/-) CCR6(-) cells in the lungs. J. Immunol. *200*, 2090–2103. https://doi.org/10.4049/jimmunol.1701424.

O'Garra, A., Redford, P.S., McNab, F.W., Bloom, C.I., Wilkinson, R.J., and Berry, M.P. (2013). The immune response in tuberculosis. Annu. Rev. Immunol. *31*, 475–527. https://doi.org/10.1146/annurev-immunol-032712-095939.

Oehlers, S.H., Cronan, M.R., Scott, N.R., Thomas, M.I., Okuda, K.S., Walton, E.M., Beerman, R.W., Crosier, P.S., and Tobin, D.M. (2015). Interception of host angiogenic signalling limits mycobacterial growth. Nature *517*, 612–615. https://doi.org/10.1038/nature13967.

Ordovas-Montanes, J., Dwyer, D.F., Nyquist, S.K., Buchheit, K.M., Vukovic, M., Deb, C., Wadsworth, M.H., 2nd, Hughes, T.K., Kazer, S.W., Yoshimoto, E., et al. (2018). Allergic inflammatory memory in human respiratory epithelial progenitor cells. Nature *560*, 649–654. https://doi.org/10.1038/s41586-018-0449-8.

Padela, S., Yi, M., Cabacungan, J., Shek, S., Belcastro, R., Masood, A., Jankov, R.P., and Tanswell, A.K. (2008). A critical role for fibroblast growth factor-7 during early alveolar formation in the neonatal rat. Pediatr. Res. *63*, 232–238. https://doi.org/10.1203/pdr.0b013e31815f6e3a.

Pagan, A.J., and Ramakrishnan, L. (2014). Immunity and immunopathology in the tuberculous granuloma. Cold Spring Harb. Perspect. Med. *5*, a018499. https://doi.org/10.1101/cshperspect.a018499.

Phuah, J., Wong, E.A., Gideon, H.P., Maiello, P., Coleman, M.T., Hendricks, M.R., Ruden, R., Cirrincione, L.R., Chan, J., Lin, P.L., and Flynn, J.L. (2016). Effects of B Cell depletion on early Mycobacterium tuberculosis infection in cynomolgus macaques. Infect. Immun. *84*, 1301–1311. https://doi.org/10.1128/IAI.00083-16.

Phuah, J.Y., Mattila, J.T., Lin, P.L., and Flynn, J.L. (2012). Activated B cells in the granulomas of nonhuman primates infected with Mycobacterium tuberculosis. Am. J. Pathol. *181*, 508–514. https://doi.org/10.1016/j.ajpath.2012.05.009.

Polena, H., Boudou, F., Tilleul, S., Dubois-Colas, N., Lecointe, C., Rakotosamimanana, N., Pelizzola, M., Andriamandimby, S.F., Raharimanga, V., Charles, P., et al. (2016). Mycobacterium tuberculosis exploits the formation of new blood vessels for its dissemination. Sci. Rep. *6*, 33162. https://doi.org/10.1038/srep33162.

Potter, E.L., Gideon, H.P., Tkachev, V., Fabozzi, G., Chassiakos, A., Petrovas, C., Darrah, P.A., Lin, P.L., Foulds, K.E., Kean, L.S., et al. (2021). Measurement of leukocyte trafficking kinetics in macaques by serial intravascular staining. Sci. Transl. Med. *13*, eabb4582. https://doi.org/10.1126/scitranslmed.abb4582.

Prakadan, S.M., Shalek, A.K., and Weitz, D.A. (2017). Scaling by shrinking: empowering single-cell 'omics' with microfluidic devices. Nat. Rev. Genet. *18*, 345–361. https://doi.org/10.1038/nrg.2017.15.

Raphael, I., Nalawade, S., Eagar, T.N., and Forsthuber, T.G. (2015). T cell subsets and their signature cytokines in autoimmune and inflammatory diseases. Cytokine *74*, 5–17. https://doi.org/10.1016/j.cyto.2014.09.011.

Rath, J.A., Bajwa, G., Carreres, B., Hoyer, E., Gruber, I., Martinez-Paniagua, M.A., Yu, Y.R., Nouraee, N., Sadeghi, F., Wu, M., et al. (2020). Single-cell transcriptomics identifies multiple pathways underlying antitumor function of TCR- and CD8αβ-engineered human CD4 ⁺ T cells. Sci. Adv. *6*, eaaz7809. https://doi.org/10.1126/sciadv.aaz7809.

Reece, S.T., and Kaufmann, S.H. (2012). Floating between the poles of pathology and protection: can we pin down the granuloma in tuberculosis? Curr. Opin. Microbiol. *15*, 63–70. https://doi.org/10.1016/j.mib.2011.10.006.

Russell, D.G., Barry, C.E., 3rd, and Flynn, J.L. (2010). Tuberculosis: what we don't know can, and does, hurt us. Science *328*, 852–856. https://doi.org/10.1126/science.1184784.

Sakai, S., Kauffman, K.D., Sallin, M.A., Sharpe, A.H., Young, H.A., Ganusov, V.V., and Barber, D.L. (2016). CD4 T cell-derived IFN-gamma plays a minimal role in control of pulmonary Mycobacterium tuberculosis infection and must Be actively repressed by PD-1 to prevent lethal disease. PLoS Pathog *12*, e1005667. https://doi.org/10.1371/journal.ppat.1005667.

Schindelin, J., Arganda-Carreras, I., Frise, E., Kaynig, V., Longair, M., Pietzsch, T., Preibisch, S., Rueden, C., Saalfeld, S., Schmid, B., et al. (2012). Fiji: an open-source platform for biological-image analysis. Nat. Methods *9*, 676–682. https://doi.org/10.1038/nmeth.2019.

Scriba, T.J., Coussens, A.K., and Fletcher, H.A. (2017). Human immunology of tuberculosis. Microbiol. Spectr *5*. https://doi.org/10.1128/microbiolspec.TBTB2-0016-2016.

Shanmugasundaram, U., Bucsan, A.N., Ganatra, S.R., Ibegbu, C., Quezada, M., Blair, R.V., Alvarez, X., Velu, V., Kaushal, D., and Rengarajan, J. (2020). Pulmonary Mycobacterium tuberculosis control associates with CXCR3- and CCR6-expressing antigen-specific Th1 and Th17 cell recruitment. JCI Insight *5*, e137858. https://doi.org/10.1172/jci.insight.137858.

Smillie, C.S., Biton, M., Ordovas-Montanes, J., Sullivan, K.M., Burgin, G., Graham, D.B., Herbst, R.H., Rogel, N., Slyper, M., Waldman, J., et al. (2019). Intra- and inter-cellular rewiring of the human colon during ulcerative colitis. Cell *178*, 714–730.e22. https://doi.org/10.1016/j.cell.2019.06.029.

Stanley, S.A., Barczak, A.K., Silvis, M.R., Luo, S.S., Sogi, K., Vokes, M., Bray, M.-A., Carpenter, A.E., Moore, C.B., Siddiqi, N., et al. (2014). Identification of host-targeted small molecules that restrict intracellular Mycobacterium tuberculosis growth. PLos Pathog. *10*, e1003946. https://doi.org/10.1371/journal.ppat.1003946.

Szabo, P.A., Levitin, H.M., Miron, M., Snyder, M.E., Senda, T., Yuan, J., Cheng, Y.L., Bush, E.C., Dogra, P., Thapa, P., et al. (2019). Single-cell transcriptomics of human T cells reveals tissue and activation signatures in health and disease. Nat. Commun. *10*, 4706. https://doi.org/10.1038/s41467-019-12464-3.

Tabula Muris Consortium; Overall Coordination; Logistical Coordination; Organ Collection And Processing; Library Preparation And Sequencing; Computational Data Analysis; Cell Type Annotation; Writing Group; Supplemental Text Writing Group; Principal Investigators (2018). Single-cell transcriptomics of 20 mouse organs creates a Tabula Muris. Nature *562*, 367–372. https://doi.org/10.1038/s41586-018-0590-4.

Tobin, D.M., Roca, F.J., Oh, S.F., McFarland, R., Vickery, T.W., Ray, J.P., Ko, D.C., Zou, Y., Bang, N.D., Chau, T.T.H., et al. (2012). Host genotype-specific therapies can optimize the inflammatory response to mycobacterial infections. Cell *148*, 434–446. https://doi.org/10.1016/j.cell.2011.12.023.

Tobin, D.M., Vary, J.C., Ray, J.P., Walsh, G.S., Dunstan, S.J., Bang, N.D., Hagge, D.A., Khadge, S., King, M.-C., Hawn, T.R., et al. (2010). The lta4h locus modulates susceptibility to mycobacterial infection in zebrafish and humans. Cell *140*, 717–730. https://doi.org/10.1016/j.cell.2010.02.013.

Todryk, S.M. (2018). T cell memory to vaccination. Vaccines (Basel) *6*, 84. https://doi.org/10.3390/vaccines6040084.

Touzot, M., Grandclaudon, M., Cappuccio, A., Satoh, T., Martinez-Cingolani, C., Servant, N., Manel, N., and Soumelis, V. (2014). Combinatorial flexibility of cytokine function during human T helper cell differentiation. Nat. Commun. *5*, 3987. https://doi.org/10.1038/ncomms4987.

Ulrichs, T., and Kaufmann, S.H. (2006). insights into the function of granulomas in human tuberculosis. J. Pathol. *208*, 261–269. https://doi.org/10.1002/path.1906.

van Hamburg, J.P., and Tas, S.W. (2018). Molecular mechanisms underpinning T helper 17 cell heterogeneity and functions in rheumatoid arthritis. J. Autoimmun. *87*, 69–81. https://doi.org/10.1016/j.jaut.2017.12.006.

Varemo, L., Nielsen, J., and Nookaew, I. (2013). Enriching the gene set analysis of genome-wide data by incorporating directionality of gene expression and combining statistical hypotheses and methods. Nucleic Acids Res. *41*, 4378–4391. https://doi.org/10.1093/nar/gkt111.

Wacleche, V.S., Goulet, J.P., Gosselin, A., Monteiro, P., Soudeyns, H., Fromentin, R., Jenabian, M.A., Vartanian, S., Deeks, S.G., Chomont, N., et al. (2016). insights into the heterogeneity of Th17 subsets contributing to HIV-1 persistence during antiretroviral therapy. Retrovirology *13*, 59. https://doi.org/10.1186/s12977-016-0293-6.

White, A.G., Maiello, P., Coleman, M.T., Tomko, J.A., Frye, L.J., Scanga, C.A., Lin, P.L., and Flynn, J.L. (2017). Analysis of 18FDG PET/CT imaging as a tool for studying Mycobacterium tuberculosis infection and treatment in non-human primates. J. Vis. Exp *10*. https://doi.org/10.3791/56375.

WHO (2019). Global Tuberculosis Report (WHO).

Wijesundara, D.K., Tscharke, D.C., Jackson, R.J., and Ranasinghe, C. (2013). Reduced interleukin-4 receptor alpha expression on CD8+ T cells correlates with higher quality anti-viral immunity. PLoS One *8*, e55788. https://doi.org/10.1371/journal.pone.0055788.

Wohlfert, E.A., Grainger, J.R., Bouladoux, N., Konkel, J.E., Oldenhove, G., Ribeiro, C.H., Hall, J.A., Yagi, R., Naik, S., Bhairavabhotla, R., et al. (2011). GATA3 controls Foxp3+ regulatory T cell fate during inflammation in mice. J. Clin. Invest. *121*, 4503–4515. https://doi.org/10.1172/JCI57456.

Wolf, F.A., Angerer, P., and Theis, F.J. (2018). SCANPY: large-scale single-cell gene expression data analysis. Genome Biol. *19*, 15. https://doi.org/10.1186/s13059-017-1382-0.

Wong, E.A., Evans, S., Kraus, C.R., Engelman, K.D., Maiello, P., Flores, W.J., Cadena, A.M., Klein, E., Thomas, K., White, A.G., et al. (2020). IL-10 impairs local immune response in lung granulomas and lymph nodes during early Mycobacterium tuberculosis infection. J. Immunol. *204*, 644–659. https://doi.org/10.4049/jimmunol.1901211.

Wong, E.A., Joslyn, L., Grant, N.L., Klein, E., Lin, P.L., Kirschner, D.E., and Flynn, J.L. (2018). Low levels of T cell exhaustion in tuberculous lung granulomas. Infect. Immun *86*. https://doi.org/10.1128/IAI.00426-18.

Wu, C., Pot, C., Apetoh, L., Thalhamer, T., Zhu, B., Murugaiyan, G., Xiao, S., Lee, Y., Rangachari, M., Yosef, N., and Kuchroo, V.K. (2013). Metallothioneins negatively regulate IL-27-induced type 1 regulatory T-cell differentiation. Proc. Natl. Acad. Sci. U S A *110*, 7802–7807. https://doi.org/10.1073/pnas.1211776110.

Yang, X.O., Nurieva, R., Martinez, G.J., Kang, H.S., Chung, Y., Pappu, B.P., Shah, B., Chang, S.H., Schluns, K.S., Watowich, S.S., et al. (2008). Molecular antagonism and plasticity of regulatory and inflammatory T cell programs. Immunity *29*, 44–56. https://doi.org/10.1016/j.immuni.2008.05.007.

Yap, J., McCurdy, S., Alcala, M., Irei, J., Garo, J., Regan, W., Lee, B.-H., Kitamoto, S., and Boisvert, W.A. (2020). Expression of chitotriosidase in macrophages modulates atherosclerotic plaque formation in hyperlipidemic mice. Front. Physiol. *11*, 714. https://doi.org/10.3389/fphys.2020.00714.

Yosef, N., Shalek, A.K., Gaublomme, J.T., Jin, H., Lee, Y., Awasthi, A., Wu, C., Karwacz, K., Xiao, S., Jorgolli, M., et al. (2013). Dynamic regulatory network controlling TH17 cell differentiation. Nature *496*, 461–468. https://doi.org/10.1038/nature11981.

Young, M.D., and Behjati, S. (2018). SoupX removes ambient RNA contamination from droplet based single cell RNA sequencing data. Preprint at bioRxiv. https://doi.org/10.1101/303727.

Zhang, G., Zhou, B., Li, S., Yue, J., Yang, H., Wen, Y., Zhan, S., Wang, W., Liao, M., Zhang, M., et al. (2014). Allele-specific induction of IL-1β expression by C/EBPβ and PU.1 contributes to increased tuberculosis susceptibility. PLoS Pathog *10*, e1004426. https://doi.org/10.1371/journal.ppat.1004426.

Zilionis, R., Engblom, C., Pfirschke, C., Savova, V., Zemmour, D., Saatcioglu, H.D., Krishnan, I., Maroni, G., Meyerovitz, C.V., Kerwin, C.M., et al. (2019). Single-cell transcriptomics of human and mouse lung cancers reveals conserved myeloid populations across individuals and species. Immunity *50*, 1317–1334.e10. https://doi.org/10.1016/j.immuni.2019.03.009.

STAR★METHODS

KEY RESOURCES TABLE

REAGENT or RESOURCE	SOURCE	IDENTIFIER
Antibodies		
Mouse anti-human c-kit, clone CL1657	Novus Biologicals	Cat# NBP2-52975
Mouse anti-human tryptase, clone AA1	Abcam	Cat# ab2378; RRID: AB_303023
Mouse anti-human CD11c, clone 5D11	Leica Biosystems	Cat# CD11C-563-L-CE; RRID: AB_2750846
Rabbit anti-human CD20, polyclonal	ThermoFisher	Cat# RB-9013; RRID: AB_149767
Rabbit anti-human CD3, polyclonal	Dako Omnis	Cat# GA503
Donkey anti-rabbit IgG Alexa Fluor 647	Jackson ImmunoResearch Laboratories	Cat# 711-605-152; RRID: AB_2492288
Donkey anti-rabbit IgG Alexa Fluor 488	ThermoFisher	Cat# A32790; RRID: AB_2866495
Donkey anti-rabbit IgG Alexa Fluor 546	ThermoFisher	Cat# A10040; RRID: AB_2534016
Goat anti-mouse IgG1 Alexa Fluor 546	ThermoFisher	Cat# A21123; RRID: AB_2535765
Anti-rabbit IgG Alexa Fluor 488	ThermoFisher	Cat# Z25302; RRID: AB_2572214
Anti-rabbit IgG Alexa Fluor 546	ThermoFisher	Cat# Z25304; RRID: AB_2736947
Donkey anti-mouse IgG Alexa Fluor 488	ThermoFisher	Cat# A-21202; RRID: AB_141607
Mouse anti-human CD3, clone SP34-2	BD Biosciences	Cat# 551916; RRID: AB_394293
Mouse anti-human CD4, clone L200	BD Biosciences	Cat# 551980; RRID: AB_398521
Mouse anti-human CD8a, clone RPA-T8	BD Biosciences	Cat# 563823; RRID: AB_2687487
Mouse anti-human CD8b, clone 2ST8.5H7	BD Biosciences	Cat# 641058; RRID: AB_1645723
Mouse anti-human TCR gamma/delta, clone 5A6.E9	Invitrogen	Cat# TCR1061; RRID: AB_223500
Mouse anti-human CD16, clone 3G8	BD Biosciences	Cat# 556617; RRID: AB_396489
Mouse anti-human NKG2A, clone Z199	Beckman Coulter	Cat# A60797; RRID: AB_10643105
Mouse anti-human Granzyme B, clone GB11	BD Biosciences	Cat# 561998; RRID: AB_10894005
Mouse anti-human Granzyme A, clone CB9	BD Biosciences	Cat# 557449; RRID: AB_396712
Mouse anti-human Granzyme K, clone G3H69	BD Biosciences	Cat# 566655; RRID: AB_2869812
Bacterial and virus strains		
M. tuberculosis: Erdman strain	Flynn Lab	N/A
Biological samples		
Cynomolgus macaque granulomas	This study	N/A
Human granulomas	This study	N/A
Chemicals, peptides, and recombinant proteins		
2-mercaptoethanol	Sigma	Cat# M3148
Buffer RLT	QIAGEN	Cat# 79216
Buffer RLT Plus	QIAGEN	Cat# 1053393
Deoxynucleotide (dNTP) solution mix	NewEngland BioLabs	Cat# N0447L
Superase.In RNase Inhibitor	Thermo Fisher	Cat# AM2696
Maxima H minus reverse transcriptase	Fisher Scientific	Cat# EP0753
AMPure XP beads	Beckman Coulter	Cat# A63881
Guanidinium thiocyanate	Thermo Fisher	Cat# AM9422
N-Lauroylsarcosine sodium salt solution (Sarkosyl NL)	Sigma	Cat# L7414
Exonuclease I	New England BioLabs	Cat# M0293S
Klenow Fragment	New England BioLabs	Cat# M0212L
Polycarbonate membrane filters 62x22	Fisher Scientific/Sterlitech Corporation	Cat# NC1421644
MACOSKO-2011-10 mRNA Capture Beads	Fisher Scientific/ChemGenes	Cat# NC0927472

(*Continued on next page*)

Continued

REAGENT or RESOURCE	SOURCE	IDENTIFIER
Critical commercial assays		
Nextera XT DNA Library Preparation Kit	Illumina	Cat# FC-131-1096
Nextseq 500/550 High output v2.5 kit (75 cycles)	Illumina	Cat# 20024906
Kapa HiFi HotStart ReadyMix	Kapa Biosystems	Cat# KK2602
High Sensitivity D5000 ScreenTape	Agilent	Cat# 5067–5592
Qubit dsDNA High-Sensitivity kit	Thermo Fisher	Cat# Q32854
Rneasy Kit	Qiagen, Inc.	Cat# 74004
0.1mm Zirconia/Silica Beads	BioSpec Products	Cat# NC0362415
TaqMan Universal Master Mix II	Life Technologies	Cat# 4440043
Zombie NIR Fixable Viability Kit	BioLegend	Cat# 423105
Deposited data		
scRNA-seq data from 10-week p.i. granulomas	This study	Gene Expression Omnibus: GSE200151; https://singlecell.broadinstitute.org/single_cell/study/SCP257
scRNA-seq data from 4-week p.i. granulomas	This study	Gene Expression Omnibus: GSE200151; https://singlecell.broadinstitute.org/single_cell/study/SCP1749
Experimental models: Organisms/strains		
Cynomolgus macaques	Valley Biosystems	N/A
Oligonucleotides		
Seq-Well ISPCR: AAG CAG TGG TAT CAA CGC AGA GT	Integrated DNA Technologies	N/A
Custom Read 1 Primer: GCC TGT CCG CGG AAG CAG TGG TAT CAA CGC AGA GTA C	Integrated DNA Technologies	N/A
Seq-Well TSO: AAG CAG TGG TAT CAA CGC AGA GTG AAT rGrGrG	Integrated DNA Technologies	N/A
Seq-Well Custom P5-SMART PCR hybrid oligo: AAT GAT ACG GCG ACC ACC GAG ATC TAC ACG CCT GTC CGC GGA AGC AGT GGT ATC AAC GCA GAG TAC	Integrated DNA Technologies	N/A
Seq-Well dN-SMRT oligo: AAG CAG TGG TAT CAA CGC AGA GTG ANN NGG NNN B	Integrated DNA Technologies	N/A
Software and algorithms		
R project for statistical computing v4.1.2	R Core Team	https://www.r-project.org
R package – Seurat v4.0.2	GitHub	https://github.com/satijalab/seurat
R package – Circlize v0.4.8	CRAN	https://cran.r-project.org/web/packages/circlize/index.html
R package – data.table v1.12.0	GitHub	https://github.com/Rdatatable/data.table
R package – ggplot2 v3.2.1	CRAN	https://cran.r-project.org/web/packages/ggplot2/index.html
R package – ComplexHeatmap v2.7.3	Bioconductor	https://bioconductor.org/packages/ComplexHeatmap/
R package – dplyr v1.0.7	CRAN	https://cran.r-project.org/web/packages/dplyr/
GraphPad Prism v8 (GraphPad software, San Diego, CA), JMP Pro v12	Prism	https://www.graphpad.com/
JMP Pro v12	JMP	https://www.jmp.com/
FlowJo	FlowJo	https://www.flowjo.com/
DropSeqTools v1.12	Macosko et al., 2015	https://github.com/broadinstitute/Drop-seq
OsiriX DICOM	Pixmeo SARL	https://www.oxirix-viewer.com
NIS-Elements AR	Nikon	https://www.microscope.healthcare.nikon.com/products/software/nis-elements/nis-elements-advanced-research
SpectroFlo	Cytek	https://cytekbio.com/pages/spectro-flo

CellPress
OPEN ACCESS

RESOURCE AVAILABILITY

Lead contact

Further information and requests for resources, analytical code, and reagents should be directed to and will be fulfilled by the lead contact, Alex K. Shalek (shalek@mit.edu).

Materials availability

The study did not generate new unique reagents.

Data and code availability

- scRNA-seq data generated for this study is available at Gene Expression Omnibus. Accession numbers are listed in the key resources table. Processed data from granulomas sampled at 10 weeks p.i. can be accessed and visualized at https://singlecell.broadinstitute.org/single_cell/study/SCP257/cellular-ecology-of-m-tuberculosis-granulomas-10-week-dataset#/. Data from granulomas sampled at 4 weeks p.i. can be accessed and visualized at https://singlecell.broadinstitute.org/single_cell/study/SCP1749/cellular-ecology-of-m-tuberculosis-granulomas-4-week-dataset.
- All original code has been deposited to Zenodo at https://doi.org/10.5281/zenodo.6419143.
- Any additional information required to reanalyze the data reported in this paper is available from the lead contact upon request.

EXPERIMENTAL MODEL AND SUBJECT DETAILS

Research animals

Cynomolgus macaques (*Macaca fascicularis*), >4 years of age, (Valley Biosystems, Sacramento, CA) were housed within a Biosafety Level 3 (BSL-3) primate facility. Further information (including biological sex, number of granulomas, etc.) for each macaque involved in this study can be found in Table S1. Animals were infected with low dose (~10 colony-forming units (CFUs)) *M tuberculosis* (Erdman strain) via bronchoscopic instillation. Infection was confirmed by PET-CT scan at 4 weeks and monitored with clinical and radiographic examinations until 10 weeks p.i.

All experimental manipulations, protocols, and care of the animals were approved by the University of Pittsburgh School of Medicine Institutional Animal Care and Use Committee (IACUC). The protocol assurance number for our IACUC is D16-00118. Our specific protocol approval numbers for this project are 18124275 and IM-18124275-1. The IACUC adheres to national guidelines established in the Animal Welfare Act (7 U.S.C. Sections 2131 - 2159) and the Guide for the Care and Use of Laboratory Animals (8th Edition) as mandated by the U.S. Public Health Service Policy.

All macaques used in this study were housed at the University of Pittsburgh in rooms with autonomously controlled temperature, humidity, and lighting. Animals were singly housed in caging at least 2 square meters apart that allowed visual and tactile contact with neighboring conspecifics. The macaques were fed twice daily with biscuits formulated for nonhuman primates, supplemented at least 4 days/week with large pieces of fresh fruits or vegetables. Animals had access to water *ad libitum*. Because our macaques were singly housed due to the infectious nature of these studies, an enhanced enrichment plan was designed and overseen by our nonhuman primate enrichment specialist. This plan has three components. First, species-specific behaviors are encouraged. All animals have access to toys and other manipulata, some of which will be filled with food treats (e.g., frozen fruit, peanut butter, etc.). These are rotated on a regular basis. Puzzle feeders foraging boards, and cardboard tubes containing small food items also are placed in the cage to stimulate foraging behaviors. Adjustable mirrors accessible to the animals stimulate interaction between animals. Second, routine interaction between humans and macaques are encouraged. These interactions occur daily and consist mainly of small food objects offered as enrichment and adhere to established safety protocols. Animal caretakers are encouraged to interact with the animals (by talking or with facial expressions) while performing tasks in the housing area. Routine procedures (e.g. feeding, cage cleaning, etc) are done on a strict schedule to allow the animals to acclimate to a routine daily schedule. Third, all macaques are provided with a variety of visual and auditory stimulation. Housing areas contain either radios or TV/video equipment that play cartoons or other formats designed for children for at least 3 h each day. The videos and radios are rotated between animal rooms so that the same enrichment is not played repetitively for the same group of animals.

All animals are checked at least twice daily to assess appetite, attitude, activity level, hydration status, etc. Following *M. tuberculosis* infection, the animals are monitored closely for evidence of disease (e.g., anorexia, weight loss, tachypnea, dyspnea, coughing). Physical exams, including weights, are performed on a regular basis. Animals are sedated prior to all veterinary procedures (e.g. blood draws, etc.) using ketamine or other approved drugs. Regular PET/CT imaging is conducted on most of our macaques following infection and has proved very useful for monitoring disease progression. Our veterinary technicians monitor animals especially closely for any signs of pain or distress. If any are noted, appropriate supportive care (e.g. dietary supplementation, rehydration) and clinical treatments (analgesics) are given. Any animal considered to have advanced disease or intractable pain or distress from any cause is sedated with ketamine and then humanely euthanized using sodium pentobarbital.

METHOD DETAILS

Serial PET-CT Imaging

Animals underwent PET-CT scans after Mtb infection at 4 weeks, 8 weeks and pre necropsy (i.e. 10 weeks post-infection) as previously described (White et al., 2017). Briefly, animals were sedated, intubated and imaged by 2-deoxy-2-^{18}F-D-deoxyglucose (FDG) PET imaging (microPET Focus 220 preclinical PET scanner, Seimens Medical Solutions, USA, Malvern, PA) and Cretom CT scanner (Neurologica Corp, Danvers, MA, USA) within biosafety level 3 facility. The total lung FDG avidity was analyzed using Osirix viewer, an open-source PACS workstation and DICOM viewer (Pixmeo, Bernex, Switzerland). The whole lung was segmented on CT by using the growing region algorithm on the Osirix viewer to create a ROI of normal lung (Hounsfield units <200). The closing tool was used to include individual nodules and other pulmonary disease. The ROI was transferred to the co-registered PET scan and manually edited to ensure all pulmonary disease was included. Voxels outside the ROI were set to zero and voxels with an SUV greater than or equal to normal lung (SUV >2.3) were isolated. Finally, the "Export ROIs" plug-in was then used to export the data from these isolated ROIs to a spreadsheet where the total SUV per voxel were summed to represent the total lung FDG activity. Total FDG activity in lungs was used to estimate thoracic bacterial burden prior to reinfection (Figure 1C), as previously published (Coleman et al., 2014b; White et al., 2017). Granulomas were individually characterized by their date of establishment (scan date), size (mm), and relative metabolic activity as a proxy for inflammation ([^{18}F]-FDG standard uptake normalized to muscle [SUVR]) (Coleman et al., 2014b; White et al., 2017). Granulomas greater than 1mm are detected by CT scan.

Necropsy

Necropsy was performed as previously described (Gideon et al., 2015; Lin et al., 2009, 2013; Maiello et al., 2018). Briefly, an ^{18}F-FDG PET-CT scan was performed on every animal 1–3 days prior to necropsy to measure disease progression and identify individual granulomas. At necropsy, monkeys were maximally bled and humanely sacrificed using pentobarbital and phenytoin (Beuthanasia; Schering-Plough, Kenilworth, NJ). Individual granulomas previously identified by PET-CT and those that were not seen on imaging from lung and mediastinal lymph nodes were excised for histological analysis, bacterial burden, and other immunological studies. TB specific gross pathologic lesions and overall gross pathologic disease burden were quantified using a previously published method (Maiello et al., 2018). The size of each granuloma was measured by pre-necropsy scans and at necropsy. Granulomas were enzymatically dissociated using the gentleMACS dissociator system (Miltenyi Biotec Inc) to obtain a single suspension for enumerating bacterial burden and for single cell RNA-sequencing (scRNA-seq) on the Seq-Well platform.

Bacterial burden

200 μL of each granuloma homogenate were plated in serial dilutions onto 7H11 medium, and the CFU of *M. tuberculosis* growth were enumerated 21 days later to determine the number of bacilli in each granuloma (Gideon et al., 2015). As a quantitative measure of overall bacterial burden, a CFU score was derived from the summation of the log-transformed CFU/gram of each sample at the time of necropsy.

Chromosomal equivalents, CEQ

DNA extraction and qPCR were performed with modifications as described previously (Lin et al., 2014b). Briefly, frozen aliquots of homogenates were thawed and volumes recorded throughout the extraction process. Samples were transferred to tubes containing 150 μL of 0.1mm zirconia-silica beads (Biospec Products) before adding 600μL of Tris-EDTA buffer, pH 8.0. Three hundred microliters of phenol/chloroform/isoamyl alcohol (25:24:1, Sigma-Aldrich) at 70 °C were subsequently added and the samples incubated at room temperature for 10 min. The samples were then vortexed, the aqueous layer separated and supplemented with 50 μL 5M NaCl and a second phenol chloroform extraction performed on the extracted aqueous layer. DNA was precipitated with the addition of one volume of 100% isopropanol and one-tenth volume of 3M sodium acetate and incubating at −20 °C overnight. The DNA pellet was washed with 70% ethanol, dried and resuspended in nuclease-free water. Mtb genomes were then quantified using Taqman Universal Master Mix II (Life Technologies) and previously published sigF primer-probe combination (Lin et al., 2014b). Each sample was amplified in triplicate using an ABI Systems 7900HT machine. Chromosomal equivalents (CEQ) were quantified by comparing the samples with a standard curve derived from serial dilution of Mtb genomes prepared from liquid culture. Our detection limit for the standard curve was 10 copies per reaction. When we calculated the number of genomes for the whole granuloma, our detection limit was 1,000 copies per granuloma. Of the 26 granulomas analyzed, 2 granulomas failed at the CEQ quantification and they were eliminated from CEQ and CFU/CEQ analysis.

Immunohistochemistry analysis

Granulomas from macaques were harvested at 10 or 11 weeks post Mtb infection from other published (Phuah et al., 2016) and unpublished studies at the University of Pittsburgh. Following formalin fixation and paraffin embedding, 5 μm sections were placed on slides for staining. Slides were deparaffinized in xylenes, hydrated in a series of graded ethanol dips, and then antigen retrieval was performed by boiling the slides in a pressure cooker containing antigen retrieval citrate buffer for slides stained with c-kit and tryptase or Tris-EDTA buffer (Mattila et al., 2013) for slides stained with CD11c, CD20, and CD3. Sections were cooled to room temperature and washed with 1× PBS then stained overnight at 4 °C in a humidified chamber using anti-human c-kit, anti-mast cell tryptase antibodies, or rabbit-anti-CD3 and mouse anti-CD11c antibodies as previously described (Phuah et al., 2016). For the c-kit and tryptase

stained slides, the tissue sections were washed three times using 1× PBS and then incubated with anti-mouse IgG1 AF546 to label the anti-c-kit antibodies for 1 h at room temperature in a humidified chamber. Tryptase staining was performed overnight at 4 °C with anti-tryptase antibodies that were labeled with an Alexa Fluor 488 anti-rabbit IgG Zenon labeling kit. For the CD3, C11c, and CD20 stained sections, the CD3 and CD11c antibodies were labeled with donkey anti-rabbit IgG Alexa Fluor 647 and anti-mouse IgG Alexa Fluor 488-conjugated secondaries purchased from Jackson ImmunoResearch Laboratories (West Grove, PA) or ThermoFisher, respectively. After the secondary antibodies were removed with PBS washes, CD20 was stained with rabbit anti-CD20 that was labeled with Alexa Fluor 546 anti-rabbit IgG Zenon labeling kit. For both staining panels, the sections were washed again in 1× PBS and coverslips were applied using ProLong Gold Antifade Mountant with DAPI. For the slides stained with CD3, CD11c, and CD20, individual image channels were acquired with an Olympus FluoView 500 laser scanning confocal microscope (Olympus, Life Sciences Waltham, MA) maintained by the University of Pittsburgh's Center for Biologic Imaging and combined and pseudocolored with the FIJI build of ImageJ (Schindelin et al., 2012). Images of c-kit and tryptase-stained slides were acquired with a Nikon e1000 epifluorescence microscope (Nikon Instruments, Melville, NY) operated by the NIS-Elements AR software package (Nikon).

Human granulomas were identified from sections of lung tissue obtained at subjects undergoing partial lung resection for clinical indications at King Dinzulu Hospital and Inksosi Albert Luthili Central Hospital in Durban, South Africa. Gross pathology was assessed by Haematoxylin and Eosin (H&E) staining. Briefly, samples of lung were fixed in 10% neutral buffered formalin and processed routinely in a vacuum filtration processor using a xylene-free method with isopropanol as the main substitute fixative. Tissue sections were embedded in paraffin wax. Sections were cut at 4 μm using a microtome, heated at 56 °C for 15 min, dewaxed through two changes of xylene and rehydrated through descending grades of alcohol to water and stained with Haematoxylin & Eosin (H&E, 5 min incubation with each stain). Slides were dehydrated in ascending grades of alcohol, cleared in xylene, and mounted with a mixture of distyrene, plasticizer, and xylene (DPX). For immunohistochemistry, 4 μm sections and were mounted on charged slides and heated at 56 °C for 15 min. Mounted sections were dewaxed in xylene followed by rinsing in 100% ethanol and 1 change of SVR (95%). Slides were then washed under running water for 2 min followed by antigen retrieval via Heat Induced Epitope Retrieval (HIER) in Tris-sodium chloride (pH 6.0) for 30 min. Slides were then cooled for 15 min and rinsed under running water for 2 min. Endogenous peroxide activity was blocked using 3% hydrogen peroxide for 10 min at room temperature (RT). Slides were then washed in phosphate-buffered saline with 1% Tween (PBST) and blocked with protein block (Novolink) for 5 min at RT. Sections were incubated with primary antibodies for CD117 (A4502-CD117,c-kit, DAKO, 1:500), followed by washing and incubation with post primary (Novolink) for 30 min at RT. Slides were washed with PBST followed by incubation with the polymer (Novolink) for 30 min at RT. Slides were then washed and stained with DAB for 5 min, washed under running water and counterstained with hematoxylin for 2 min. Slides were rinsed under running water, blued in 3% ammoniated water for 30 s, washed under water, dehydrated and mounted in DPX.

Flow cytometry

Granulomas harvested from other Mtb infected NHPs were used in the flow cytometry analysis and processed as previously published (Gideon et al., 2015). Cells were counted and stained for viability using fixable viability dye (Zombie NIR, BioLegend) and other surface and intracellular markers using the standard protocols. Surface markers include: CD3 (SP34-2, BD), CD4 (L200, BD), CD8a (RPA-T8, BD), CD8b (2ST8.5H7, BD), TCR γδ (5A6.E9, Invitrogen), CD16 (3G8, BD), NKG2A (Z199, Beckman Coulter) and intracellular markers include: Granzyme B (GB11, BD), Granzyme A (CB9, BD) and Granzyme K (G3H69, BD). Samples were acquired on a Cytek Aurora spectral cytometer (5 laser configuration) and unmixed using SpectroFlo software (Cytek). Final analysis was performed in FlowJo (v10, FlowJo)

Single-cell RNA-sequencing (scRNA-seq)

High-throughput scRNA-seq was performed using the Seq-Well platform as previously described (Gierahn et al., 2017). Briefly, total cell counts from single-cell suspension of granuloma homogenate were enumerated and ~15,000–30,000 cells were applied to the surface of a Seq-Well device loaded with capture beads in the BSL-3 facility at University of Pittsburgh. Following cell loading, Seq-Well devices were reversibly sealed with a polycarbonate membrane and incubated at 37 °C for 30 min. After membrane sealing, Seq-Well devices were submerged in lysis buffer (5 M guanidine thiocyanate, 10 mM EDTA, 0.1% β-mercaptoethanol, 0.1% Sarkosyl) and rocked for 30 min. Following cell lysis, arrays were rocked for 40 min in 2 M NaCl to promote hybridization of mRNA to bead-bound capture oligos. Beads were removed from arrays by centrifugation and reverse transcription was performed at 52 °C for 2 h. Following reverse transcription, arrays were washed with TE-SDS (TE Buffer +0.1% SDS) and twice with TE-Tween (TE Buffer +0.01% Tween20). Following ExoI digestion, PCR amplification was performed to generate whole-transcriptome amplification (WTA) libraries. Specifically, a total of 2,000 beads were amplified in each PCR reaction using 16 cycles as previously described (Gierahn et al., 2017). Following PCR amplification, SPRI purification was performed at 0.6× and 0.8× volumetric ratios and eluted samples were quantified using a Qubit. Sequencing libraries were prepared by tagmentation of 800 pg of cDNA input using Illumina Nextera XT reagents. Tagmented libraries were purified using 0.6× and 0.8× volumetric SPRI ratios and final library concentrations were determined using a Qubit. Library size distributions were established using an Agilent TapeStation with D1000 High Sensitivity ScreenTapes (Agilent, Inc., USA).

Bulk RNA sequencing

Bulk RNA sequencing was performed using cells obtained from a total of 12 granulomas from a separate set of animals infected with Mtb for 10 weeks. Initially, granulomas were enzymatically dissociated and cells from each granuloma were placed in 100 uL of lysis

buffer. RNA was then extracted from whole lysates using RNeasy kits (Qiagen, Inc.) and combined with mRNA capture beads. Reverse transcription, whole transcriptome amplification, tagmentation and sequencing were performed as described above. Within each bulk RNA sequencing sample, expression values were summarized across bead barcodes to obtain an aggregate expression profile for each population.

Sequencing and alignment

Libraries for each sample were sequenced on a NextSeq550 or NovaSeq 6000 (Illumina Inc., Sunnyvale, CA, USA). For each library, 20 bases were sequenced in read 1, which contains information for cell barcode (12 bp) and unique molecular identifier (UMI, 8bp), while 50 bases were obtained for each read 2 sequence. Cell barcode and UMI tagging of transcript reads was performed using DropSeqTools v1.12 (Macosko et al., 2015). Barcode and UMI-tagged sequencing reads were aligned to the *Macaca fascicularis* v5 genome (https://useast.ensembl.org/Macaca_fascicularis/Info/Index) using the STAR aligner. Aligned reads were then collapsed by barcode and UMI sequences to generate digital gene expression matrices with 10,000 barcodes for each array.

QUANTIFICATION AND STATISTICAL ANALYSIS

Data processing and quality control

Initially, after examining a range of cell inclusion thresholds, a combined dataset of 169,830 barcodes was generated by applying a cutoff of 500 genes and 750 transcripts (UMIs). We visualized cells from each array using t-SNE across 30 principal components and performed Louvain clustering in Seurat. For many arrays, large clusters of cell barcodes were identified that were not marked by distinct cell-type defining gene expression. Instead, these cells were marked by distributed, low-level expression of genes presumed to originate from other cell types (e.g. *HBB* from erythrocytes, *JCHAIN* from plasma cells, and *CPA3* from mast cells). To understand the identity of these barcodes more fully, sequencing quality metrics were initially examined, and non-descript clusters did not significantly differ in the total number of aligned reads, detected genes, UMIs/cell, or mitochondrial percentage.

To more fully understand the identity of these clusters, multiple modeling approaches were pursued:

1. Initially, low-quality clusters were modeled as array-specific doublets. Here, models were constructed in which pseudo-doublets/multiplets (n = 2, 5, 10, 15, or 20 cells) were created from random sampling of the remaining cell type clusters. However, in these models, there was not significant overlap between the generated pseudo-multiplets and the clusters with non-distinct gene expression patterns.
2. Random cells were created by binomial sampling a pseudo-population average expression vector generated by summation of expression profiles across all cell type clusters not suspected to be derived from ambient contamination. In these models, direct overlap was not observed between the simulated mixed population and those clusters with non-distinct gene expression patterns.
3. Finally, we examined whether these clusters might represent deep sampling of ambient contamination or cellular debris by generating a "contamination" scoring scheme. First, to identify the clusters within each array, 30 principal components were calculated (this was observed to consistently capture the majority of variation in each array), and Louvain clustering (resolution = 1.25) was performed using all significant principal components (JackStraw Empirical P-value < 0.05). Next, within each array, cluster-specific "contamination" scores were generated that consisted of 3 components:
   a. **A measure of array-specific background contamination by cluster ("soup expression").** For each array, a background expression profile was generated based on low-UMI barcodes (See correction for residual background contamination below for full details). A set of "soup"-defining genes was identified at a range of thresholds for soup-defining gene expression (0.01, 0.005, 0.001, and 0.0005), a value that represents the proportional contribution of a given gene to the cumulative soup expression profile for each array. Array-specific, background-contamination scores were generated for the set of soup-defining transcripts using the AddModuleScore function in Seurat. Clusters with ambiguous/overlapping expression of lineage-defining gene expression signatures (Erythrocytes: *HBB*, Plasma cells: *JCHAIN*, Mast cells: *CPA3*, etc.) were observed to be significantly enriched for soup-defining gene expression. Finally, to calculate "contamination' scores, expression scores for soup genes at a threshold of 0.001 were generated to calculate the average soup-profile score for each cluster within each array.
   b. **An estimate of biological signal ("biological signal").** Here, the average log-fold change for the top 5 genes enriched within each cluster was calculated. For clusters dominated by ambient RNA, lower fold change enrichments for their biological signature genes were observed relative to clusters characterized by expression of canonical cluster-defining genes. In cases where the highest average log-fold change values within a cluster were below the "return threshold" in Seurat, we set the value to the default return threshold of 0.25.
   c. **A measure of co-expression of lineage-defining genes ("soup lineage coexpression").** 5 genes were manually selected that were recurrently over-represented in clusters suspected to arise from ambient contamination and cellular debris. Specifically, the following genes were selected: *HBB* (an erythrocyte-defining gene), *JCHAIN* (a plasma cell-defining gene), *COL3A1* (a fibroblast-defining gene), *SFTPC* (a type 2 pneumocyte-defining gene), and *CPA3* (a mast cell-defining gene). For each cell barcode, the number of these five genes with non-zero expression was calculated as a measure of lineage-defining co-expression. Within each cluster, the average co-expression of these genes was calculated and one was subtracted from this average to allow for endogenous expression of 1 lineage-defining gene. This parameter was

specifically added to avoid exclusion of *bona fide* cell clusters with high-background contamination (presumably due to low endogenous RNA content) and low biological signal (e.g., naive T cells). Here, cell populations that scored high for markers of a single lineage yet had higher soup-expression scores presented with lower rates of co-expression of these soup and lineage defining transcripts relative to clusters which did not, likely representing ambient RNA and debris.

Using these three values, cluster-specific background "contamination" scores were calculated for each array in 2 ways:

$$Contamination\ Score\ 1 = \frac{(Soup\ Expression)\ x\ (Soup\ Lineage\ Coexpression)}{Biological\ Signal}$$

$$Contamination\ Score\ 2 = \frac{(Scaled - Soup\ Expression)\ x\ (Soup\ Lineage\ Coexpression)}{Biological\ Signal}$$

These two "contamination" scores quantify both the (1) absolute and (2) relative soup-profile contamination in subsequent cluster classification.

Next, for each array, clustering was performed to identify clusters with array-specific ambient contamination and debris. Specifically, hierarchical clustering was performed using a total of 7 variables to identify clusters defined by ambient contamination: the 2 contamination scores (shown above), three scaled soup scores (soup gene thresholds: 0.01, 0.05 and 0.001), the average log-fold change for the top 5 cluster genes, and soup/lineage gene co-expression. For each array, the hierarchical clustering tree was cut at the first branch point to identify clusters with a signature of ambient contamination. In total, 41 array-specific clusters, comprising 56,590 barcodes from 21 out of 32 total arrays, were identified as characterized by ambient RNA contamination and cellular debris and removed them in all subsequent analyses.

Correction for residual background contamination

After removal of cell barcodes that were derived from background contamination and extracellular debris, additional correction for ambient RNA contamination was performed among remaining cell barcodes on an array-by-array basis. Among filtered cell barcodes, array-specific, ambient RNA contamination was observed to be marked by ectopic expression of cell-type defining genes (e.g., widespread expression of *JCHAIN*, *HBB*, and *CPA3*, etc.). Specifically, this contamination was observed to vary in relation to the overall distribution of cell types recovered from each array. To correct for residual ambient contamination within each array, SoupX (Young and Behjati, 2018) was used to: (1) generate array-specific profiles of background contamination, (2) estimate per-cell contamination fractions, and (3) generate corrected background-corrected UMI counts matrices. To generate background expression profiles, counts matrices containing up to 50,000 barcodes were generated to assemble a collection of low-UMI cell barcodes that presumably represent extracellular mRNA. For each array, a UMI threshold for background expression was determined using EmptyDrops (Lun et al., 2019) to estimate the likelihood distribution that low-UMI barcodes represent cells rather than ambient contamination. Using an array-specific UMI-threshold (Range: 20–100 UMIs), a composite background profile was created for each array. To estimate the per-cell contamination fraction, a set of lineage-defining genes was first identified with bimodal expression patterns across cells (i.e., lineage defining genes with leaky expression). For each array, this set of soup-defining, lineage genes was used to estimate contamination fraction for cell types with known endogenous expression. Finally, the composite soup profile was subtracted from each the transcriptional profile of each cell based on the estimated contamination fraction. For each array, individual transcripts most likely to be contamination were removed from each single-cell based on the estimated contamination fraction. Specifically, individual transcripts were sequentially removed from each single-cell transcriptome until the probability of subsequent transcripts being soup-derived was less than 0.5 to generate a background-corrected counts matrix for each array.

Separation of doublets

Within each array, doublet identification and separation were performed using DoubletFinder. To account for differences in cell loading densities and expected cell doublet frequencies, array-specific estimates of the expected number of doublets were generated (Table S1). For example, for a total of 20,000 cells applied to a Seq-Well device containing 85,000 wells (lambda = 20,000), an expected doublet rate of >2.37% (since not all of the array's surface area contains wells) was calculated. For each array, pseudo-doublets were generated using DoubletFinder (McGinnis et al., 2019). Here, the pK parameter estimate was separately optimized for each array by performing a parameter sweep in which we selected the pK value with the maximum bimodality coefficient, while a pN = 0.25 was maintained across all arrays based on published recommendations (McGinnis et al., 2019). Cells were identified as doublets based on their rank order in the distribution of the proportion of artificial nearest neighbors (pANN). Specifically, the pANN value for the cell at the expected doublet percentile was identified and the corresponding pANN value was used as a threshold to remove additional cells in the event of ties. In total, we excluded 3,656 cells as doublets.

Integrated cell type classification

Following the aforementioned quality filtering, a combined dataset of 109,584 cells was used in downstream analysis (Table S1). An initial dimensionality reduction was performed on these cells by selecting 1580 variable genes, performing principal component analysis (PCA), UMAP dimensionality reduction and Louvain clustering using Scanpy (Wolf et al., 2018). To identify broad cell types,

we examined cluster assignments at multiple clustering resolutions (Resolutions: 0.5 to 2.25). We selected a cluster resolution of 1.00 because this was the resolution beyond which branching did not result in discovery of clusters that represent distinct cell lineages (e.g., division of Type 1 and Type 2 pneumocytes) (Table S2). To define major cell populations, extensive comparisons to existing signatures of lung parenchyma and immune cell populations were performed using data from the Tabula Muris (Tabula Muris Consortium et al., 2018) and Mouse Cell Atlas (Han et al., 2018) studies. Specifically, lung scRNA-seq data from both studies were collected and used to calculate enriched gene expression signatures for each lung cell type cluster using a Wilcox rank-sum test. For each cluster, the top 20 genes (Table S2) were selected as a cluster-specific expression signature and then used them to score all cells in the granuloma dataset. The average signature score within each cluster was calculated and the distribution of signature score was examined within each granuloma cell type, and significance was determined via permutation testing.

Cell type assignment of proliferating cells

Among our top-level clusters was one defined by markers of cellular proliferation (*MKI67*, *TOP2A*, and *CDK1*). To identify the underlying cell type identity for these cells, a separate dimensionality reduction and clustering was performed among 3,123 cells defined by this proliferation signature. UMAP dimensionality reduction and Louvain clustering was running at multiple clustering resolutions (0.4-0.8), and a resolution of 0.70 was selected as the value beyond which no additional major cell type clusters were observed (Figure S3E). For each of the major cell types identified in the global clustering analysis, we generated a gene signature using the top 20 enriched genes and scored the proliferating cells clusters using the AddModuleScore function in Seurat. We then examined the distribution of cell-type signature scores across each of the sub-clusters of proliferating cells and re-assigned clusters based on enrichment of lineage-specific gene expression. Here, we assessed the significance of the cluster scores using a permutation test. More specifically, 1,000 permutations were performed in which the proliferating clusters were down-sampled to have the same number of cells. Cluster assignments of the cells were randomized and the average generic cell type signature score was calculated for each randomized cluster. The significance of a cell type score for each proliferating cluster was determined by comparing the observed average signature score to the random null distribution. Through this approach, distinct clusters of proliferating B cells, macrophages, neutrophils, plasma cells, and T cells were identified and re-assigned to their respective cell types.

Filtering of soup-defining transcripts

To avoid artifacts from ambient RNA contamination and cellular debris in sub-clustering of T cells and macrophages, genes that were observed to be soup-defining for any array were excluded. Specifically, a set of 210 soup-defining genes was identified that comprised 0.001 of total soup expression in any array. The threshold of 0.001 was selected to maximize the cumulative fraction of soup expression with the least number of genes to avoid removing underlying biology. Here, this threshold value represents cumulative fraction of soup expression accounted for by a given gene for each array. In a further effort to avoid removing cell type specific biology, any genes with average log-fold changes greater than 1.00 in T cells and macrophages compared to all other generic cell types were retained. In total, 204 and 180 genes were removed prior to sub-clustering analysis of T cells and macrophages, respectively.

Sub-clustering of granuloma unified T and NK cells

Across the complete set of 44,766 T and NK cells, Louvain clustering was initially performed at a range of resolution of values (0.30–0.75) to examine the relationships between cluster membership. In this analysis, a cluster was observed to be defined by persistent expression of contaminating transcripts derived from macrophage and mast cells (Cluster 4 - Louvain Resolution 0.60). To confirm that these cells did not represent persistent doublets, all T cells were scored by expression of the top 20 cluster defining T cells and similar signature scores between the contaminated cell population were observed. Additional sub-clustering within the "contaminated" T cell cluster was performed to understand whether residual contamination obscured additional T cell biology; this failed to reveal additional T cell clusters not identified among the remaining non-contaminated populations. Since this contamination cluster was not observed to obscure a novel T cell phenotype, this population was excluded from downstream analysis (including compositional analyses associating cell type/group abundances with bacterial burden). Following removal of the cluster of T cells defined by residual contamination, dimensionality reduction and clustering at multiple clustering resolutions (Louvain resolution: 0.25–0.75) were performed. In this final analysis, a total of 12 T cell populations were identified at a clustering resolution of 0.75. Finally, additional sub-clustering was performed within the population of 2,377 $\gamma\delta$ and cytotoxic T cells, including dimensionality reduction and clustering at multiple resolutions (0.30–0.75). Here, 2 primary populations of cells were identified: sub-cluster 2, a population of cytotoxic cells enriched for expression of *TRDC* and sub-cluster 3, a population of XCL1+ NK cells. Differential expression analysis was performed to determine differences in gene expression between these clusters upon which the classification of these cells was based.

Additional sub-clustering analysis was performed within the T1-T17 population through repeated variable gene identification, dimensionality reduction and Louvain clustering (Resolution = 0.55), and 4 distinct sub-populations were discovered. Differential expression analysis was performed within the 9,234 T1-T17 cells using a Wilcoxon test in Seurat to identify sub-cluster defining gene signatures.

Annotation of T /NK subclusters

T cell populations were classified using a combination of manual curation and comparison to literature-derived sequences. Granuloma T cell populations were compared to publicly available T cell population and scRNA-seq signatures. Specifically, comparisons were performed in the following ways:

1. For each T cell cluster, cluster-defining genes were compared to publicly available databases of immune signatures, including IPA, GeneGO, MSigDb (Liberzon et al., 2011) and SaVant (Lopez et al., 2017). This was performed by comparing the set of T cell cluster-defining genes (Adjusted p value < 0.001 and log-FC > 0.2) to the signatures in GSEA and the SaVant data using Piano (Lopez et al., 2017; Varemo et al., 2013). Specifically, significance was assessed using a hypergeometic test to examine the likelihood of the observed frequency of enriched genes. Among cluster-defining genes for each T/NK cell sub-cluster, comparisons were performed within each GSEA collection C1-7 (https://www.gsea-msigdb.org/gsea/msigdb/collections.jsp) and to the SaVant database. Expression signatures were also compared to MSigDB signatures using GSEA. Here, pseudo-bulk expression signatures were generated for each T/NK sub-population as the average gene expression across all cells within each cluster. These average expression values were used to perform GSEA for each cluster in which the expression values were compared to all other clusters using 1,000 permutations.
2. Each T cell cluster was compared to literature-derived signatures of T cells from another scRNA-seq study. Here, cell signature scores were generated in Seurat using the AddModuleScore function using gene expression signatures obtained from human lung cancer (Guo et al., 2018). To determine the significance of these score, 1,000 permutations were performed in which T cell cluster identity was randomly re-assigned to generate a null distribution of module scores.
3. Finally, extensive manual curation was performed based on literature evidence. For each cell population, an extensive literature search was performed to support classification of T cell sub-populations based on patterns of enriched gene expression. For example, regulatory T cells were identified on the basis of expression of known regulatory T cell markers (*FOXP3, IKZF1*, and *TNFSF18/GITR*). However, in many cases, surface markers used to define canonical T cell populations were not detected in the scRNA-seq data.

Next, expression of *TRAC* and *TRBC* or *TRDC* was evaluated within T cells in the scRNA-seq data and the frequency of cells expressing either *TRAC/TRBC* (yellow) or *TRDC* (green) within each of the 13 clusters was calculated. While *TRAC/TRBC* expression was observed in all 13 subclusters, *TRDC* expression was observed mainly in subclusters 1-3 compared to subclusters 4–13. Finally, cluster-specific expression of *CD4* and *CD8A* and *CD8B* were examined as the proportion of cells with non-zero expression of *CD4*, *CD8A/B* or *CD4&CD8 (A/B)*.

Sub-clustering of granuloma macrophages

Across 27,670 macrophages, dimensionality reduction and Louvain clustering at multiple clustering resolutions was performed. In initial clustering, a cluster defined by contaminating transcripts derived from other cell types (including mast cells (*KIT* and *CLU*), T cells (*CD3D*), and plasma cells (*JCHAIN*)) and soup-defining gene expression was identified. By comparing the distribution of macrophage-defining gene expression in this cluster to other clusters, this cluster was observed to have enriched signature scores relative to other clusters. The enrichment of macrophage expression signatures was examined to determine the population of macrophages that have a core macrophage expression program. While this population of macrophages exhibits primarily soup-defining gene expression, this cluster was not excluded due to the possibility that this represents an efferocytotic macrophage population.

Classification of macrophage populations

Identities of the macrophage clusters were established through a combination of manual curation and comparison to published gene expression signatures from both population and scRNA-seq studies. More specifically:

1. For each macrophage cluster, similar comparison to databases of immune signatures including MSigDb and SaVanT were performed (See ***Identification of T cell Populations***).
2. A series of gene expression signatures were generated from published scRNA-Seq studies of macrophage states. For example, a recently published atlas of myeloid states in lung (Zilionis et al., 2019) was used to score granuloma macrophages. Further, a list of myeloid expression signatures was generated using lung myeloid cells from the Mouse Cell Atlas (Han et al., 2018). For each study, signatures for the top 20 cluster-defining genes were selected to generate gene expression signatures (Table S3). Signature scores were generated for each cell using the AddModuleScore function in Seurat.
3. Finally, in cases where an existing description of a macrophage population was not discovered, extensive literature searches were performed to contextualize possible identities of macrophage populations.

Deconvolution of bulk RNA-sequencing data

Population deconvolution was performed using CiberSort (Newman et al., 2015) using reference populations generated from random sampling of a quarter of the single cells within each of the 13 generic cell types identified in our single-cell analysis.

Co-variation in granuloma composition

We calculated correlations in cell-type proportions to identify underlying structure in the co-occurrence of cell types across all granulomas. Specifically, we calculated Pearson correlation coefficients for all pair-wise cell-type combinations (N.B., we also performed each analysis using Spearman correlation coefficients and obtained similar results). For each pairwise combination of cell types, we calculated permutation p values by randomly re-assigning cell type labels to generate a set of background correlation values (Table S5).

We then performed hierarchical clustering to identify clusters of correlated cell-types across granulomas, calculating the proportional composition of correlated cell-type clusters within each lesion. For each of the 5 clusters identified through hierarchical clustering, we calculated permutation p values to examine average correlation values. To understand the relationship between identified cell-type clusters and granuloma-level bacterial burden, we examined the abundance of correlated cell types by grouping lesions by timing of granuloma formation.

Cell-communication analysis

To examine cell-cell interactions, we first generated a curated list of receptor-ligand pairs through a combination of publicly-available databases and literature review. Within each granuloma, we generated edge weights between cell types for a given receptor ligand pair by multiplying the average receptor expression in Cell Type 1 by the average ligand expression in Cell Type 2. Edge weights were constructed for all receptor-ligand pairs and pairwise-cell type combinations within granulomas individually. Within each granuloma, we performed a total of 1,000 permutations for each receptor-ligand pair in which cell-type identifiers were randomly resorted and the resulting edge weight was recorded. For each receptor-ligand pair, the significance of the observed value was calculated from a z-score comparison of the observed value relative the permuted values.

We further performed adjustment of receptor-ligand edge weights at multiple levels. (1) To account for differences in the relative abundance of 'sender' cell types, we multiplied receptor-ligand edge weights by the proportion of all 'sender' cells within a granuloma. In effect, this generates a pool of 'sender' cell derived ligand that is available to act upon cell types bearing appropriate receptors. (2) To identify the most likely receiver cells, we weighted receptor-ligand edge-weights by the proportion of total receptor expression within the receiving cell subset cluster relative to the average receptor expression across all cells in the granuloma. In this scheme, receptors with more uniform expression across the entire granuloma will be down-weighted to reflect non-autonomous sinks of extracellular ligands, while receptors predominantly expressed by a single cell subset will be up-weighted. (3) Finally, we adjusted receptor-ligand edge weights to account for the percent of cells within the receiver cell subset expressing a given receptor by multiplying our receptor-ligand edge weights by the proportion of all 'receiver' cells expressing the receptor within the receiver cell subset.

To identify axes of intercellular communication with differential weights across granulomas, we performed student's t-tests of receptor-ligand edge weights between (A) high-burden and low-burden lesions, and (B) original and late-blooming lesions. We filtered results based on the following criteria: (1) the average permutation p values for the receptor-ligand pair within high or low-burden lesions <0.05, (2) p value from Student's *t* test in (A) or (B) above <0.05. The "dplyr" package in R was used to filter the resulting cell-cell interaction database to count significant interactions across cell type groups and granuloma burdens, identify cell type groups contributing to the top 10% of ligands most strengthened in either high or low burden granulomas, identify ligands most associated with high or low burden granulomas, and identify cell type specificity of these ligands. The "circlize" package in R was used to generate circus plots of the topology of signaling networks across high and low burden granulomas.

Statistical methods

Non-parametric Spearman's rho with Benjamini-Hochberg multiple testing correction was calculated for correlation analysis for evaluating the degree of relationship between cellular abundance and bacterial burden. As a complementary analysis approach that accounts for the inter-dependent nature of compositional data (i.e., where changes in counts of one cell type necessarily affect proportions of all other cell type, Dirichlet regression analysis was conducted to evaluate relationships between cell type abundances with bacterial burden; cell types were prioritized based on concordance between these two statistical testing frameworks. Non parametric t test or Mann-Whitney U test was used when comparing two groups. p values, or where appropriate adjusted or permutation p values, ≤ 0.05 were considered significant. Statistical analysis was performed using GraphPad Prism v8 (GraphPad software, San Diego, CA), JMP Pro v12 and R base statistics.

Immunity, Volume *55*

Supplemental information

Multimodal profiling of lung granulomas in macaques reveals cellular correlates of tuberculosis control

Hannah P. Gideon, Travis K. Hughes, Constantine N. Tzouanas, Marc H. Wadsworth II, Ang Andy Tu, Todd M. Gierahn, Joshua M. Peters, Forrest F. Hopkins, Jun-Rong Wei, Conner Kummerlowe, Nicole L. Grant, Kievershen Nargan, Jia Yao Phuah, H. Jacob Borish, Pauline Maiello, Alexander G. White, Caylin G. Winchell, Sarah K. Nyquist, Sharie Keanne C. Ganchua, Amy Myers, Kush V. Patel, Cassaundra L. Ameel, Catherine T. Cochran, Samira Ibrahim, Jaime A. Tomko, Lonnie James Frye, Jacob M. Rosenberg, Angela Shih, Michael Chao, Edwin Klein, Charles A. Scanga, Jose Ordovas-Montanes, Bonnie Berger, Joshua T. Mattila, Rajhmun Madansein, J. Christopher Love, Philana Ling Lin, Alasdair Leslie, Samuel M. Behar, Bryan Bryson, JoAnne L. Flynn, Sarah M. Fortune, and Alex K. Shalek

Fig S1

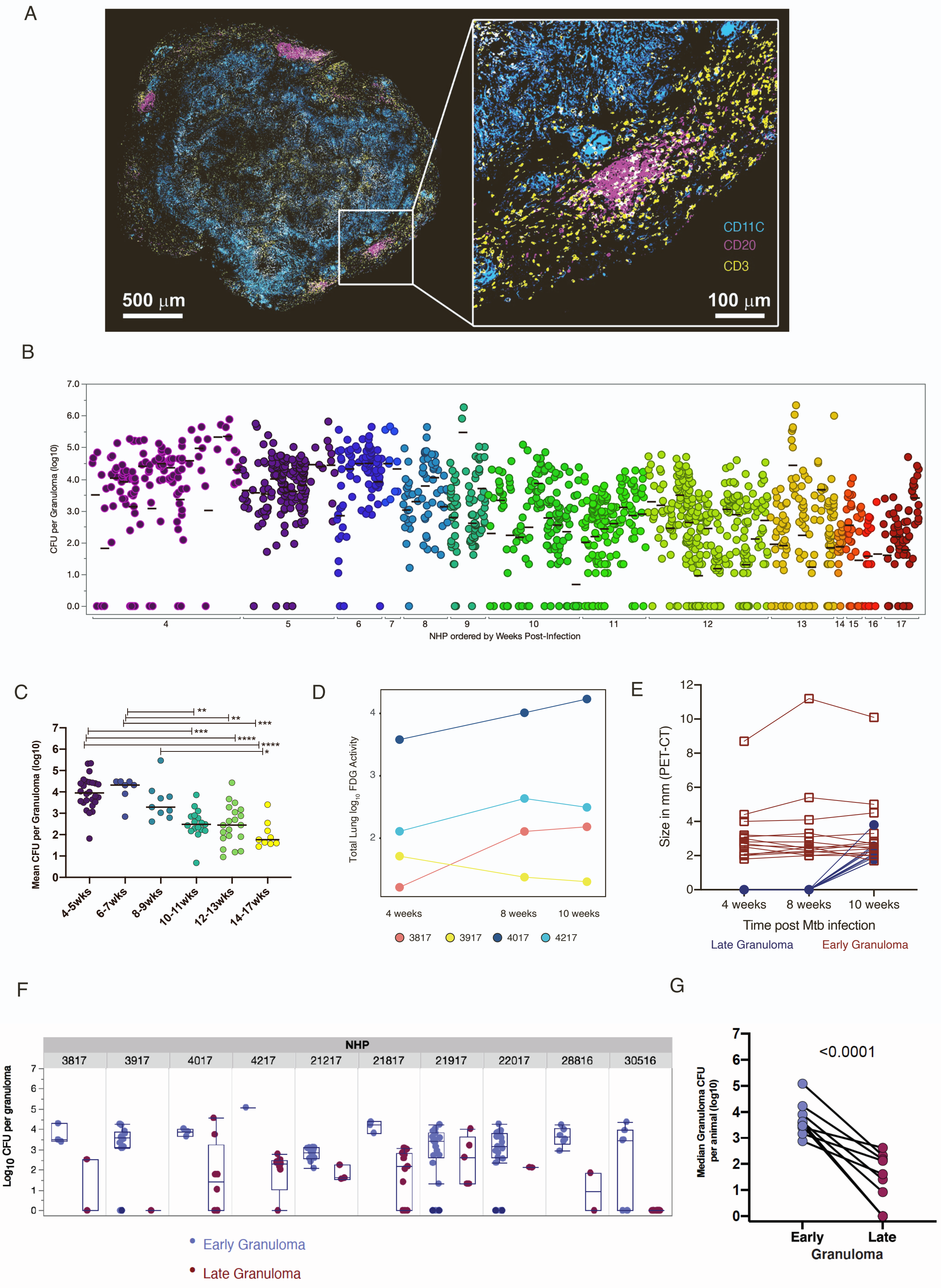
A
500 μm
100 μm
CD11C
CD20
CD3
B
CFU per Granuloma (log10)
7.0
6.0
5.0
4.0
3.0
2.0
1.0
0.0
4
5
6
7
8
9
10
11
12
13
14
15
16
17
NHP ordered by Weeks Post-Infection
C
Mean CFU per Granuloma (log10)
**
**
***
***
****
****
*
4-5wks
6-7wks
8-9wks
10-11wks
12-13wks
14-17wks
D
Total Lung log10 FDG Activity
4 weeks
8 weeks
10 weeks
3817
3917
4017
4217
E
Size in mm (PET-CT)
4 weeks
8 weeks
10 weeks
Time post Mtb infection
Late Granuloma
Early Granuloma
F
NHP
3817
3917
4017
4217
21217
21817
21917
22017
28816
30516
Log10 CFU per granuloma
Early Granuloma
Late Granuloma
G
Median Granuloma CFU per animal (log10)
<0.0001
Early
Late
Granuloma

Figure S1. Characterization of granuloma architecture and bacterial burden dynamics (related to Fig 1)

(**A**) Architecture of macaque TB lung granuloma, where lymphocytes and macrophages are present in distinct regions. Immunohistochemistry and confocal microscopy were performed on a granuloma from an animal at 11 weeks post-Mtb infection to visualize localization of CD11c+ macrophages (cyan), CD3+ T cells (yellow), and CD20+ B cells (magenta). (**B**) Each column depicts the CFU for all granulomas of an individual macaque (N=88 macaques), ranging from 4 weeks to 17 weeks post-infection. Each dot represents a granuloma. Lines are at means (per animal) and different colors represent weeks post-infection. (**C**) CFU per granuloma decreases significantly starting at 10-11 weeks post-infection. Each dot represents the mean CFU per granuloma of an individual animal, with the x-axis indicating weeks post-infection at which necropsy was performed. Lines are at medians. Differences between time points were tested using Kruskal-Wallis test with Dunn's multiple comparison adjustment. (* $p < 0.05$, ** $p < 0.01$, *** $p < 0.001$, **** $p < 0.0001$.) (**D**) Total lung FDG activity (in log scale) measured by PET scans of each animal at 4, 8 and 10-weeks post-Mtb infection showing trajectories of lung inflammation. (**E**) Size of each granuloma measured by CT scans at 4, 8 and 10 weeks post-mtb infection. Early granulomas are those identified at 4 weeks post infection (in maroon) and late granulomas are those identified at 10 weeks post infection (in dark blue). (**F**) CFU per granuloma is shown for early detection (blue) and late detection (red) within each animal. Box plots lines represent the median, IQR and range Each dot represents a granuloma. (**G**) CFU is significantly lower in new granulomas within animals. Each dot (and line) represents the median CFU per granuloma of each animal. Statistics: paired t-test

Fig S2

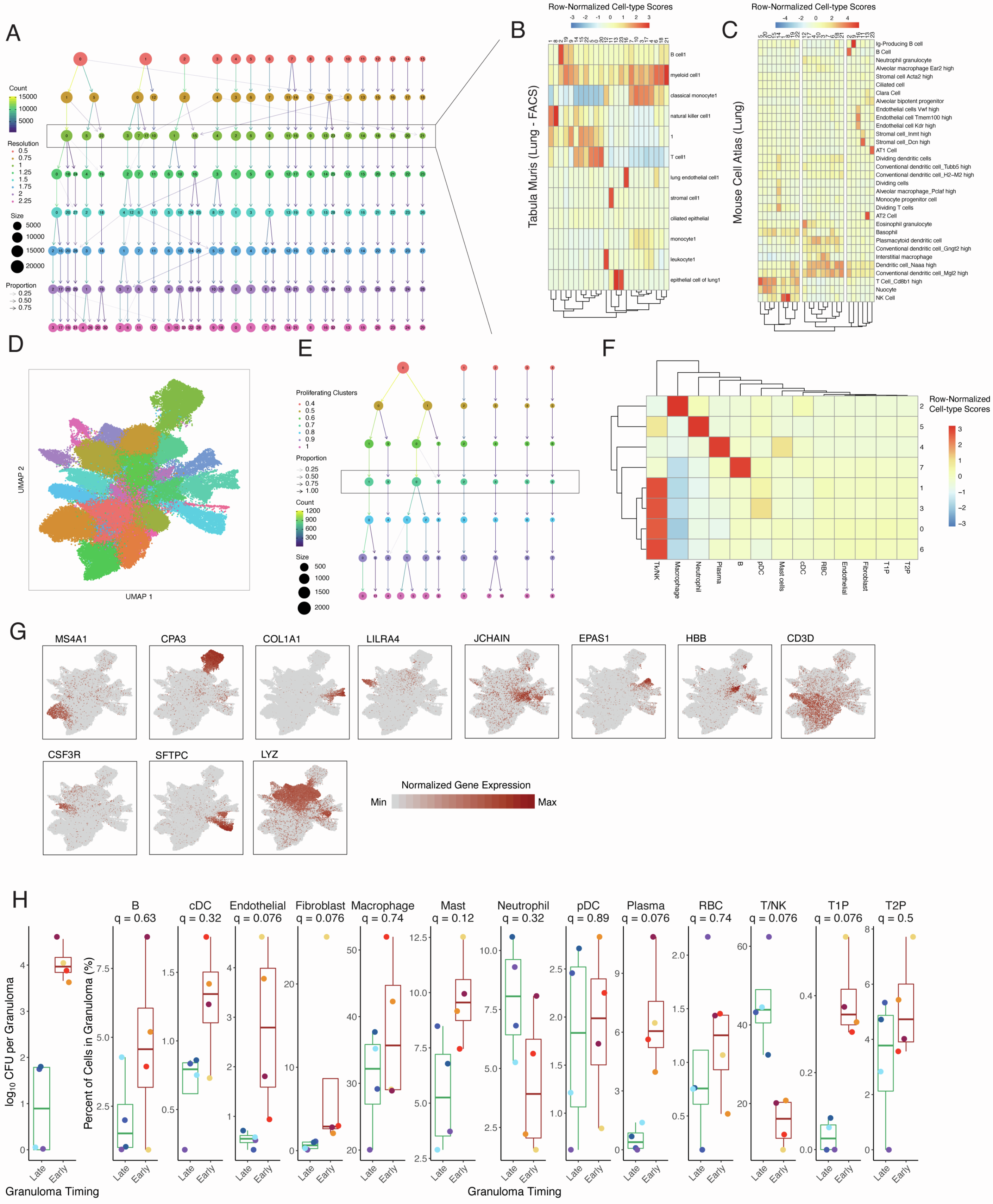
A
B
C
D
E
F
G
H
Row-Normalized Cell-type Scores
Tabula Muris (Lung - FACS)
Mouse Cell Atlas (Lung)
Count
Resolution
Size
Proportion
UMAP 1
UMAP 2
Proliferating Clusters
Row-Normalized Cell-type Scores
MS4A1
CPA3
COL1A1
LILRA4
JCHAIN
EPAS1
HBB
CD3D
CSF3R
SFTPC
LYZ
Normalized Gene Expression
Min
Max
log10 CFU per Granuloma
Percent of Cells in Granuloma (%)
Granuloma Timing
B q = 0.63
cDC q = 0.32
Endothelial q = 0.076
Fibroblast q = 0.076
Macrophage q = 0.74
Mast q = 0.12
Neutrophil q = 0.32
pDC q = 0.89
Plasma q = 0.076
RBC q = 0.74
T/NK q = 0.076
T1P q = 0.076
T2P q = 0.5
Late
Early

Figure S2. Identification of Canonical Cell Types (related to Fig 2)

(A) Waterfall plot showing stability of cell-type clustersat multiple clustering resolutions. Boxed row (resolution=1.00) selected for downstream analysis. **(B, C)** Distribution of lung cell-type signatures obtained from the Tabula Muris (B) and Mouse cell (C) atlas. (**D**) UMAP plot of 109,584 cells colored by Louvain clusters (resolution = 1.00). **(E)** Waterfall plot showing the stability of sub-clustering analysis of 3,123 cells with a proliferating gene signature. **(F)** Distribution of canonical cell type signatures across subclusters of proliferating cells. **(G)** Expression levels of cluster-defining genes overlaid on UMAP plot in panel 2A. (**H) Left:** CFU per granuloma based on the timing of detection by PET CT scan in one animal : 4017. **Right:** Difference in granuloma proportional composition of cell type clusters between early (maroon box plot) and late granulomas (green) within an animal (4017). Each granuloma is coloured. Statistics: Mann Whitney U test with Benjamini-Hochberg multiple testing correction. Box plot showing median, IQR and range; each dot represents a granuloma.

Fig S3

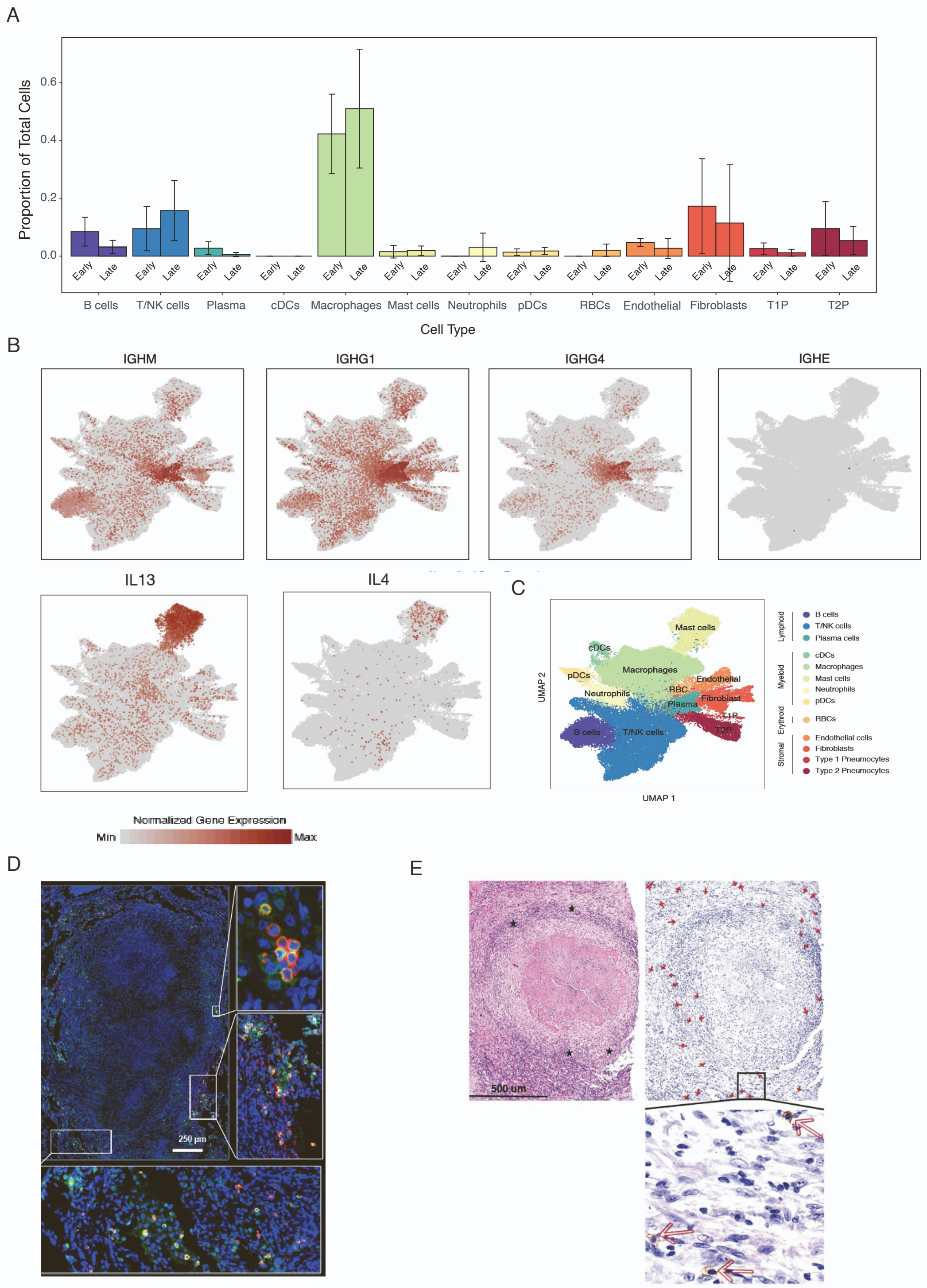

Figure S3. Cell type confirmation and Expression of selected functional transcripts (related to Fig 1-2)

(A) Proportion of cell types in granulomas from bulk sequencing of 6 early and 6 late granulomas to confirm the trend seen in scRNAseq. **(B)** UMAP plot of 109,584 cells from 26 granulomas colored by identities of 13 generic cell types. **(C)** Expression levels of select functional genes overlaid on UMAP plot of 109,584 cells. **(D)** Detection of mast cells in a 10-week NHP granuloma using immunohistochemistry, staining for tryptase (green) and c-kit (CD117)(red). **(E)** Detection of mast cells in a human lung granuloma. Hematoxylin and eosin stain and immunohistochemistry with multinucleated giant cells (stars, (top left) and c-kit (CD117) staining (indicated by arrows, top and bottom right).

Fig S4

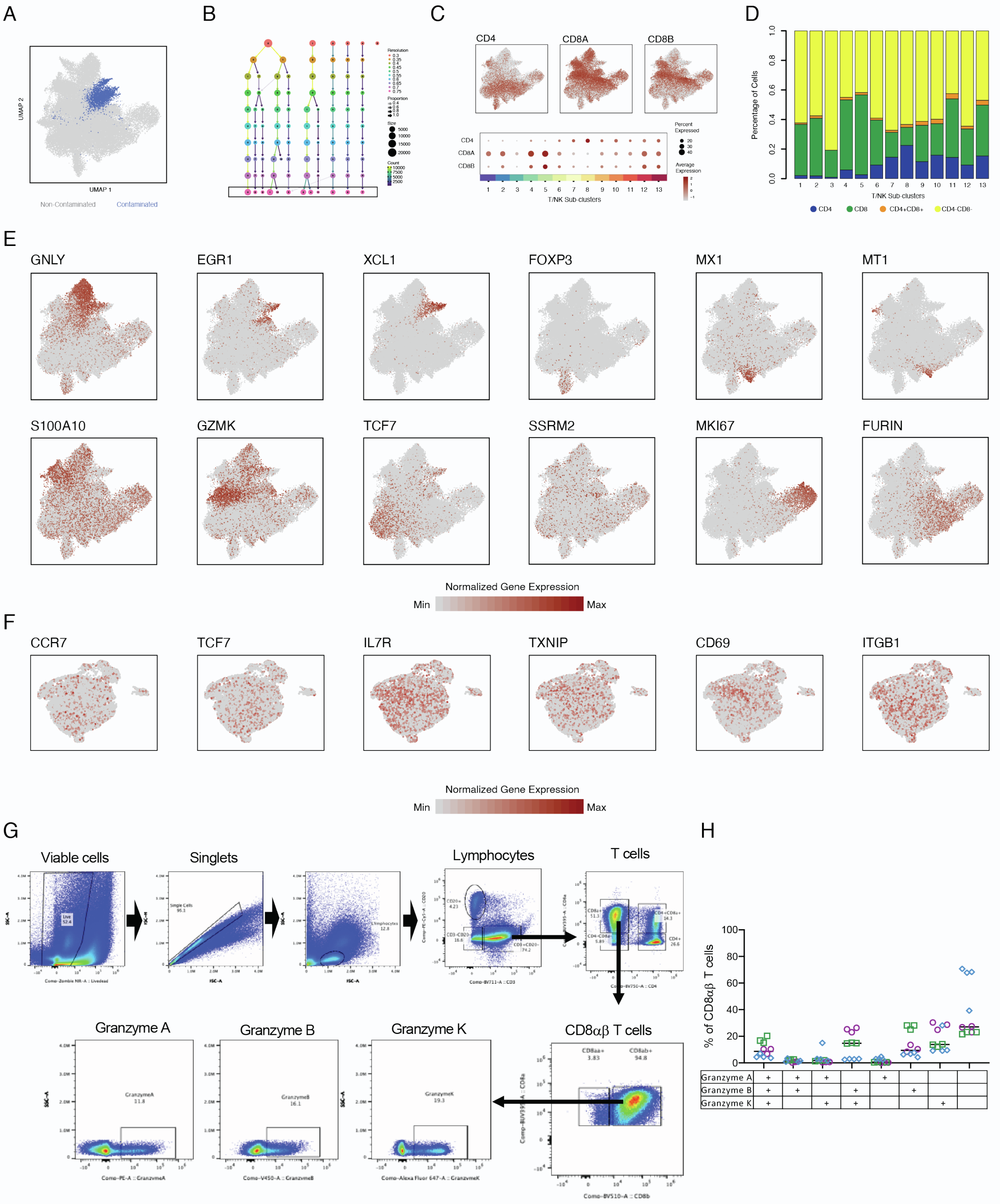

A
UMAP 2
UMAP 1
Non-Contaminated
Contaminated
B
Resolution
Proportion
Size
Count
C
CD4
CD8A
CD8B
Percent Expressed
Average Expression
T/NK Sub-clusters
1 2 3 4 5 6 7 8 9 10 11 12 13
D
Percentage of Cells
0.0 0.2 0.4 0.6 0.8 1.0
T/NK Sub-clusters
CD4
CD8
CD4+CD8+
CD4-CD8-
E
GNLY
EGR1
XCL1
FOXP3
MX1
MT1
S100A10
GZMK
TCF7
SSRM2
MKI67
FURIN
Normalized Gene Expression
Min
Max
F
CCR7
TCF7
IL7R
TXNIP
CD69
ITGB1
Normalized Gene Expression
Min
Max
G
Viable cells
Singlets
Lymphocytes
T cells
Granzyme A
Granzyme B
Granzyme K
CD8αβ T cells
H
% of CD8αβ T cells
0 20 40 60 80 100
Granzyme A + + + +
Granzyme B + + + +
Granzyme K + + + +

Figure S4. Sub-clustering and phenotypic identification of T/NK cell populations (related to Fig 3-4)

(A) UMAP plot of 44,766 T/NK cells with a sub-cluster of 3,544 T/NK cells defined by residual contamination highlighted (blue). **(B)** Waterfall plot showing the stability of T/NK cell sub-clustering following removal of contaminated T cell sub-cluster. Boxed row (resolution=0.75) selected for downstream analysis. **(C)** T/NK subclustering UMAP overlaid with normalized gene expression for CD4, CD8A, and CD8B (top). Expression of these genes across 13 sub-clusters (bottom) where color intensity corresponds to level of gene expression and size of dots represents the percent of cells with non-zero expression in each cluster. **(D)** Frequency of expression of *CD4* (blue), *CD8A* and/ *CD8B* (green), *CD4* and *CD8A/B* (orange) or no expression of *CD4/CD8A/B* (yellow) across 13 T/NK cell subclusters. **(E)** UMAP plots overlaid with normalized expression levels for selected T/NK cell subcluster-defining genes. **(F)** Expression of key T cell markers on subclustered stem-like T cell population. **(G)** Gating Tree showing identification CD8ab T cells in lung granuloma samples and population of Granzyme A, Granzyme B and Granzyme K + CD8abT cells. **(H)** Frequency of CD8ab T cells in lung granulomas making one or more (two , three) types of Granzymes (A, B or K). Each symbol is a granuloma and each colour identifies an animal. This data supports different types of granzyme producing cytotoxic cells identified in scRNAseq.

Fig S5

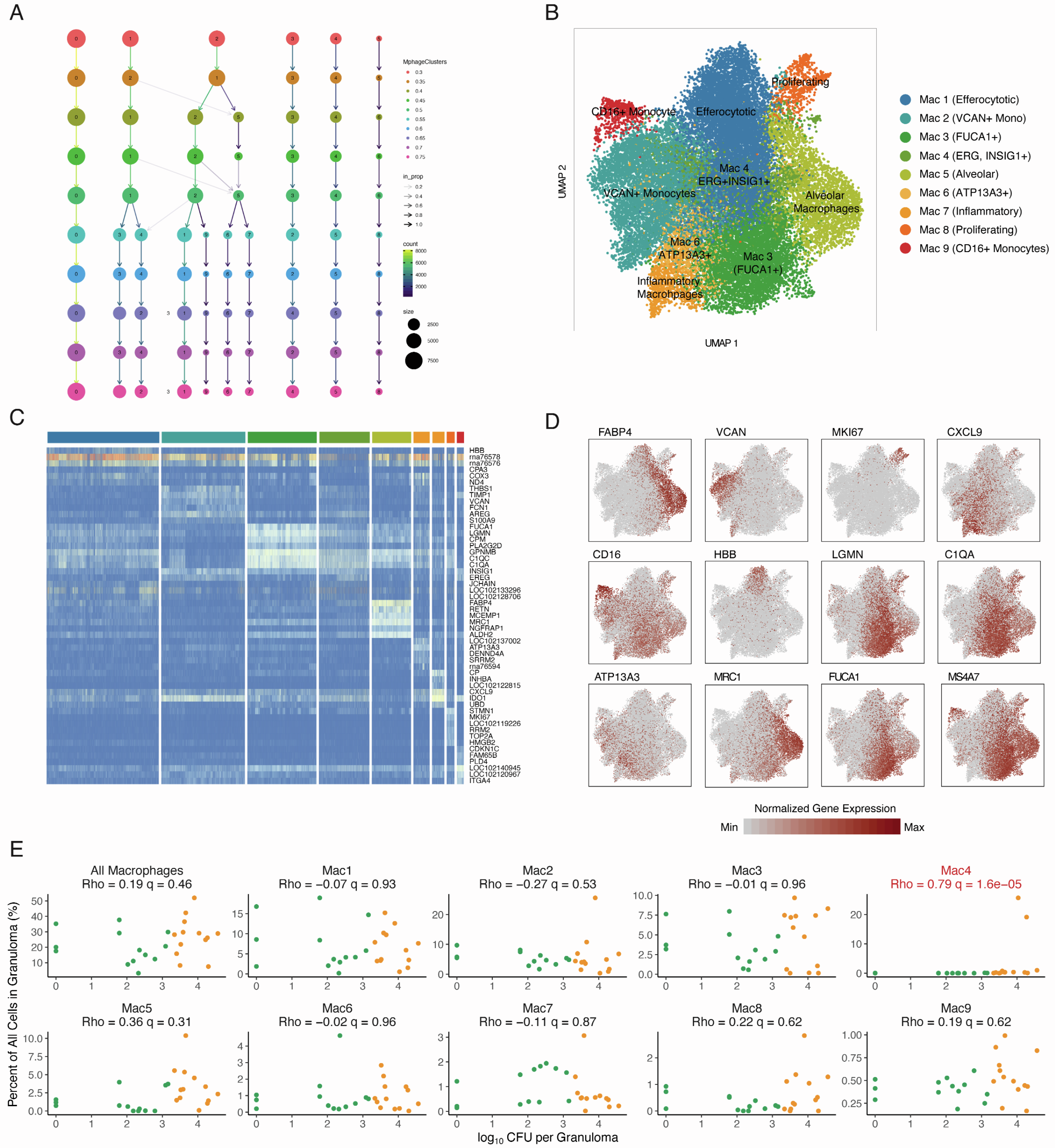

Figure S5. Macrophage heterogeneity in Mtb granulomas (related to Fig 2)

(A) Waterfall plot showing the stability of macrophage sub-clusters. Boxed row (resolution=0.55) selected for downstream analysis. **(B)** UMAP plot of 27,670 macrophage cluster colored by phenotypes. **(C)** Cluster-defining genes across macrophage subclusters. **(D)** Macrophage subcluster-defining genes overlaid on macrophage plot in panel B. **(E)** Significant correlations between proportion of Macrophage subclusters with bacterial burden of individual granulomas (Log_{10} CFU per granuloma) using non-parametric Spearman's rho correlation test. Color indicated binned granuloma bacterial burden: low (green) and high (orange).

Fig S6

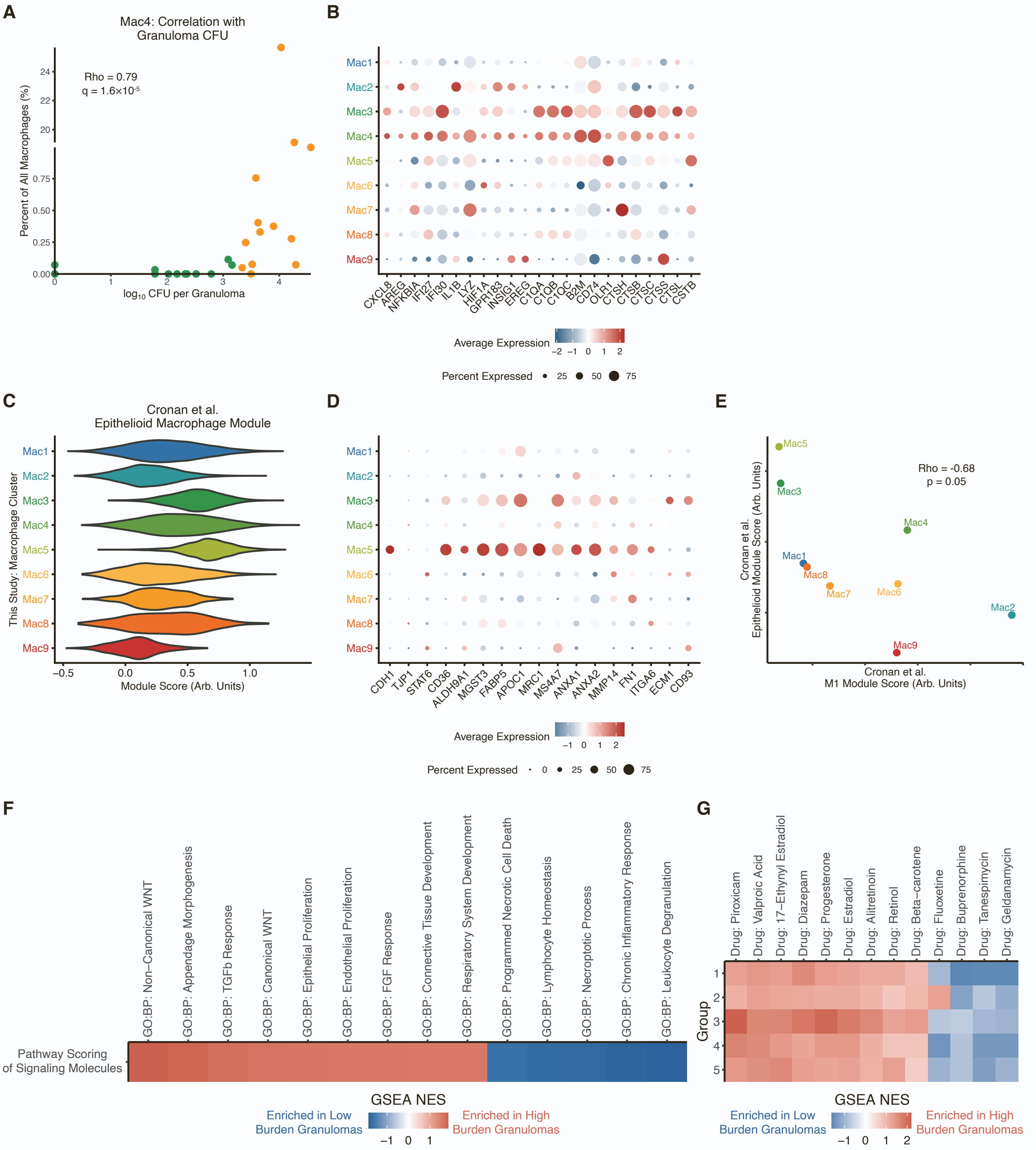
A
Mac4: Correlation with Granuloma CFU
Rho = 0.79
q = 1.6×10-5
Percent of All Macrophages (%)
log10 CFU per Granuloma
B
Mac1
Mac2
Mac3
Mac4
Mac5
Mac6
Mac7
Mac8
Mac9
CXCL8 AREG NFKBIA IFI27 IFI30 IL1B LYZ HIF1A GPR183 INSIG1 EREG C1QA C1QB C1QC B2M CD74 OLR1 CTSH CTSB CTSC CTSS CTSL CSTB
Average Expression
Percent Expressed
C
Cronan et al.
Epithelioid Macrophage Module
This Study: Macrophage Cluster
Module Score (Arb. Units)
D
CDH1 TJP1 STAT6 CD36 ALDH9A1 MGST3 FABP5 APOC1 MRC1 MS4A7 ANXA1 ANXA2 MMP14 FN1 ITGA6 ECM1 CD93
Average Expression
Percent Expressed
E
Rho = -0.68
p = 0.05
Cronan et al.
Epithelioid Module Score (Arb. Units)
Cronan et al.
M1 Module Score (Arb. Units)
F
GO:BP: Non−Canonical WNT
GO:BP: Appendage Morphogenesis
GO:BP: TGFb Response
GO:BP: Canonical WNT
GO:BP: Epithelial Proliferation
GO:BP: Endothelial Proliferation
GO:BP: FGF Response
GO:BP: Connective Tissue Development
GO:BP: Respiratory System Development
GO:BP: Programmed Necrotic Cell Death
GO:BP: Lymphocyte Homeostasis
GO:BP: Necroptotic Process
GO:BP: Chronic Inflammatory Response
GO:BP: Leukocyte Degranulation
Pathway Scoring of Signaling Molecules
GSEA NES
Enriched in Low Burden Granulomas
Enriched in High Burden Granulomas
G
Drug: Piroxicam
Drug: Valproic Acid
Drug: 17−Ethynyl Estradiol
Drug: Diazepam
Drug: Progesterone
Drug: Estradiol
Drug: Alitretinoin
Drug: Retinol
Drug: Beta−carotene
Drug: Fluoxetine
Drug: Buprenorphine
Drug: Tanespimycin
Drug: Geldanamycin
Group
GSEA NES
Enriched in Low Burden Granulomas
Enriched in High Burden Granulomas

Figure S6. Connecting cell states and interactions to prior knowledge (related to Fig 2, 6)

(A) Correlation between Mac4 compositional abundance and granuloma burden, using Spearman's rho correlation test with Benjamini-Hochberg correction for multiple hypothesis testing. **(B)** Expression levels marker genes that align the Mac4 subcluster with pro-inflammatory, interferon-responsive macrophages, as in Esaulova et al. Color intensity corresponds to level of gene expression, and size of dots represents the proportion of cells with non-zero expression in each cluster. **(C)** Module scoring of macrophage clusters from this study against markers associated with Cronan et al.'s epithelioid macrophage populations. **(D)** Expression levels marker genes that align the Mac3 and Mac5 subclusters with epithelioid, pro-remodeling macrophage phenotypes, as in Cronan et al. Color intensity corresponds to level of gene expression, and size of dots represents the proportion of cells with non-zero expression in each cluster. **(E)** Module scoring of macrophage clusters from this study against M1 and epithelioid gene modules from Cronan et al; Spearman's rho correlation test. **(F)** Pathways enriched in signaling molecules associated with high vs. low granuloma burden. Signaling molecules were ranked according to their log(fold-change in high vs. low burden granulomas) as input to GSEA. **(G)** Drugs with targets enriched in signaling molecules associated with each cell type group.
